# Human genetic history on the Tibetan Plateau in the past 5100 years

**DOI:** 10.1126/sciadv.add5582

**Published:** 2023-03-17

**Authors:** Hongru Wang, Melinda A. Yang, Shargan Wangdue, Hongliang Lu, Honghai Chen, Linhui Li, Guanghui Dong, Tinley Tsring, Haibing Yuan, Wei He, Manyu Ding, Xiaohong Wu, Shuai Li, Norbu Tashi, Tsho Yang, Feng Yang, Yan Tong, Zujun Chen, Yuanhong He, Peng Cao, Qingyan Dai, Feng Liu, Xiaotian Feng, Tianyi Wang, Ruowei Yang, Wanjing Ping, Zhaoxia Zhang, Yang Gao, Ming Zhang, Xiaoji Wang, Chao Zhang, Kai Yuan, Albert Min-Shan Ko, Mark Aldenderfer, Xing Gao, Shuhua Xu, Qiaomei Fu

**Affiliations:** ^1^ Key Laboratory of Vertebrate Evolution and Human Origins, Institute of Vertebrate Paleontology and Paleoanthropology, Center for Excellence in Life and Paleoenvironment, Chinese Academy of Sciences, Beijing 100044, China.; ^2^ Department of Biology, University of Richmond, Richmond, VA 23173, USA.; ^3^ Tibet Institute for Conservation and Research of Cultural Relics, Lhasa 850000, China.; ^4^ School of Archaeology and Museology, Sichuan University, Chengdu 610064, China.; ^5^ Center for Archaeological Science, Sichuan University, Chengdu 610064, China.; ^6^ School of Cultural Heritage, Northwest University, Xi’an 710069, China.; ^7^ Key Laboratory of Western China’s Environmental Systems (Ministry of Education), College of Earth and Environmental Sciences, Lanzhou University, Lanzhou 730000, China.; ^8^ University of the Chinese Academy of Sciences, Beijing 100049, China.; ^9^ School of Archaeology and Museology, Peking University, Beijing 100871, China.; ^10^ State Key Laboratory of Genetic Engineering, Center for Evolutionary Biology, Collaborative Innovation Center of Genetics and Development, School of Life Sciences, Fudan University, Shanghai 200438, China.; ^11^ Key Laboratory of Computational Biology, Shanghai Institute of Nutrition and Health, University of Chinese Academy of Sciences, Chinese Academy of Sciences, Shanghai 200031, China.; ^12^ Department of Anthropology and Heritage Studies, University of California, Merced, Merced, CA 95343, USA.; ^13^ Department of Liver Surgery and Transplantation, Liver Cancer Institute, Zhongshan Hospital, Fudan University, Shanghai 200032, China.; ^14^ Human Phenome Institute, Zhangjiang Fudan International Innovation Center, and Ministry of Education Key Laboratory of Contemporary Anthropology, Fudan University, Shanghai 201203, China.; ^15^ Shanghai Qi Zhi Institute, Shanghai 200232, China.

## Abstract

Using genome-wide data of 89 ancient individuals dated to 5100 to 100 years before the present (B.P.) from 29 sites across the Tibetan Plateau, we found plateau-specific ancestry across plateau populations, with substantial genetic structure indicating high differentiation before 2500 B.P. Northeastern plateau populations rapidly showed admixture associated with millet farmers by 4700 B.P. in the Gonghe Basin. High genetic similarity on the southern and southwestern plateau showed population expansion along the Yarlung Tsangpo River since 3400 years ago. Central and southeastern plateau populations revealed extensive genetic admixture within the plateau historically, with substantial ancestry related to that found in southern and southwestern plateau populations. Over the past ~700 years, substantial gene flow from lowland East Asia further shaped the genetic landscape of present-day plateau populations. The high-altitude adaptive *EPAS1* allele was found in plateau populations as early as in a 5100-year-old individual and showed a sharp increase over the past 2800 years.

## Teaser

Ancient human DNA from the last 5,100 years provides insight into Tibetan ancestry and population dynamics on the plateau.

## INTRODUCTION

The Tibetan Plateau, with elevations often exceeding 4000 m above sea level (masl), is one of the most inhospitable places ever inhabited by humans. Archaic humans known as Denisovans were present on the northeastern regions of the plateau by at least 160,000 years (*1*), and early modern humans appeared on the central plateau at least 40,000 to 30,000 years ago (*2*). However, the permanent settlement of modern humans on the plateau (*3*, *4*) and the relationship of present-day Tibetans to early occupants of the plateau (*5*) are much debated.

The origin of present-day Tibetans is of high interest, and genetic studies of present-day Tibetans in the past decade have revealed a close genetic relationship to their lowland East Asian counterparts. However, some of these studies suggest that more deeply diverged populations likely also contributed to the ancestral gene pool of Tibetans today (*6*, *7*). Ancient DNA from past humans living in the Himalayan arc on the southwest edge of the Tibetan Plateau has shown that humans in this region dating back to 3400 years share a unique ancestry with present-day Tibetans. The ancient populations in the Himalayan arc and all present-day plateau populations are closely related to ancient populations of northern East Asian ancestry found in the Yellow and Amur River Valley regions, and all show a small amount of deeply diverged ancestry that has not yet been directly sampled (*8*, *9*).

While these studies have revealed that a shared ancestry can be traced from widespread plateau populations back to the Himalayan arc of the Tibetan Plateau in Nepal as early as 3400 years ago, a number of important questions remain. First, no ancient modern humans older than 3400 years have been sampled genetically, making it unclear when this shared ancestry first entered the plateau population. Second, the extent to which this shared ancestry is represented in ancient populations from other regions of the plateau is unclear. Without further spatial resolution, it is difficult to resolve why the ancient populations in the Himalayan arc and widespread present-day plateau populations share similar ancestry. Historical records suggest that the earliest complex societies on the Tibetan Plateau likely began ~2500 years ago (i.e., Zhang Zhung), which was conquered by the Tibetan Empire that spanned from ~1300 to 1100 years ago (629 to 842 CE) (*10*, *11*). How these political shifts affected human migration and interaction on the plateau is unknown.

To address these questions, we sampled DNA from ancient humans who once lived on the Tibetan Plateau, surveying across a broad temporal and spatial range throughout the Tibet Autonomous Region and Qinghai province of China. Notably, this includes 21 individuals dated to 5100 to 3900 years ago from the northeastern edges of the Tibetan Plateau, extending our ability to investigate the genetic history of humans on the Tibetan Plateau back by another 1700 years.

## RESULTS

**Fig. 1.**
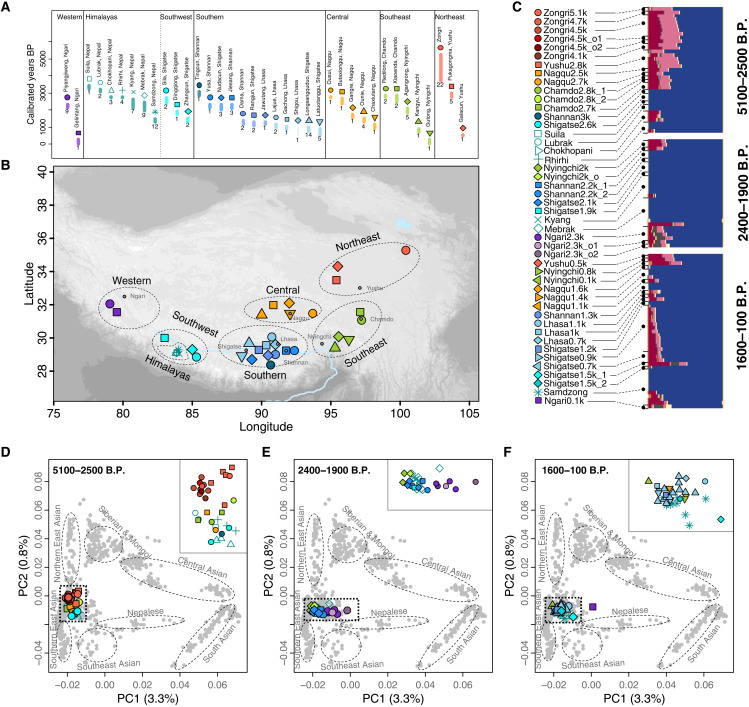
Chronological, geographic distribution and genetic landscape of ancient individuals of the Tibetan Plateau. (**A**) Dating and number of individuals sampled per site. Thick lines show the 95% confidence interval (CI) for radiocarbon dating for all samples at a site, and thin lines denote dates estimated from archaeological contexts. Asterisks denote previously published individuals. Colors indicate the seven regions of the Tibetan Plateau in which each site is grouped, as well as denoted on the map. (**B**) Geographic location of each site, with the seven regions given by the dashed circles and color. Gray dots denote the capitols of the associated prefectures on the Tibetan Plateau, written in gray. (**C**) ADMIXTURE plot for ancient Tibetans at *K* = 11. Blue, Tibetan ancestry; red, southern East Asian ancestry; pink, Ancient Northern Siberian ancestry; yellow/gray, Central Asian ancestry. Full ADMIXTURE results can be found in fig. S14. (**D** to **F**) PCA of present-day populations on or near the Tibetan Plateau (gray dots) onto which ancient individuals were projected. The insets represent the black dotted square, and they zoom in on the distribution of ancient Tibetan individuals. Dashed circles indicate the broader categories into which present-day populations can be placed, labeled in gray. The corresponding present-day populations associated with these categories can be found in fig. S12.

We generated genome-wide data at ~1.2 million single-nucleotide polymorphisms (SNPs) for 97 ancient human specimens from 30 archaeological sites across the Tibetan Plateau with elevations ranging from 2776 to 5000 masl (Fig. 1B, fig. S1, and table S1). Direct radiocarbon dating for 62 individuals shows that they lived from 5260 to 27 calibrated years before the present (1950 CE) (cal B.P.) (Fig. 1A, fig. S1, and table S2). After removing samples for contamination and a low number of SNPs (<10,000) (table S1), 89 individuals from 29 sites were retained with contamination levels lower than 3.50% (tables S1 and S3). Across the 89 individuals, the sequencing depth at the 1.2 million SNPs ranged from 0.01 to 9.81×, and the number of SNPs covered with at least one read ranged from 14,552 to 1,120,464 (table S1).

To determine whether any individuals sampled were related to each other, we performed a kinship analysis (*12*). Of the 89 individuals, 14 familial groups (first- and second-degree) were found (table S4). The individual with the highest SNP count was retained for each group (table S1), resulting in 75 unrelated individuals for population genetic analysis. To our panel, we added 33 published individuals from the Himalayan arc on the southern margin of the Tibetan Plateau in Nepal, which date to 3440 to 1250 B.P. (*8*, *9*). We compared these ancient plateau individuals to previously published present-day and ancient humans from the surrounding regions, particularly those from East Asia, Central Asia, and Siberia due to their proximity to the Tibetan Plateau.

### Formation of Tibetan ancestry dates back to at least 5100 B.P.

To address the genetic relationship between the sampled individuals and present-day Asian populations, we performed principal components analysis (PCA) (*13*). After projecting ancient plateau individuals onto the first two principal components constructed with either diverse present-day Asian populations (Fig. 1, D to F, and fig. S12) or only present-day East and Southeast Asians (fig. S13), we found that ancient plateau individuals consistently overlap with or fall close to the Qiang, Tibetan, and Sherpa who live on or near the Tibetan Plateau today. These three present-day populations also share the most alleles with ancient plateau individuals in outgroup *f*_3_ analyses (figs. S2 to S11) (*14*). Collectively, these results suggest that the ancient individuals dating to 5100 to 300 years B.P. spanning from the northeastern to the southern regions of the Tibetan Plateau all share the closest genetic relationship to human populations that live on or near the Tibetan Plateau today.

**Fig. 2.**
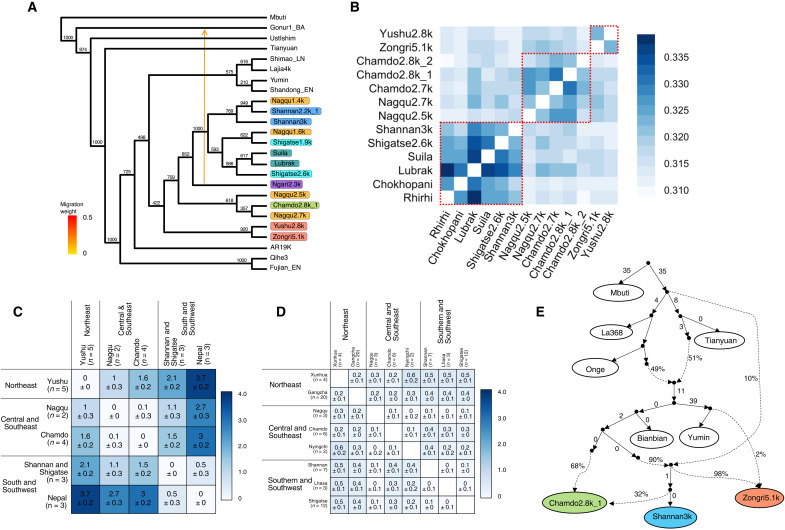
Population structure in ancient Tibetans. (**A**) Maximum likelihood phylogeny with one migration edge inferred with TreeMix. Colors indicate geographic location on the Tibetan Plateau: pink, northeast; blue, south and southwest; orange, central; green, southeast; purple, west. Numbers indicate bootstrap support from 1000 replicates. Original phylogeny with branch lengths can be found in fig. S19. (**B**) Outgroup *f*_3_ analysis for *f*_3_ (X, Y; Mbuti) where Mbuti is a central African present-day population and X and Y are Early Ancient Tibetans. Darker blue indicates higher genetic similarity. (**C** and **D**) Pairwise *F*_ST_ analysis with SEs (×100) for Early Ancient Tibetans (C) and present-day Tibetans (D) from three plateau regions. Darker blue indicates higher genetic differentiation. (**E**) Admixture graph inferred with qpGraph. Dashed lines denote admixture events, and colored boxes represent ancient Tibetan Plateau individuals, where colors denote their corresponding geographic location. The largest magnitude for the *Z* scores comparing all expected *f* values to observed *f* values is 2.32.

To evaluate the genetic origins of Tibetan Plateau populations, we next focused on the earliest plateau individuals sampled to date (5100 to 2500 B.P., “Early Ancient Tibetans”), whose locations span the full range of Tibetan Plateau from the Himalayan arc on the west to the Gonghe Basin on the northeast (Fig. 1, B and D). To determine their relationship with temporally and geographically diverse populations across Asia, we estimated a maximum likelihood phylogeny (*15*) comparing ancient plateau individuals to 45,000- to 3000-year-old individuals from across Asia, we found that all Early Ancient Tibetans cluster with 19,000- to 4500-year-old East Asians from low elevation regions of east of the plateau [bootstrap support (b.s.) = 1000/1000; Fig. 2A and fig. S19]. However, they are more closely related to 9500- to 4000-year-old northern East Asians from the Yellow River region than 12,000- to 8000-year-old southern East Asians from coastal southern China (b.s. = 725/1000; Fig. 2A and fig. S19). In an *f*_4_ analysis (*14*), Early Ancient Tibetans from the northern, southern, and central Tibetan Plateau share more alleles with a diverse set of ancient northern East Asians than ancient southern East Asians from coastal southern China, i.e., *f*_4_ (Ancient Northern East Asian, Ancient Southern East Asian; Early Ancient Tibetans, Mbuti) < 0 (1.7 < *Z* < 8.6; fig. S16). In an outgroup *f*_3_ analysis comparing to diverse lowland ancient Asian individuals, the oldest plateau individual sampled (Zongri5.1k) shared the highest genetic drift with other ancient individuals from the plateau, followed by ancient northern East Asians (fig. S17). These patterns support that early humans on the Tibetan Plateau dating from 5100 to 2500 years ago are closely related to ancient northern East Asians.

To address the genetic formation of the unique ancestry found on the plateau, we performed qpAdm analysis (*16*–*18*), which tests whether a target population can be described as a mixture of ancestries related to predefined source populations for Early Ancient Tibetans with distal sources. We confirmed that the primary ancestral sources in Early Ancient Tibetans are related to one or more ancient northern East Asians (>74% northern East Asians related ancestry; table S14). The remaining ancestry (7 to 26%) is attributable to an ancestry deeply diverged from Asian ancestries sampled to date (table S14) and is related to one or more of the following sources: a 45,000-year-old Ust’-Ishim individual from Siberia, a 40,000-year-old Tianyuan individual from northern China, or present-day Andamanese islanders (Onge; table S14). This ancestry is unlikely to be related to archaic humans, as no elevated archaic-related ancestry is detected in Early Ancient Tibetans (tables S18 and S20). Similar patterns were also found in other studies on present-day and ancient plateau populations from the Himalayan arc (*6*–*9*, *19*). The similar mixture proportion pattern suggests that the ancestry across plateau populations derive from a single common origin. The maximum likelihood phylogeny estimated does not show strong support at first glance for a single shared ancestry between ancient plateau populations (b.s. = 422/1000; Fig. 2A), but the low support is primarily attributable to some Early Ancient Tibetans on occasion being placed as more deeply diverged than one or more ancient lowland East Asians. When allowing a couple of ancient plateau individuals to move out of the plateau-related clade, node support increase substantially (b.s. = 944/1000; see Supplementary Text for “TreeMix analysis”). A qpGraph analysis shows shared ancestry across geographically diverse Early Ancient Tibetans that derives from a single source related to a 9500-year-old individual from the Yellow River region (68 to 90%; Fig. 2E), but similarly, attempts to model deeply diverged ancestry as contributing to all plateau populations around their origin do not fit. In both cases, the lack of an ancient individual representing the deeply diverged ancestry in ancient plateau populations makes it difficult to successfully model how this ancestry first entered early plateau populations. An *f*_4_ analysis helps to establish that despite deeply diverged ancestry making phylogenetic analysis and qpAdm modeling difficult, there is substantial support for a closer genetic relationship between Early Ancient Tibetans than with 19,000- to 12,000-year-old northern and southern East Asians, i.e., most *f*_4_ (Early Ancient Tibetan, Qihe3/AR19K; Early Ancient Tibetan, Mbuti) > 0 (table S9A). Overall, we find that plateau-related ancestry was probably formed through the mixture of a major source related to ancient northern East Asians and a minor source from a “ghost” population not yet sampled (Fig. 2E and fig. S33). Finding this pattern in ancient plateau individuals shows that this mixed ancestry was widespread across prehistoric populations of the plateau and dates back as far as 5100 years ago, predating the introduction of wheat and barley to the Tibetan Plateau (*3*).

The 4200-year-old individuals from the Lajia archaeological site who lived in the Upper Yellow River Valley were close neighbors of the northeastern plateau region, and the Lajia population was thought to be important in the formation of plateau-related ancestry (*9*). Our phylogenetic analysis shows that 4200-year-old Lajia individuals do not group with Early Ancient Tibetans but instead group with ancient northern East Asians (Fig. 2A). In an *f*_4_ analysis comparing the Lajia individuals to a broad sample of ancient northern East Asians, we found that they and ancient northern East Asians from Inner Mongolia and the Yellow River Valley are similarly related to most Early Ancient Tibetans, and the Lajia individuals form a clade with other contemporary populations along the Yellow River (Miaozigou_MN and Shimao_LN) relative to Early Ancient Tibetans (table S8). Moreover, in a rotating qpAdm analysis allowing potential sources including the Lajia population, the previously published 3400-year-old Lubrak individuals from the southern plateau region, the 5100-year-old Zongri individual, and a diverse array of ancient northern East Asians, we found that most Early Ancient Tibetans can be described as solely or primarily ancestry related to the Lubrak or Zongri individuals (table S15), and most reject Lajia4k as a potential source (table S15). These patterns show that Early Ancient Tibetans carry a unique ancestry distinct from that observed in populations from the upper reaches of the Yellow River. We denote this as Tibetan ancestry.

### Population structure on the Tibetan Plateau reveals three local ancestries before 2500 B.P.

Most present-day Tibetan populations living on the plateau today share a close genetic relationship (*6*, *7*), and studies comparing 3400- to 1200-year-old individuals from Nepal to present-day Tibetans reveal high genetic continuity (*8*, *9*). With spatially diverse sampling, we next investigated the population structure within ancient plateau populations. Comparing the levels of genetic differentiation within Early Ancient Tibetans to the levels within present-day Tibetans using an *F*_ST_ analysis (*20*), we found that there is much higher differentiation between Early Ancient Tibetans across different regions (*F*_ST_ = 0.01 to 0.037; Fig. 2C) than that between present-day Tibetans (*F*_ST_ = 0 to 0.005; Fig. 2D). We used an outgroup *f_3_* analysis (*14*, *21*) to examine genetic clustering within Early Ancient Tibetans and found that they could be separated into at least three genetic clusters based on pairwise shared genetic drift (Fig. 2B). These three clusters follow a geographic pattern, including (i) a “northeast” cluster of 5100- to 2800-year-old individuals from the Yushu prefecture and the Zongri archaeological site in the Gonghe Basin in the Hainan prefecture of Qinghai province; (ii) a “southeast-central” cluster of 2800- to 2500-year-old individuals from the Chamdo and Nagqu prefectures; and (iii) a “south-southwest” cluster of 3400- to 2600-year-old individuals from the Shannan and Shigatse prefectures of the Tibet Autonomous Region, which also includes previously published individuals (*8*, *9*) from the Himalayan arc (Fig. 2B). Early Ancient Tibetans in each cluster group most closely with each other, with bootstrap support of 100% for the south-southwest cluster, 92% for the northeast cluster, and 61.8% for the southeast-central cluster (Fig. 2A), which is also consistent with *f*_4_ (table S9C) and outgroup *f*_3_ statistics (Fig. 2B and fig. S17), lending stronger cladal support for northeast and southern clusters, while the southeast-central cluster shows an intermediate pattern between the two clusters. The genetic relationships from the *f*_4_ analyses between ancient plateau populations of different clusters are not as robust relative to ancient lowland northern East Asians (table S9), which suggests that the population structure occurred fairly soon after their separation from other northern East Asians. We did not observe a significant correlation between longitude and genetic relationships to lowland East Asians in Early Ancient Tibetans (*P* > 0.41, Mantel test; fig. S27), which was observed in present-day Tibetans (*22*). The patterns within Early Ancient Tibetans support that the unique Tibetan ancestry was deeply diverse, giving rise to three different ancestral patterns before 2500 B.P.: a northeastern plateau ancestry associated with the northeast cluster, a southern plateau ancestry associated with the south-southwest cluster, and a southeastern plateau ancestry associated with the southeast-central cluster.

### Gene flow in lower elevation regions of the northeastern Tibetan Plateau from 4700 B.P.

The northeastern region of the Tibetan Plateau has been considered a major interaction zone for different human populations during the Neolithic, as changes in pottery style, human diet, and burial patterns indicate cultural contact between the local foragers associated with the region and millet farmers from further east (*23*–*25*). The northeast cluster of Early Ancient Tibetans includes the oldest human sampled from the Tibetan Plateau at 5100 B.P. and is represented by humans from two archaeological sites: 12 unrelated individuals dating to 5100 to 3900 B.P. from the Zongri site in the Gonghe Basin of Qinghai province and 5 unrelated individuals dating to 2800 B.P. from the Pukagongma site in the Yushu prefecture of Qinghai province (Fig. 1, A and B). We used these individuals to investigate whether human migration and admixture also played a role in cultural interactions and shifts previously observed (*24*, *25*).

**Fig. 3.**
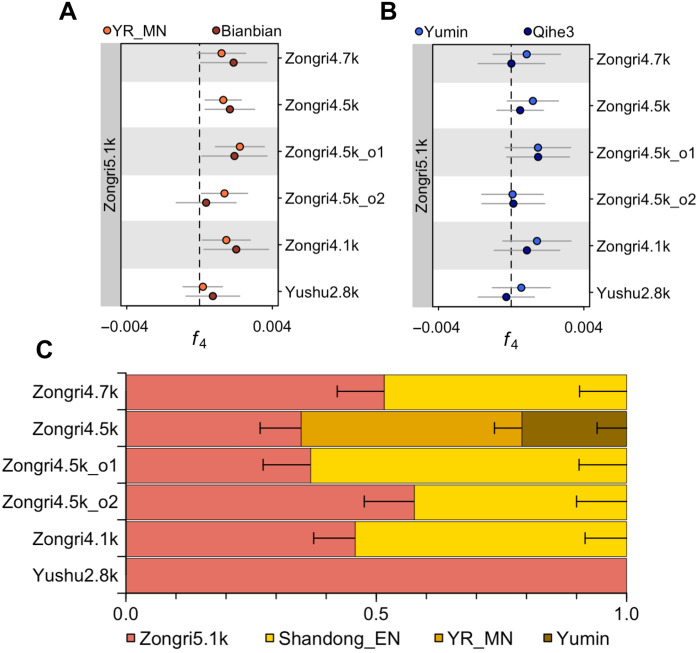
Genetic interaction on the northeastern Tibetan Plateau. (**A** and **B**) Symmetry test between Zongri5.1k and later ancient populations on the northeastern Tibetan Plateau. The statistics *f*_4_ (Zongri and Yushu individuals, Zongri5.1k; X, Mbuti) are shown with ±3 SEs, where X includes Early Neolithic individuals from the Yellow River region carrying northern East Asian ancestry in (A) and Early Neolithic individuals from Inner Mongolia and coastal southern China in (B). (**C**) Genetic ancestry estimated with qpAdm. The local Zongri5.1k-related ancestry is denoted in pink, and Neolithic northern East Asian–related ancestry is denoted by different shades of yellow. The error bars represent ±1 SE. Mixture models with the highest *P* values are shown, and all feasible models for each population are listed in table S15.

Focusing first on the Zongri site, we found that the 5100-year-old individual showed a distinct genetic pattern from younger individuals (table S7 and fig. S18). In an ADMIXTURE (*26*) analysis, the 4700- to 3900-year-old Zongri individuals harbor an ancestral component that is maximized in present-day southern East Asians, while the 5100-year-old individual (Zongri5.1k/C4783_C202) does not show this component (Fig. 1C, figs. S14 and S15, and table S5). In an *f*_4_ analysis, the 4700- to 4100-year-old Zongri individuals share significantly more alleles with Neolithic populations from the Yellow River region than the 5100-year-old individual (2.7 < |*Z*| < 4.9; Fig. 3, A and B). We further investigated potential sources of gene flow using qpAdm, and we found that the younger Zongri individuals are best described as a mixture of ancestry related to the 5100-year-old Zongri individual (40 to 73%) and Early and Middle Neolithic populations from Inner Mongolia (Yumin) and the Yellow River region (YR_MN and Shandong_EN; Fig. 3C and table S15). Collectively, these patterns show that additional northern East Asian ancestry affected populations at the Zongri site 4700 years ago, supporting that gene flow accompanied the cultural interactions that occurred in this region.

The other members of the northeast cluster derive from the Pukagongma site, located 300 km west of Zongri at a higher elevation region in the Yushu prefecture of the Tibetan Plateau. Unlike the younger Zongri individuals (4700 to 3900 B.P.), the 2800-year-old Pukagongma individuals from Yushu share a similar number of alleles to Neolithic northern East Asians, i.e., *f*_4_ (Yushu2.8k, Zongri5.1k; Neolithic northern East Asians, Mbuti) ~ 0 (0.2 < |*Z*| < 1.8; Fig. 3, A and B). qpAdm analysis shows that they can be described as deriving from the same ancestral population as the 5100-year-old Zongri individual (*P* = 0.08, chi-square test; table S15). These patterns suggest that while migration from nonlocal populations greatly affected populations who lived in the Gonghe Basin such as those who lived at the Zongri site, these external impacts did not reach the neighboring, higher-altitude region of the Tibetan Plateau in the Yushu prefecture by 2800 B.P.

### Onset of southern plateau ancestry along the Yarlung Tsangpo River before 3400 B.P.

The Yarlung Tsangpo River (*27*) is the greatest river on the Tibetan Plateau, with its headwaters in the Ngari prefecture from the western regions of the plateau. It spans across the entire southern plateau, running west to east, from the Ngari prefecture through the Shigatse, Shannan, and Lhasa prefectures of the southwestern and southern plateau, and out through the Nyingchi prefecture in the southeastern plateau (Fig. 1B). The Yarlung Tsangpo River valley is one of the places where prehistoric humans were found on the plateau (*4*, *5*), and this valley is also the most populated region of the plateau today. Early Ancient Tibetans that span the southwestern and southern prefectures along this river valley, such as the 3400- to 2600-year-old individuals from the Shannan, Shigatse, and Himalayan arc (Fig. 1B), form a robust clade in a phylogenetic analysis (Fig. 2A). They share more alleles with each other than they share with Early Ancient Tibetans from other clusters (table S12). qpWave analysis using reference populations with the power to distinguish different plateau ancestries further shows that they can be described as deriving from the same stream of ancestry (*P* > 0.06, chi-square test; table S16). These results show that they all harbor a homogenous southern plateau ancestry, which is probably underpinned by the spread of an ancestral population carrying southern ancestry who possibly traveled upstream from the east to the west along the river valley before 3400 B.P.

**Fig. 4.**
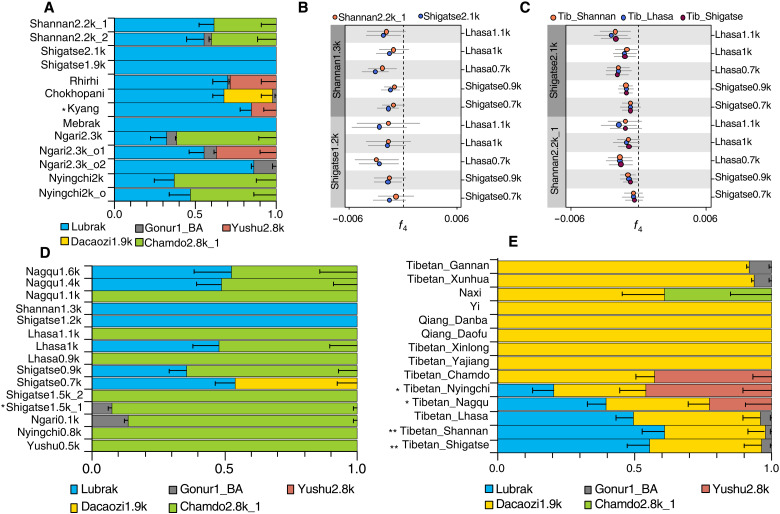
Genetic transformations on the Tibetan Plateau over the past ~3000 years. (**A**) Genetic ancestry of 3000- to 1900-year-old individuals from the southern and southwestern regions of the plateau using qpAdm. (**B**) Symmetry test comparing southern and southwestern Tibetans dating from 2200 to 2100 B.P., 1300 to 1200 B.P., and 1100 to 900 B.P. to each other, using *f*_4_ (1100 to 900 B.P., 1300 to 1200 B.P.; 2200 to 2100 B.P., Mbuti). (**C**) Symmetry test comparing southern and southwestern Tibetans dating from 2200 to 2100 B.P., 1100 to 900 B.P., and the present-day to each other, using *f*_4_ (1100 to 900 B.P., 2200 to 2100 B.P.; present-day, Mbuti). (**D** and **E**) Genetic ancestry of 1600- to 100-year-old individuals (D) from the southern and southwestern regions of the plateau and present-day plateau populations (E) using qpAdm. For (A), (D), and (E), mixture models with the highest *P* values are shown, and all feasible models for each population are listed in table S17. The qpAdm modeling was performed using “O10” outgroup and five populations representing different ancestries. 0.01 < *P < 0.05 and **P < 0.01. Error bars represent ±1 SE.

To further document the geographic span of the southern plateau ancestry, we also examined the oldest individuals sampled to date at the start and end of the river on the Tibetan Plateau (i.e., the southeastern and western plateau). In the Ngari prefecture on the western plateau, the oldest individuals date to 2300 B.P. and were excavated from the Piyangjiweng archaeological site. In a phylogenetic analysis, they form a clade with individuals belonging to the south-southwest cluster (b.s. = 85.2%; Fig. 2A). Mixture models show that these 2300-year-old Ngari individuals carry 32 to 86% southern plateau ancestry (Fig. 4A and table S17). On the southeastern plateau, we analyzed the earliest individuals from the Nyingchi prefecture, i.e., three individuals dating to ~2000 B.P. from the Agangrong site. In an outgroup *f*_3_ analysis, they shared the highest genetic similarity with 2600- to 2200-year-old individuals from the Shannan and Shigatse prefectures (fig. S22). In a proximal qpAdm analysis, they are a mixture of southern (37 to 47%) and southeastern (53 to 63%) plateau ancestries (Fig. 4A and table S17). Therefore, our analyses show that southern plateau ancestry shaped the southern and southwestern plateau populations by 3400 B.P. and was widespread along the entire Yarlung Tsangpo River valley by 2000 B.P. The wide span of the southern plateau ancestry is likely related to prehistoric human migration up the river, possibly facilitated by favorable environmental conditions for humans along this river valley (*27*, *28*).

### Genetic continuity and fluctuations on the south and southwest Tibetan Plateau since 3400 B.P.

The southern and southwestern Tibetan Plateau (Fig. 1B) is the cultural heartland of the plateau (*29*). To trace the population dynamics in this region further, we analyzed an additional 31 individuals spanning 2200 to 700 years ago from the Lhasa, Shannan, and Shigatse prefectures (table S1). In outgroup *f*_3_ analyses, the 2200- to 1900-year-old individuals from Shannan and Shigatse show the most genetic similarity to Early Ancient Tibetans since 3400 years ago from the south-southwest cluster (fig. S21), consistent with a phylogenetic analysis supporting that they form a clade with local Early Ancient Tibetans (Fig. 2A). Aside from a 2200-year-old individual from Shannan, individuals from these two prefectures spanning 3000 to 1900 years B.P. can be described as sharing the same ancestry (*P* > 0.1, chi-square test; table S16) in a qpWave analysis. In a proximal qpAdm analysis, they carry 55 to 100% southern plateau ancestry related to Lubrak (*P* > 0.07; Fig. 4A and table S17). These results show continuity in genetic ancestry in the southern and southwestern plateau over 3400 to 1900 B.P.

From 1500 to 700 years B.P., all groups except one outlier (Shigatse1.5k_1) share the highest genetic drift with preceding populations from the region (fig. S24), suggesting population continuity. However, a closer examination shows that fluctuations in genetic ancestry did occur during this period. First, on the southern Tibetan Plateau, 2200- to 2100-year-old individuals share more alleles with 1300- to 1200-year-old individuals than with 1100- to 700-year-old individuals, i.e., *f*_4_ (1300 to 1200 B.P. populations, 1100 to 700 B.P. populations; 2200 to 2100 B.P. populations, Mbuti) > 0 (1.2 < *Z* < 6.4; Fig. 4B), indicating that the 1100- to 700-year-old population might have been affected by ancestry unrelated to the south-southwest cluster. In a qpAdm analysis, the 1300- to 1200-year-old individuals do not show additional admixture, but most 1100- to 700-year-old individuals show ancestry-related to 2800- to 2000-year-old individuals from other regions (Fig. 4D and table S17), suggesting cross-regional population admixture occurred during this period.

Present-day Tibetans from the Shannan, Shigatse, and Lhasa prefectures share more alleles with 2200- to 2100-year-old individuals than they share with 1100- to 700-year-old individuals from the southern and southwestern plateau, i.e., *f*_4_ (2200 to 1200 B.P. populations, 1100 to 700 B.P. populations; present-day Tibetans, Mbuti) > 0 (1.4 < *Z* < 5.8; Fig. 4C). These patterns indicate that on the southern and southwestern Tibetan Plateau, admixture with populations from other regions of the plateau resulted in ancestry fluctuation from 1100 to 700 B.P., but the influence was not sustained into present-day populations from this region. Present-day Tibetan populations also harbor a high proportion of ancestry related to lowland East Asians, as in a proximal qpAdm model, the ancestry of most populations on the eastern Tibetan Plateau can be accounted for by a source related to a 1900-year-old lowland population from the Upper Yellow River (Dacaozi1.9k; Fig. 4, D and E and table S17). These patterns are not found in 1100- to 700-year-old individuals, suggesting extensive gene flow from lowland East Asians occurred over the past 700 years. Present-day populations on the Tibetan Plateau are characterized by a longitudinal cline with eastern populations showing higher genetic affinity with lowland East Asians (fig. S26) (*22*). However, this pattern is not observed in different time transects of 3000- to 700-year-old plateau populations (Supplementary Text and figs. S27 to S30), which suggests that the longitudinal cline occurred recently, possibly associated with extensive recent gene flow from lowland East Asia.

### Genetic shifts and persistence in the central and southeastern Tibetan Plateau

The central plateau in the Nagqu prefecture is known for its harsh living conditions, with an average elevation of over 4500 masl (Fig. 1B). Ancient individuals before 2500 B.P. in this region form a southeast-central cluster with populations from the Chamdo prefecture of the southeastern Tibetan Plateau (Fig. 2, A and B). qpWave analysis shows that their southeastern plateau ancestry was distinct from the southern plateau ancestry found in regions along the Yarlung Tsangpo River (*P* < 0.0024, chi-square test; table S16). After 1600 B.P., however, all ancient individuals from the Nagqu prefecture, including Nagqu1.6k (*n* = 1), Nagqu1.4k (*n* = 3), and Nagqu1.1k (*n* = 1), share the most genetic drift with ancient individuals from the south-southwest cluster who mainly carries southern plateau ancestry (fig. S23). In an *f*_4_ analysis, the 1600- to 1400-year-old individuals from the Nagqu prefecture share more alleles with ancient individuals from the south-southwest cluster than those from the southeast-central cluster, i.e., *f*_4_ (3000 to 2000 B.P. southern/southwestern Tibetan populations, 2700 to 2500 B.P. Nagqu; <1600 B.P. Nagqu, Mbuti) > 0 (−0.4 < *Z* < 6.7, fig. S20). In phylogenetic and *f*_4_ analyses, these individuals fall entirely within the diversity of Early Ancient Tibetans from the south-southwest cluster, rather than grouping with the southeast-central cluster (b.s. = 1000/1000; Fig. 2A and table S13). In a proximal qpAdm analysis, the 1600- to 1400-year-old individuals from Nagqu are best described as sharing ancestry with Early Ancient Tibetans of the south-southwest cluster (Fig. 4D and table S17), while the 2500-year-old individuals from Nagqu are best described as sharing ancestry with either the southeast-central or northeast cluster (table S17). Notably, the 1600-year-old Nagqu individuals are more related to the 1900-year-old individuals from the West Shigatse prefecture on the southwestern plateau, whereas the 1400- to 1100-year-old individuals from Nagqu are more related to the 2200- to 2100-year-old individuals from the East Shigatse and Shannan prefectures on the southern plateau (Fig. 3A and table S19). These observations suggest that between 2500 and 1600 years ago, there was a pronounced population shift on the central plateau, such that populations by 1600 years ago primarily have ancestry related to the south-southwest cluster, and subtle genetic changes were further observed between 1600-year-old and 1400- to 1100-year-old individuals from the Nagqu prefecture.

With the widespread genetic influence of southern plateau ancestry on the Tibetan Plateau, we next examined the extent to which southeastern plateau ancestry persisted into the present day. In the Nyingchi prefecture of the southeastern Tibetan Plateau, an 800-year-old individual from the Kangyu archaeological site harbors ancestry related to the southeast-central cluster (Fig. 4D and table S17), a pattern that differs from the 2000-year-old Nyingchi individuals who instead harbor southern plateau ancestry. On the northeastern Tibetan Plateau, a 500-year-old individual from Yushu also harbors southeastern plateau ancestry (Fig. 4D and table S17). These results show that southeastern plateau ancestry may have spread into the Nyingchi and Yushu prefectures by at least 800 and 500 years ago, respectively. Moreover, the southeastern plateau ancestry can still be found today in the southeastern Tibetan Plateau. The present-day Naxi population was modeled as a mixture of ~39% southeastern plateau ancestry and ~61% ancestry related to lowland East Asians (Fig. 4E and table S17). Our analyses show that the southeastern plateau ancestry persists in partial amounts across the eastern half of the Tibetan Plateau into the present day.

### Genetic heterogeneity associated with Central Asian ancestry on the Tibetan Plateau

Several outlier sites and individuals reveal that Central Asian ancestry played a role in many different regions of the plateau, adding genetic heterogeneity to the plateau population. On the western Tibetan Plateau, 2300-year-old individuals from the Piyangjiweng site mainly harbor southern plateau ancestry, but they also share ancestry with Bronze Age individuals from the Gonur archaeological site in Central Asia (Fig. 4A) (*30*). In an *f*_4_ analysis, ancient Bronze Age populations from Iran and Turkmenistan share more alleles with the 2300-year-old individuals from the Ngari prefecture (Piyangjiweng) than with other ancient plateau individuals, i.e., *f*_4_ (Early Ancient Tibetans, Ngari2.3k; Bronze Age Central Asians, Mbuti) < 0 (−5.7 < *Z* < −1.1; fig. S25 and table S9D). In ADMIXTURE and admixture *f*_3_ analyses, the Piyangjiweng individuals can be described as a mixture of ancestry related to ancient plateau populations and Bronze Age Central Asians (Fig. 1C and table S10), and in a PCA, they are positioned closer to the Central Asian cline (Fig. 1E). In a qpAdm analysis, mixture models estimate that the 2300-year-old Ngari individuals have ~6 to 14% Central Asian–related ancestry (Fig. 4A and table S17). Furthermore, an individual who dates to 262 and 27 B.P. from the Gelintang archaeological site in the Ngari prefecture also harbors Central Asian–related ancestry (14%) and in greater excess than the 2300-year-old individuals (fig. S25 and table S9D). These patterns show that Central Asian–related ancestry affected the western Tibetan Plateau as early as 2300 years ago and has been sustained until recent historical times.

Central Asian–related influences can also be observed in the southern and southwestern Tibetan Plateau. An individual who dates to 1520 to 1363 B.P. (Shigatse1.5k_1) from the Zhangcun archaeological site shows distinct genetic patterns than other 2200- to 700-year-old individuals sampled from this region. In a PCA, this individual is shifted toward the Indian population cline (Fig. 1F), and in an ADMIXTURE analysis, he harbors a component maximized in Neolithic Iranian farmers (Fig. 1C), which suggests that Shigatse1.5k_1 has Central Asian–related ancestry. These patterns are not observed in another individual from the same site (Shigatse1.5k_2), whose ancestry can be fully accounted for with sources related to the plateau population (Fig. 4D and table S17). In addition, 900-year-old individuals from the Longsangquduo archaeological site on the southern Tibetan Plateau shares more alleles with Bronze Age individuals from Central Asia (*30*) (i.e., Shahr_I_Sokhta_BA1 and Gonur1_BA) than other nearby contemporary plateau individuals, i.e., *f*_4_ (Shigatse0.9k, Lhasa1k/Lhasa0.7k/Shigatse0.7k; Ancient Central Asians, Mbuti) > 0 (2.2 < *Z* < 4.3; fig. S25). These observations suggest that they also carry ancestry found in ancient Central Asians. Today, the Sherpa from the southwestern Tibetan Plateau harbors more Central Asian–related ancestry (black component) than present-day Tibetans (fig. S14), and in *f*_4_ analyses, they also tend to share more alleles with Bronze Age Central Asians, i.e., *f*_4_ (Sherpa_Shigatse, present-day Tibetans; Shahr_I_Sokhta_BA1/Gonur1_BA, Mbuti) > 0 (1.1 < *Z* < 3.9; fig. S25). These patterns show that populations carrying Central Asian–related ancestry have also contributed to humans in the southern and southwestern regions as early as 1500 B.P.

### Haplotype frequencies of *EPAS1*

Present-day Tibetans harbor a unique haplotype of the endothelial Pas domain protein 1 gene (*EPAS1*) that introgressed into modern humans from archaic humans known as Denisovans (*31*–*33*). The unique haplotype shows a strong signature of positive selection in present day Tibetans and has likely facilitated their adaptation to high altitudes. However, it remains elusive what role positive selection and demographic shifts have played in shaping the landscape of *EPAS1* haplotype frequencies in human populations. We tracked the trajectory of the adaptive *EPAS1* variant found in present-day Tibetans over time by examining its presence in the 5100- to 700-year-old individuals on the Tibetan Plateau. The ~5100-year-old Zongri individual from the northeastern plateau harbors two copies of the adaptive haplotype, which indicates that the oldest known modern human on the Tibetan Plateau is homozygous for the *EPAS1* adaptive haplotype (table S21). The adaptive haplotype is detected in 11 of the 17 Zongri individuals (4800 to 3900 years old), with each having one copy of the adaptive haplotype (table S21), showing that the adaptive haplotype was at fairly high frequency during the early human occupation of the northeastern plateau.

**Fig. 5.**
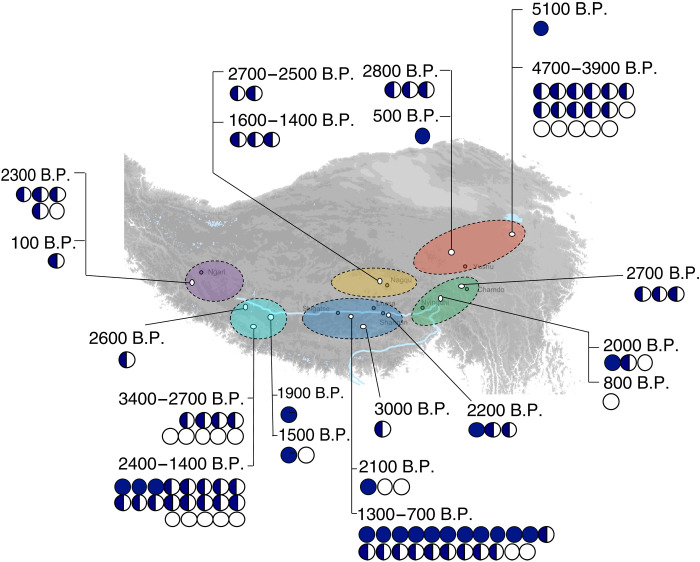
The geographic and temporal distribution of *EPAS1* alleles. Each circle represents an *EPAS1* genotype in one ancient Tibetan individual. White circles represent individuals who are homozygous for the nonadaptive *EPAS1* haplotype, blue circles represent individuals who are homozygous for the adaptive *EPAS1* haplotype, and half-filled circles represent heterozygous individuals. Colored regions on the map correspond to the same colors on the map in Fig. 1. The haplotype was called on the basis of information at 20 SNPs for individuals with at least four reads available at each position.

In the vast region encompassing the central, southeastern, southern, and southwestern plateau, one copy of the adaptive haplotype is detected in 11 of 16 individuals dated to 3500 to 2500 B.P. (Fig. 5). The estimated proportion of the adaptive haplotype is 0.36 [95% confidence interval (CI), 0.21 to 0.52; *n* = 19] for the ancient plateau individuals older than 2500 B.P., 0.47 (95% CI, 0.33 to 0.61; *n* = 24) for the 2400- to 1900-year-old plateau individuals, and 0.59 (95% CI, 0.47 to 0.71; *n* = 37) for the more recent 1600- to 700-year-old plateau individuals, showing an increasing trend from 3000 to 700 B.P. Nevertheless, all of these estimated proportions are much lower than that observed in present-day Tibetans (0.86; *n* = 33) from the same vast region (table S22) marking a substantial increase in the *EPAS1* haplotype frequency over the past 3000 years and a sharp increase over the past 700 years. This pattern shows no relationship to population shifts on the Tibetan Plateau, where despite local shifts within the plateau and influences from nonplateau regions, there is high genetic continuity into the present day. In comparison to randomly chosen SNPs across the genome, the haplotype frequency increased significantly (*P* = 0.0055; figs. S31 and S32), suggesting that it was driven by positive selection on this locus rather than demographic events.

## DISCUSSION

In this study, we show that the unique ancestry in present-day plateau populations can be found in ancient individuals across the entire Tibetan Plateau, extending as far back as 5100 B.P. We found that this plateau ancestry was formed through a mixture of a minor source from a deeply diverged population and a major source related to ancient northern East Asians, consistent with previous observations in 3400- to 1200-year-old individuals from the Himalayan arc in Nepal. The minor source is attributable to a ghost population that is deeply diverged from available Asian lineages. Whether this deeply diverged source is related to Paleolithic populations on Tibetan Plateau remains to be determined by future ancient DNA studies. We also note that this must have occurred before 5100 B.P., as the oldest individual sampled to date from the plateau similarly shows this unique ancestral pattern. The major source contributing to the plateau ancestry is related to diverse 9500- to 4000-year-old populations from northern East Asia spanning the Yellow River region and Inner Mongolia. This period overlaps with the Early to Middle Neolithic in northern China, which was characterized by substantial human population growth (*34*, *35*). When the formation of the mixed ancestry observed in all ancient and present-day plateau populations occurred is not yet known, but it seems related to human population movement and expansion in northern East Asia.

We documented the earliest instance of plateau ancestry sampled to date from a 5100-year-old individual at the Zongri archaeological site in the Gonghe Basin on the northeastern Tibetan Plateau. An examination of genetic patterns in 5100- to 3900-year-old individuals at the site suggests that human populations at Zongri experienced genetic admixture with ancient northern East Asians from the Yellow River region by 4700 B.P. (*7*, *36*), a pattern not observed in the neighboring higher elevation Pukagongma site in the Yushu prefecture. The northern East Asian ancestry related to the admixture at this period was likely associated with millet farmers who expanded into the Upper Yellow River valley around the eastern margins of the Tibetan Plateau around 6000 to 4000 years ago (*24*, *37*). The earliest residents of the Zongri site, dating to 5500 to 4000 years ago, were shown to be primarily foragers who likely traded with millet farmers from the upper Yellow River valley (*11*, *24*, *25*). Our findings show that the cultural interaction on the eastern margins of the Tibetan Plateau was coupled with genetic interactions as early as 4700 B.P. At least on the eastern margins of the Tibetan Plateau, cultural and genetic exchanges were both fundamental to shaping human interactions in this region.

Although all Early Ancient Tibetans share a similar genetic makeup, i.e., mixed Tibetan ancestry, the differentiation between 5100- to 2500-year-old individuals across the Tibetan Plateau is deep enough that three main genetic clusters can be identified: a northeast cluster spanning the Yushu prefecture and Gonghe Basin in Qinghai (northeastern plateau ancestry), a southeast-central cluster spanning the Nagqu and Chamdo prefectures of Tibet Autonomous Region (southeastern plateau ancestry), and a south-southwest cluster spanning the Shigatse, Shannan, and Lhasa prefectures of Tibet Autonomous Region and the Himalayan arc of Nepal (southern plateau ancestry). This pattern shows that Tibetan ancestry is highly structured, with enough genetic differentiation to distinguish between ancient plateau individuals ranging from 5100 to 2500 B.P. This suggests distinct histories associated with each of these different clusters. Notably, the southern plateau ancestry has a surprisingly large geographic span along the Yarlung Tsangpo River, suggesting that the Yarlung Tsangpo River valley was an important corridor for prehistoric human migration on the Tibetan Plateau.

In the past three millennia, the Tibetan Plateau was home to several powerful polities. Archaeological and historical studies indicate that an early complex society called the Zhangzhung likely formed around 2500 B.P. in what is now the Ngari prefecture (*11*, *38*), followed by the Tibetan Empire (*39*) around 1300 B.P. that originated from the Shannan prefecture (*29*). We found that in the Nagqu prefecture in the central Tibetan Plateau, there was an expansion of southern plateau ancestry replacing the earlier southeastern plateau ancestry. The replacement occurred before 1600 B.P., predating the conquest of the Nagqu region by the Tibetan Empire (*29*). We found that the 1600-year-old Nagqu individual (Nagqu1.6k) showed the closest relationship with the 1900-year-old individual from Sding Chung (*40*) (Shigatse1.9k), a site nearby the putative capital of Zhangzhung (*41*), but more genetic data are needed to clarify population shifts in Nagqu related to the Zhangzhung. We also observed that the 1400- to 1100-year-old individuals from the Nagqu prefecture show a closer genetic relationship to the 2200- to 2100-year-old individuals from the Shannan prefecture where the Tibetan Empire arose. This correlates with the conquest of the central plateau by the Tibetan Empire in ~1300 B.P. (*29*), suggesting that the expansion of the Tibetan Empire also left a genetic legacy on populations in the Nagqu prefecture. In the southern plateau, local ancestry shifts can also be observed around 1100 B.P., a period coinciding with the collapse of the Tibetan Empire (*29*). The genetic patterns between 2500 and 1100 B.P. show high population interaction and diversity in the central plateau, at a time known to be historically impactful on the plateau. Capturing southern plateau ancestry in the central plateau region, when it was not observed in central plateau populations before 2500 B.P., shows a dynamism in this time period associated with local population shifts, increased population heterogeneity, and/or increased population connectivity and migration. Resolving which of these three explanations are more representative is difficult without further sampling in this region. More dense sampling from the central plateau is needed to fully reconstruct the regional population history during this intriguing period. Cross-regional interactions can also be observed in the southeastern and northeastern plateau, with southeastern plateau ancestry persisting in partial amounts in the Nyingchi and Yushu prefectures in the past millennia. These patterns suggest that these state-level societies have played an important role in facilitating gene flow between populations from different regions on the Tibetan Plateau. Present-day Tibetans and Sherpa reflect these historical shifts, with nearly all present-day Tibetans showing some southern plateau ancestry making them share a high genetic similarity, although a record of partial amounts of southeastern plateau ancestry remains in some present-day populations from the eastern half of the plateau.

While the predominant ancestry from 5100 B.P. to the present day is a shared plateau ancestry, several outliers from the different regions of the plateau show that there were interactions on the plateau with an array of populations influenced by a diverse set of ancestries across Asia. In the Chamdo and Nyingchi prefecture on the southeastern plateau, 2800- to 2000-year-old individuals show that connections to ancestry are also found in ancient individuals from southern East Asia. In the Shigatse prefecture of the southwestern plateau, 1500-year-old and 900-year-old individuals both show connections to ancient individuals from Central Asia, similar to the present-day Sherpa. In the Ngari prefecture of the western Tibetan Plateau, similar patterns are present across all sampled individuals, suggesting that Central Asian–related influences had a larger impact in this region (*42*). Heavier influence from lowland East Asia onto the plateau can be observed in present-day Tibetans, suggesting that the west-to-east population genetic cline in present-day Tibetan populations shifting toward lowland Han populations (*22*) is primarily a product of very recent human migration. Collectively, these patterns highlight the dynamic interactions that plateau populations likely had with their neighbors in low-elevation regions near the plateau.

We also found that the adaptive *EPAS1* haplotype associated with Denisovans (*33*) is found at a relatively high frequency in ancient plateau populations, but the highest frequencies are observed in present-day Tibetan populations—a pattern that is also observed previously (*9*). With the dense temporal sampling in our study, we observed an increased frequency of the adaptive haplotype over time, showing a steady increase in this haplotype over the past 3000 years and a sharp increase in the past 700 years. Notably, the oldest individual sampled to date on the plateau, the 5100-year-old Zongri individual, shows two copies of the adaptive haplotype, which suggests that this adaptive haplotype has a fairly long evolutionary history in ancient plateau populations associated with the unique haplotype. While we do not yet know how the adaptive haplotype first entered ancient plateau populations, our finding that the 5100-year-old Zongri individual was homozygous for the adaptive haplotype suggests its origins date back to the ancestral population that contributed to all plateau populations presently sampled.

Through sampling of modern humans across a wide temporal and spatial range on the Tibetan Plateau, we emphasize a genetic history unique to the Tibetan Plateau dating back to at least 5100 B.P. in the northeastern plateau and 3400 B.P. in the central and southern plateau, with strong regional differentiation until at least 2500 B.P. Interactions with diverse ancestries from neighboring regions affected plateau populations, but the largest genetic shifts are caused by the mixture of populations from different regions of the plateau, potentially associated with large-scale political shifts related to the expansion and collapse of major state-level societies in historical times. Additional sequencing of ancient genomes on and near the plateau is still needed to determine when the ancestors of these plateau populations first diverged from populations carrying other northern East Asian ancestries, as well as the source of the unknown ancestry found in all plateau populations. However, by sampling multiple humans from an additional 1700 years further back in time and expanding the range of available ancient human genomes to the entire Tibetan Plateau, we have characterized a dynamic population history marked by population movement and interaction both across populations within the plateau and with populations in neighboring regions.

## MATERIALS AND METHODS

### Ethics statement

Permission to sampled ancient DNA from the associated human specimens was granted by the provincial archaeological institutes or universities that manage and care for the specimens. In addition, the Institutional Review Board (202112020010) at the Institute of Vertebrate Paleontology and Paleoanthropology of the Chinese Academy of Sciences provided approval and oversight, allowing us to sample the genomes of the ancient humans included in this study.

### Geography nomenclatures

The sequenced ancient individuals from this study span across the Tibet Autonomous Region of China (Xizang), covering all seven prefecture-level divisions, i.e., Ngari, Shigatse, Lhasa, Shannan, Nagqu, Nyingchi, and Chamdo (fig. S1). Samples were also obtained from the Yushu and Hainan prefectures in the Qinghai province of China (fig. S1), which are both considered part of the Tibetan Plateau geographically and are mainly inhabited by Tibetans today. Throughout the study, we refer to the Tibetan Plateau by six subregions based on geographic locations (Fig. 1A and fig. S1). These include the northeastern Tibetan Plateau (Yushu and Hainan prefectures of Qinghai), central Tibetan Plateau (Nagqu), southeastern Tibetan Plateau (Chamdo and Nyingchi), southern Tibetan Plateau (Lhasa, Shannan, and eastern Shigatse), western Tibetan Plateau (Ngari), and the southwestern Tibetan Plateau (western Shigatse and the Himalayan arc region in Nepal). The Himalayan arc refers specifically to the Himalayan valley region of Nepal in the southwestern plateau.

### Ancient DNA extraction and library preparation

We prepared bone powder from 97 human samples collected across the Tibetan Plateau using a Dremel tool and single-use drill bits. We preferentially sampled from the inner ear region of the petrous bone when available and, otherwise, selected the best-preserved bone fragments (table S2). All the samples were prepared in a dedicated ancient DNA laboratory at the Institute of Vertebrate Paleontology and Paleoanthropology, Chinese Academy of Sciences, in Beijing, China. We extracted ancient DNA using an optimized protocol (*43*). DNA libraries were prepared using either double-stranded (DS) or single-stranded protocols (table S3) (*44*–*47*). We generated 113 libraries, where for 14 samples, we prepared multiple libraries. Sixty-four libraries were prepared with partial uracil–DNA–glycosylase treatment (“DS partial UDG”; table S3), which retains ancient DNA damage only at the terminal 3′ nucleotide (*44*).

### In solution capture of human mitochondrial and nuclear DNA

We captured both mitochondrial genomes (mtDNA) and genome-wide nuclear DNA by hybridizing the libraries with oligonucleotide probes (Agilent Technologies, California, USA). The mtDNA probes were developed to capture the complete mitochondrial genome (*48*), and the nuclear DNA probes were developed to enrich 1.2 million SNPs [the “1240k” dataset as described in (*49*)].

### Sequencing and reads alignment

All mtDNA libraries were sequenced using the Illumina MiSeq and HiSeq 2500 sequencing machines to generate 2 × 76–base pair (bp) paired-end reads, and all nuclear DNA libraries were sequenced using the Illumina HiSeq 2500 and HiSeq X sequencing machine to generate 2 × 100– and 2 × 150–bp paired-end reads. We trimmed adaptors and merged forward and reverse sequences into a single sequence using leeHom (https://github.com/grenaud/leeHom) (*50*), requiring a minimum overlap of 11 bp. We aligned these sequences to the human reference genome [revised Cambridge Reference Sequence 53 (rCRS53) for mtDNA and hg19 for nuclear) using the bwa (*51*) and samse commands (version 0.6.1), with the arguments “-n 0.01 -l 16500”. We excluded fragments with mapping quality less than 30. Reads with the same orientation, start, and end positions were considered duplicates, and we retained the read fragments with the highest average sequence quality for each set of duplicates.

### Tests of contamination and pseudo-haploid genotyping

First, the rates of C → T substitution at the terminal nucleotides were calculated for each individual to confirm the authenticity of the ancient DNA (table S3). Second, we used ContamMix (*52*) to estimate contamination levels by calculating the percentage of mtDNA reads that match the mtDNA consensus better than any of 311 mtDNA sequences representing a diverse set of present-day humans (*48*). Both ends (5 bp) of sequencing reads were masked from the calculation. DNA libraries with too few data to estimate contamination level or with estimated mtDNA contamination levels greater than 3.5% were not used in downstream population genetic analysis. Third, contamination of DNA libraries from male individuals were further estimated using ANGSD (*53*) based on the assumption that males are haploid for the X chromosome and, thus, should not be polymorphic at regions that do not combine with the Y chromosome. Individuals with estimated X-chromosome contamination greater than 3.5% were removed. Multiple libraries of the same individual were merged before calling pseudo-haploid genotypes, where one fragment per position was randomly sampled to determine the corresponding allele in that individual. Both ends (5 bp) of the sequencing reads were masked when determining pseudo-haploid genotypes.

### Radiocarbon dating

The same bone materials used for ancient DNA (aDNA) extraction were used for C14 dating. The dates were calibrated with OxCal v.4.4 using the IntCal20 (*54*) curve and presented as 95.4% CIs in calibrated years before the present, with present defined as 1950 CE.

### Sex determination

The sex of each individual was genetically determined by comparing the amount of sequence data that map to the Y chromosome to the amount of sequence data that map to the autosomes or the X chromosome (*55*). Only reads with mapping quality greater than or equal to 37 were considered.

### Uniparental haplogroups

The mitochondrial haplotypes were determined for each individual based on BAM files mapped to the rCRS (*56*). Only reads with a length of ≥30 bp and a mapping quality of ≥30 were considered. The mitogenomes were constructed with the consensus sequences at each site. We called haplogroups for each individual using HaploGrep2 (*57*) based on PhyloTree Build 17 (*56*). For the male samples, we determined Y-chromosome haplogroups by identifying the most derived allele upstream and the most ancestral allele downstream in the phylogenetic tree based on the International Society of Genetic Genealogy dataset version 10.01 (www.isogg.org/tree). If the most derived Y-chromosome SNP upstream was a C → T or G → A substitution, a substitution susceptible to ancient DNA damage, then we required at least two derived SNPs to assign the Y-chromosome haplogroup. Otherwise, we assigned the individual to the upstream haplogroup. When a more detailed haplogroup assignment could not be determined, the individual was assigned to the ancestral haplogroup they best fit [e.g., K (xLT), HIJK, and NO].

### Kinship analysis

The degree of kinship among different ancient DNA samples was estimated using lcMLkin (*12*) (https://github.com/COMBINE-lab/maximum-likelihood-relatedness-estimation), a tool that infers kinship using genotype likelihoods. The method takes into account the uncertainty in genotype calling when sequence coverage is low, which is common for ancient DNA samples. lcMLkin estimates the probability a pair of individuals are identical by descent (IBD), and it outputs the estimated probability that two diploid individuals share zero (k ^_0), one (k ^_1) or two (k ^_2) alleles at a given IBD loci, using this to determine the kinship coefficient (π ^). The expected values for k0, k1, and k2 depend on the familial relationship [see table 1 of (*58*)], such that estimating these values will allow inference of the kinship between these two individuals. For a reliable estimate, the method requires an SNP set with minor allele frequency higher than 5% and that the SNPs are independent of each other. To satisfy these conditions, we took all present-day East Asians from the Simons Genome Diversity Panel (SGDP) dataset (*59*), and in the 1.2 M SNP panel, we filtered for SNPs with an allele frequency higher than 5%. We then pruned the SNPs using PLINK (*60*), with the condition “--indep-pairwise 200 25 0.5,” which resulted in 217,266 SNPs available for subsequent kinship analysis. We called genotype likelihoods at these SNP sites for the ancient individuals using SNPbam2vcf.py (provided with lcMLkin) and estimated their biological relatedness using lcMLkin (table S4). We merged data of two specimens from the same burial when they were identified as deriving from the same individual. For samples with a shared familial relationship, we only retained genome-wide data for the individual in the kin group who had higher coverage for further downstream population genetic analysis.

### Dataset compilation

We collected previously published ancient and present-day human datasets to combine with the data generated in this study for the 1240k SNP panel. The present-day human dataset includes a worldwide set of present-day humans from the Simons Genome Diversity Panel (SGDP) (*59*) and present-day Tibetan, Sherpa, and Han (*6*, *14*, *59*, *61*). We also included ancient individuals from across Eurasia (*7*–*9*, *30*, *36*, *62*, *63*) and the Americas (*64*). In particular, we obtained genome-wide data for ancient individuals from the Himalayan arc region in Nepal (*8*, *9*) and merged them with the ancient individuals sampled in this study. A larger set containing more present-day humans from across East Asia and Nepal (*7*) who were genotyped using the Human Origin SNP panel (597,573 SNPs) were also included and used for the principal components analysis (PCA). Two genetic groups close to the northeast Tibetan Plateau, i.e., Upper_YR_LN and Upper_YR_IA from Ning *et al.* (*65*), are highly relevant to this study. To be specific, six individuals from the Lajia site in the Upper_YR_LN were included and named as Lajia4k, and all individuals from Upper_YR_IA were included and named Dacaozi1.9k throughout the manuscript.

### Principal components analysis

We used the smartpca program (version16000) in EIGENSOFT (*20*) (version 7.2.1) to perform the PCA, with parameters numoutlierevec: 2, outliersigmathresh: 12, lsqproject: YES, and autoshrink: YES. Present-day humans on or near the Tibetan Plateau genotyped in the Human Origins dataset (597,573 SNPs) were used to construct the principal component space, upon which we projected all ancient individuals. As choice of reference population can bias this analysis, we primarily used the PCA to examine the ancient individuals in the context of all present-day Asian populations.

### Unsupervised structure analysis with ADMIXTURE

The unsupervised structure analysis was performed using ADMIXTURE (version 1.3.0) (*26*) on autosomal SNPs of the 1240k dataset. In total, 1205 present-day samples and 555 ancient DNA samples were included. We removed SNPs at the CpG sites to avoid potential errors specific to ancient DNA data. We also removed SNPs that have missing data in more than 20% of the individuals and SNPs with minor allele frequencies lower than 0.5% (--geno 0.2 --maf 0.005). We pruned the dataset using the parameter --indep-pairwise 50 10 0.1, retaining 109,940 SNPs for the analysis. To identify *K* values with low cross-validation (CV) errors, we ran ADMIXTURE with *K* varying from 2 to 14 (fig. S14), and for each *K*, we ran 10 replicates using random seeds (table S5). The replicate of each *K* with the lowest CV error was plotted.

### *F*_ST_

*F*_ST_ analyses were performed using smartpca (version16000) from the EIGENSOFT package (*20*) (version 7.2.1) with the “fstonly: YES” option. For the pseudo-haploid data of ancient individuals, the “inbreed: YES” option was also added.

### *F* statistics

The software qp3Pop (v412) from the ADMIXTOOLS package (*14*) was used to calculate *f*_3_ statistics in the form of *f*_3_ (S1, S2; Target). The parameter inbreed: YES was used when the target populations were represented by pseudo-haploid ancient DNA. The software qpDstat (v712) was used to calculate the *f*_4_ statistics by turning on the “f4mode: YES” option. Autosomal SNPs from the 1240k dataset were used unless mentioned otherwise.

### Genetic clustering of ancient Tibetan Plateau samples

Individuals from the same archaeological sites were clustered into the same genetic set except when ancestral heterogeneity was observed. Ancestral heterogeneity was tested using *f*_4_ statistics in the form of *f*_4_ (X1, X2; *Z*, Mbuti) where X1 and X2 are individuals from the same test site and *Z* are individuals from a reference panel. The reference panel consists of populations that are temporally or geographically relevant to the studied ancient Tibetan Plateau individuals, including Tianyuan, UstIshim, Onge, Gonur1_BA, Afanasievo, Shamanka_EN, AR19K, Yumin, Boshan, YR_MN, Lajia4k, Qihe3, and Atayal. To increase the power, we also added five representative, high-coverage individuals across the Tibetan Plateau to the panel, including C4783_C202 from Zongri, C1 from Chokhopani, C3430 from Luozhating, CSP142 from Redilong, and CSP136 from Pukagongma. Individuals causing rejection of *f*_4_ ~ 0 were isolated and assigned to a different genetic group. For the genetic groups established in this study, we assigned names in the format of “region + age.” We used the prefecture names as the “region” names for individuals except at the Zongri site, where we keep the “Zongri” notation. The “age” used in the notation is averaged from the calibrated ages of the carbon dates of all individuals in the genetic group and then rounded to the nearest hundred.

### qpAdm

We used qpAdm (v1000) program from the ADMIXTOOLS v.6.0 package (*14*) to model population ancestries. The “allsnps: YES” option was used so that every individual *f*_4_ statistic was calculated using the intersection of the four populations involved, maximizing the power of the tests without introducing bias (*66*). The autosomal SNPs of the 1240k dataset were used in the analysis, and the CpG sites were masked when testing present-day populations as target populations. Mixture models with one-, two-, and three-way mixtures were successively tested until feasible models are found.

A “rotating scheme” (*17*) was adopted for investigating ancestry in ancient plateau populations older than 2500 B.P. (Early Ancient Tibetans). In a rotating scheme, outgroups are rotated in as potential source populations, allowing up to three sources at a time. For distal models, 15 populations (“R15”) were included in the reference set representing diverse ancestries, particularly from East Asia: Mota, UstIshim, Tianyuan, Malta1, Clovis, CHG, Ganj_Dareh_N, Gonur1_BA, Shamanka_EN, Yumin, Shandong_EN, Fujian_EN, YR_MN, Atayal, and Onge. For proximal models, we additionally added Zongri5.1k to form the “R15 + Zongri5.1k” rotating outgroup set for later Zongri groups. We tested both “R15 + Lajia4k + Zongri5.1k” and “R15 + Lajia4k + Lubrak” rotating outgroup sets for Early Ancient Tibetans younger than 3000 B.P.

We then proceeded to assess mixture models for ancient plateau populations younger than 2800 B.P. With younger populations, we used a proximal model allowing up to five possible sources: Chamdo2.8k_1, Yushu2.8k, and Lubrak representing different plateau ancestries; Gonur1_BA and Dacaozi1.9k representing lowland sources from South/Central and East Asia. Ten populations (“O10”) including Mota, UstIshim, Tianyuan, Clovis, Ganj_Dareh_N, Yumin, Tanshishan, Lajia4k, Suila, and Zongri5.1k were used as the right population in qpAdm analysis. In a qpWave analysis, we found that this outgroup set could distinguish ancestries among the five source populations, as an f4rank of four was rejected at *P* = 0.015. More details can be found on the “qpAdm modeling” section of Supplementary Text.

### qpGraph

We used qpGraph from the ADMIXTOOLS package (*14*) to model the relationship between populations using the allsnps: YES option, and CpG sites were excluded from the analysis.

### TreeMix

TreeMix (version 1.13) (*15*) was used to infer the maximum likelihood admixture graphs based on population allele frequency. The trees were rooted with the Mbuti population, and we set each block to contain 1000 SNPs with the option “-k 1000”. The bootstrap values for each clade in the tree were obtained by running 1000 replicates with the options “-bootstrap –q.”

### Archaic ancestry inference with admixfrog

We used the Admixfrog software (*67*) (version 0.5.6, https://github.com/BenjaminPeter/admixfrog/) to infer the introgressed tracts from archaic sources in our 57 ancient samples with more than 333,000 SNPs (table S20). We assumed that these target individuals has ancestry from the following three sources: Two archaic sources are represented by the high-coverage archaic genomes from Neandertals (NEA) (*68*, *69*) and Denisova 3 (DEN) (*44*), and a third potential source is represented by 44 genomes of present-day Sub-Saharan Africans from the SGDP (AFR) (*59*). Input files were generated using parameters “--length-bin-size 35 –minmapq 25 --deam-cutoff 3” to remove fragments with length less than 35 bp, or low mapping quality (less than 25), and to mask the deaminated bases in the first (C → T) or the last (G → A) three positions. Using the generated input files, admixfrog was run with the parameters “--states AFR NEA DEN --bin-size 5000 --ancestral PAN”, indicating the three sources we used (AFR, NEA, and DEN), a window size of 5000 bp, and the reference genome (PAN for Chimpanzee, panTro4, GCA_000001515.4) used to infer the ancestral state of each allele, respectively. Other parameters were set with default options (*67*).

### Phenotypic SNPs

For the *EPAS1* gene, 20 SNPs that are highly differentiated between Tibetans and other modern humans were used to define the *EPAS1* haplotype common to present-day Tibetan populations. We assumed that the 20 SNPs are in complete linkage and reads mapping to different SNP sites were independent. The probabilities of homozygosity for the common Tibetan haplotype, homozygosity for the haplotype found in most present-day humans, or heterozygosity found in most present-day humans were determined with a Bayesian framework with a flat prior using the aggregated number of mapped reads across the 20 SNPs highly differentiated between Tibetan and Han populations. The genotype posterior probability at each locus, accounting for both errors due to sequencing and due to ancient DNA damage (ε) is given by the following equation:

$$P\;(G=g|r,t)=\frac{P\;(r,t|g)}{\sum_{G}P\;(r,t|G)}$$

where ∑*_G_P* (*r*, *t* ∣ *G*) = *B*(*r*, *t*,1 − ε) + *B*(*r*, *t*, ε) + *B*(*r*, *t*, 0.5), *r* is the number of reads observed for the allele, and *t* is the total number of reads observed. The allele frequency (*p*) of *EPAS1* was estimated using a maximum likelihood framework where the total number of reads across all 20 SNPs was used to calculate the allele frequency:

$$l(p|r,t)=\sum_{i=1}^{N}\log[p^{2}B(r_{i},t_{i},1-\varepsilon )+2p(1-p)B(r_{i},t_{i},0.5)+{\left(1-p\right)}^{2}B(r_{i},t_{i},\varepsilon )]$$

where *r*_*i*_, *t*_*i*_, and ε are equivalent to that found above and *i* refers to the *i*th individual.

Individuals with contamination or low SNP counts were excluded from the analyses. For other functional SNPs, we similarly count the number of mapped reads at each SNP. We restricted the analyses to reads with a mapping quality of ≥30 and bases with a quality of ≥30.

## Supplementary Materials

### This PDF file includes:

Supplementary Text Figs. S1 to S33 Legends for tables S1 to S3, S8 to S10, S14, S15, S17, S20, S21, S23 Tables S4 to S7, S11 to S13, S16, S18, S19, S22 References

## References

[1] F. Chen, F. Welker, C. C. Shen, S. E. Bailey, I. Bergmann, S. Davis, H. Xia, H. Wang, R. Fischer, S. E. Freidline, T. L. Yu, M. M. Skinner, S. Stelzer, G. Dong, Q. Fu, G. Dong, J. Wang, D. Zhang, J. J. Hublin, A late middle Pleistocene Denisovan mandible from the Tibetan Plateau. Nature 569, 409–412 (2019). 31043746 10.1038/s41586-019-1139-x

[2] X. L. Zhang, B. B. Ha, S. J. Wang, Z. J. Chen, J. Y. Ge, H. Long, W. He, W. Da, X. M. Nian, M. J. Yi, X. Y. Zhou, P. Q. Zhang, Y. S. Jin, O. Bar-Yosef, J. W. Olsen, X. Gao, The earliest human occupation of the high-altitude Tibetan Plateau 40 thousand to 30 thousand years ago. Science 362, 1049–1051 (2018). 30498126 10.1126/science.aat8824

[3] F. H. Chen, G. H. Dong, D. J. Zhang, X. Y. Liu, X. Jia, C. B. An, M. M. Ma, Y. W. Xie, L. Barton, X. Y. Ren, Z. J. Zhao, X. H. Wu, M. K. Jones, Agriculture facilitated permanent human occupation of the Tibetan Plateau after 3600 B.P. Science 347, 248–250 (2015). 25593179 10.1126/science.1259172

[4] M. C. Meyer, M. S. Aldenderfer, Z. Wang, D. L. Hoffmann, J. A. Dahl, D. Degering, W. R. Haas, F. Schlutz, Permanent human occupation of the central Tibetan Plateau in the early Holocene. Science 355, 64–67 (2017). 28059763 10.1126/science.aag0357

[5] M. Aldenderfer, Peopling the Tibetan Plateau: Insights from archaeology. High Alt. Med. Biol. 12, 141–147 (2011). 21718162 10.1089/ham.2010.1094

[6] D. Lu, H. Lou, K. Yuan, X. Wang, Y. Wang, C. Zhang, Y. Lu, X. Yang, L. Deng, Y. Zhou, Q. Feng, Y. Hu, Q. Ding, Y. Yang, S. Li, L. Jin, Y. Guan, B. Su, L. Kang, S. Xu, Ancestral origins and genetic history of Tibetan highlanders. Am. J. Hum. Genet. 99, 580–594 (2016). 27569548 10.1016/j.ajhg.2016.07.002 PMC5011065

[7] C.-C. Wang, H.-Y. Yeh, A. N. Popov, H.-Q. Zhang, H. Matsumura, K. Sirak, O. Cheronet, A. Kovalev, N. Rohland, A. M. Kim, S. Mallick, R. Bernardos, D. Tumen, J. Zhao, Y.-C. Liu, J.-Y. Liu, M. Mah, K. Wang, Z. Zhang, N. Adamski, N. Broomandkhoshbacht, K. Callan, F. Candilio, K. S. D. Carlson, B. J. Culleton, L. Eccles, S. Freilich, D. Keating, A. M. Lawson, K. Mandl, M. Michel, J. Oppenheimer, K. T. Ozdogan, K. Stewardson, S. Wen, S. Yan, F. Zalzala, R. Chuang, C.-J. Huang, H. Looh, C.-C. Shiung, Y. G. Nikitin, A. V. Tabarev, A. A. Tishkin, S. Lin, Z.-Y. Sun, X.-M. Wu, T.-L. Yang, X. Hu, L. Chen, H. Du, J. Bayarsaikhan, E. Mijiddorj, D. Erdenebaatar, T.-O. Iderkhangai, E. Myagmar, H. Kanzawa-Kiriyama, M. Nishino, K.-I. Shinoda, O. A. Shubina, J. Guo, W. Cai, Q. Deng, L. Kang, D. Li, D. Li, R. Lin, R. S. Nini, L.-X. Wang, L. Wei, G. Xie, H. Yao, M. Zhang, G. He, X. Yang, R. Hu, M. Robbeets, S. Schiffels, D. J. Kennett, L. Jin, H. Li, J. Krause, R. Pinhasi, D. Reich, Genomic insights into the formation of human populations in East Asia. Nature 591, 413–419 (2021). 33618348 10.1038/s41586-021-03336-2 PMC7993749

[8] C. Jeong, A. T. Ozga, D. B. Witonsky, H. Malmstrom, H. Edlund, C. A. Hofman, R. W. Hagan, M. Jakobsson, C. M. Lewis, M. S. Aldenderfer, A. Di Rienzo, C. Warinner, Long-term genetic stability and a high-altitude east Asian origin for the peoples of the high valleys of the Himalayan arc. Proc. Natl. Acad. Sci. U.S.A. 113, 7485–7490 (2016). 27325755 10.1073/pnas.1520844113 PMC4941446

[9] C.-C. Liu, D. Witonsky, A. Gosling, J. H. Lee, H. Ringbauer, R. Hagan, N. Patel, R. Stahl, J. Novembre, M. Aldenderfer, C. Warinner, A. Di Rienzo, C. Jeong, Ancient genomes from the Himalayas illuminate the genetic history of Tibetans and their Tibeto-Burman speaking neighbors. Nat. Commun. 13, 1203 (2022). 35260549 10.1038/s41467-022-28827-2 PMC8904508

[10] R. Czai, *Tibetan Historical Manuscripts* (Gansu People Publishing Company, 2010).

[11] J. d’Alpoim Guedes, M. Aldenderfer, The archaeology of the early Tibetan Plateau: New research on the initial peopling through the early bronze age. J. Archaeol. Res. 28, 339–392 (2020).

[12] M. Lipatov, K. Sanjeev, R. Patro, K. Veeramah, Maximum likelihood estimation of biological relatedness from low coverage sequencing data. bioRxiv 10.1101/023374 [Preprint]. 29 July 2015. 10.1101/023374.

[13] A. L. Price, N. J. Patterson, R. M. Plenge, M. E. Weinblatt, N. A. Shadick, D. Reich, Principal components analysis corrects for stratification in genome-wide association studies. Nat. Genet. 38, 904–909 (2006). 16862161 10.1038/ng1847

[14] N. Patterson, P. Moorjani, Y. Luo, S. Mallick, N. Rohland, Y. Zhan, T. Genschoreck, T. Webster, D. Reich, Ancient admixture in human history. Genetics 192, 1065–1093 (2012). 22960212 10.1534/genetics.112.145037 PMC3522152

[15] J. K. Pickrell, J. K. Pritchard, Inference of population splits and mixtures from genome-wide allele frequency data. PLOS Genet. 8, e1002967 (2012). 23166502 10.1371/journal.pgen.1002967 PMC3499260

[16] W. Haak, I. Lazaridis, N. Patterson, N. Rohland, S. Mallick, B. Llamas, G. Brandt, S. Nordenfelt, E. Harney, K. Stewardson, Q. Fu, A. Mittnik, E. Banffy, C. Economou, M. Francken, S. Friederich, R. G. Pena, F. Hallgren, V. Khartanovich, A. Khokhlov, M. Kunst, P. Kuznetsov, H. Meller, O. Mochalov, V. Moiseyev, N. Nicklisch, S. L. Pichler, R. Risch, M. A. R. Guerra, C. Roth, A. Szecsenyi-Nagy, J. Wahl, M. Meyer, J. Krause, D. Brown, D. Anthony, A. Cooper, K. W. Alt, D. Reich, Massive migration from the steppe was a source for Indo-European languages in Europe. Nature 522, 207–211 (2015). 25731166 10.1038/nature14317 PMC5048219

[17] É. Harney, N. Patterson, D. Reich, J. Wakeley, Assessing the performance of qpAdm: A statistical tool for studying population admixture. Genetics 217, iyaa045 (2021). 33772284 10.1093/genetics/iyaa045 PMC8049561

[18] I. Lazaridis, A. Mittnik, N. Patterson, S. Mallick, N. Rohland, S. Pfrengle, A. Furtwangler, A. Peltzer, C. Posth, A. Vasilakis, P. J. P. McGeorge, E. Konsolaki-Yannopoulou, G. Korres, H. Martlew, M. Michalodimitrakis, M. Ozsait, N. Ozsait, A. Papathanasiou, M. Richards, S. A. Roodenberg, Y. Tzedakis, R. Arnott, D. M. Fernandes, J. R. Hughey, D. M. Lotakis, P. A. Navas, Y. Maniatis, J. A. Stamatoyannopoulos, K. Stewardson, P. Stockhammer, R. Pinhasi, D. Reich, J. Krause, G. Stamatoyannopoulos, Genetic origins of the Minoans and Mycenaeans. Nature 548, 214–218 (2017). 28783727 10.1038/nature23310 PMC5565772

[19] G. He, M. Wang, X. Zou, P. Chen, Z. Wang, Y. Liu, H. Yao, L.-H. Wei, R. Tang, C.-C. Wang, H.-Y. Yeh, Peopling history of the Tibetan Plateau and multiple waves of admixture of Tibetans inferred from both ancient and modern genome-wide data. Front. Genet. 12, 725243 (2021). 34650596 10.3389/fgene.2021.725243 PMC8506211

[20] N. Patterson, A. L. Price, D. Reich, Population structure and eigenanalysis. PLOS Genet. 2, e190 (2006). 17194218 10.1371/journal.pgen.0020190 PMC1713260

[21] M. Raghavan, P. Skoglund, K. E. Graf, M. Metspalu, A. Albrechtsen, I. Moltke, S. Rasmussen, T. W. Stafford Jr., L. Orlando, E. Metspalu, M. Karmin, K. Tambets, S. Rootsi, R. Magi, P. F. Campos, E. Balanovska, O. Balanovsky, E. Khusnutdinova, S. Litvinov, L. P. Osipova, S. A. Fedorova, M. I. Voevoda, M. DeGiorgio, T. Sicheritz-Ponten, S. Brunak, S. Demeshchenko, T. Kivisild, R. Villems, R. Nielsen, M. Jakobsson, E. Willerslev, Upper Palaeolithic Siberian genome reveals dual ancestry of native Americans. Nature 505, 87–91 (2014). 24256729 10.1038/nature12736 PMC4105016

[22] C. Jeong, B. M. Peter, B. Basnyat, M. Neupane, C. M. Beall, G. Childs, S. R. Craig, J. Novembre, A. Di Rienzo, A longitudinal cline characterizes the genetic structure of human populations in the Tibetan Plateau. PLOS ONE 12, e0175885 (2017). 28448508 10.1371/journal.pone.0175885 PMC5407838

[23] H. H. Chen, G. S. Wang, D. Z. Mei, N. Suo, Brief report on the excavation of Zongri site, Tongde County, Qinghai. Archaeology (Chinese), 1–14 (1998).

[24] L. L. Ren, G. H. Dong, F. W. Liu, J. d'Alpoim-Guedes, R. K. Flad, M. M. Ma, H. M. Li, Y. S. Yang, Y. J. Liu, D. J. Zhang, G. L. Li, J. Y. Li, F. H. Chen, Foraging and farming: Archaeobotanical and zooarchaeological evidence for Neolithic exchange on the Tibetan Plateau. Antiquity 94, 637–652 (2020).

[25] L. Y. Hung, J. F. Cui, H. H. Chen, Emergence of Neolithic communities on the northeastern Tibetan Plateau: Evidence from the Zongri cultural sites, in *BAR International Series 2679* (The ‘Crescent -Shaped Cultural Communication Belt’: Tong Enzheng’s Model in Retrospect: An Examination of Methodological, Theoretical and Material Concerns of Long-Distance Interactions in East Asia, Archaeopress, 2014), pp. 65–78.

[26] D. H. Alexander, J. Novembre, K. Lange, Fast model-based estimation of ancestry in unrelated individuals. Genome Res. 19, 1655–1664 (2009). 19648217 10.1101/gr.094052.109 PMC2752134

[27] A. M. Hudson, J. W. Olsen, J. Quade, G. Lei, T. E. Huth, H. Zhang, A regional record of expanded Holocene wetlands and prehistoric human occupation from paleowetland deposits of the western Yarlung Tsangpo valley, southern Tibetan Plateau. Quatern. Res. 86, 13–33 (2016).

[28] Z. Ling, X. Yang, Y. Wang, Y. Wang, J. Jin, D. Zhang, F. Chen, OSL chronology of the Liena archeological site in the Yarlung Tsangpo valley throws new light on human occupation of the Tibetan Plateau. The Holocene 30, 1043–1052 (2020).

[29] S. v. Schaik, *Tibet: A History* (Yale University Press, 2011), pp. 352.

[30] V. M. Narasimhan, N. Patterson, P. Moorjani, N. Rohland, R. Bernardos, S. Mallick, I. Lazaridis, N. Nakatsuka, I. Olalde, M. Lipson, A. M. Kim, L. M. Olivieri, A. Coppa, M. Vidale, J. Mallory, V. Moiseyev, E. Kitov, J. Monge, N. Adamski, N. Alex, N. Broomandkhoshbacht, F. Candilio, K. Callan, O. Cheronet, B. J. Culleton, M. Ferry, D. Fernandes, S. Freilich, B. Gamarra, D. Gaudio, M. Hajdinjak, E. Harney, T. K. Harper, D. Keating, A. M. Lawson, M. Mah, K. Mandl, M. Michel, M. Novak, J. Oppenheimer, N. Rai, K. Sirak, V. Slon, K. Stewardson, F. Zalzala, Z. Zhang, G. Akhatov, A. N. Bagashev, A. Bagnera, B. Baitanayev, J. Bendezu-Sarmiento, A. A. Bissembaev, G. L. Bonora, T. T. Chargynov, T. Chikisheva, P. K. Dashkovskiy, A. Derevianko, M. Dobes, K. Douka, N. Dubova, M. N. Duisengali, D. Enshin, A. Epimakhov, A. V. Fribus, D. Fuller, A. Goryachev, A. Gromov, S. P. Grushin, B. Hanks, M. Judd, E. Kazizov, A. Khokhlov, A. P. Krygin, E. Kupriyanova, P. Kuznetsov, D. Luiselli, F. Maksudov, A. M. Mamedov, T. B. Mamirov, C. Meiklejohn, D. C. Merrett, R. Micheli, O. Mochalov, S. Mustafokulov, A. Nayak, D. Pettener, R. Potts, D. Razhev, M. Rykun, S. Sarno, T. M. Savenkova, K. Sikhymbaeva, S. M. Slepchenko, O. A. Soltobaev, N. Stepanova, S. Svyatko, K. Tabaldiev, M. Teschler-Nicola, A. A. Tishkin, V. V. Tkachev, S. Vasilyev, P. Veleminsky, D. Voyakin, A. Yermolayeva, M. Zahir, V. S. Zubkov, A. Zubova, V. S. Shinde, C. Lalueza-Fox, M. Meyer, D. Anthony, N. Boivin, K. Thangaraj, D. J. Kennett, M. Frachetti, R. Pinhasi, D. Reich, The formation of human populations in South and Central Asia. Science 365, eaat7487 (2019). 31488661 10.1126/science.aat7487 PMC6822619

[31] X. Zhang, K. E. Witt, M. M. Banuelos, A. Ko, K. Yuan, S. Xu, R. Nielsen, E. Huerta-Sanchez, The history and evolution of the Denisovan-*EPAS1* haplotype in Tibetans. Proc. Natl. Acad. Sci. U.S.A. 118, e2020803118 (2021). 34050022 10.1073/pnas.2020803118 PMC8179186

[32] E. Huerta-Sanchez, X. Jin, Asan, Z. Bianba, B. M. Peter, N. Vinckenbosch, Y. Liang, X. Yi, M. He, M. Somel, P. Ni, B. Wang, X. Ou, Huasang, J. Luosang, Z. X. Cuo, K. Li, G. Gao, Y. Yin, W. Wang, X. Zhang, X. Xu, H. Yang, Y. Li, J. Wang, J. Wang, R. Nielsen, Altitude adaptation in Tibetans caused by introgression of Denisovan-like DNA. Nature 512, 194–197 (2014). 25043035 10.1038/nature13408 PMC4134395

[33] E. Huerta-Sanchez, F. P. Casey, Archaic inheritance: Supporting high-altitude life in Tibet. J. Appl. Physiol. 119, 1129–1134 (2015). 26294746 10.1152/japplphysiol.00322.2015

[34] C. Leipe, T. Long, E. A. Sergusheva, Discontinuous spread of millet agriculture in eastern Asia and prehistoric population dynamics. Sci. Adv. 5, eaax6225 (2019). 31579827 10.1126/sciadv.aax6225 PMC6760930

[35] C. J. Stevens, D. Q. Fuller, The spread of agriculture in eastern Asia. Lang. Dyn. Chang. 7, 152–186 (2017).

[36] M. A. Yang, X. Fan, B. Sun, Ancient DNA indicates human population shifts and admixture in northern and southern China. Science 369, 282–288 (2020). 32409524 10.1126/science.aba0909

[37] G. Dong, X. Jia, R. Elston, F. Chen, S. Li, L. Wang, L. Cai, C. An, Spatial and temporal variety of prehistoric human settlement and its influencing factors in the upper Yellow River valley, Qinghai Province, China. J. Archaeol. Sci. 40, 2538–2546 (2013).

[38] J. V. Bellezza, *The Dawn of Tibet* (Rowman & Littlefield Publishers, 2014).

[39] W. Huo, Tubo archaeology and Tubo civilization. J. Tibet Univ. 24, 57–74 (2009).

[40] H. L. Lu, Z. Y. Li, C. L. Ciren, D. D. Cao, X. Gao, L. H. Li, Sding Chung: An early multi-burial cave site on the Tibetan Plateau. Antiquity 96, 745–753 (2022).

[41] G. Zhaxi, A research review on the Zhang-zhung civilization in the past three decades. J. Sichuan Minzu Coll. 26, (2017).

[42] H. Lu, J. Zhang, Y. Yang, X. Yang, B. Xu, W. Yang, T. Tong, S. Jin, C. Shen, H. Rao, X. Li, H. Lu, D. Q. Fuller, L. Wang, C. Wang, D. Xu, N. Wu, Earliest tea as evidence for one branch of the silk road across the Tibetan Plateau. Sci. Rep. 6, 18955 (2016). 26738699 10.1038/srep18955 PMC4704058

[43] J. Dabney, M. Knapp, I. Glocke, M. T. Gansauge, A. Weihmann, B. Nickel, C. Valdiosera, N. Garcia, S. Paabo, J. L. Arsuaga, M. Meyer, Complete mitochondrial genome sequence of a middle Pleistocene cave bear reconstructed from ultrashort DNA fragments. Proc. Natl. Acad. Sci. U.S.A. 110, 15758–15763 (2013). 24019490 10.1073/pnas.1314445110 PMC3785785

[44] M. Meyer, M. Kircher, M. T. Gansauge, H. Li, F. Racimo, S. Mallick, J. G. Schraiber, F. Jay, K. Prufer, C. de Filippo, P. H. Sudmant, C. Alkan, Q. Fu, R. Do, N. Rohland, A. Tandon, M. Siebauer, R. E. Green, K. Bryc, A. W. Briggs, U. Stenzel, J. Dabney, J. Shendure, J. Kitzman, M. F. Hammer, M. V. Shunkov, A. P. Derevianko, N. Patterson, A. M. Andres, E. E. Eichler, M. Slatkin, D. Reich, J. Kelso, S. Paabo, A high-coverage genome sequence from an archaic Denisovan individual. Science 338, 222–226 (2012). 22936568 10.1126/science.1224344 PMC3617501

[45] M. T. Gansauge, M. Meyer, Single-stranded DNA library preparation for the sequencing of ancient or damaged DNA. Nat. Protoc. 8, 737–748 (2013). 23493070 10.1038/nprot.2013.038

[46] M. Meyer, M. Kircher, Illumina sequencing library preparation for highly multiplexed target capture and sequencing. Cold Spring Harb. Protoc. 2010, pdb.prot5448 (2010). 20516186 10.1101/pdb.prot5448

[47] M. Kircher, S. Sawyer, M. Meyer, Double indexing overcomes inaccuracies in multiplex sequencing on the Illumina platform. Nucleic Acids Res. 40, e3 (2012). 22021376 10.1093/nar/gkr771 PMC3245947

[48] Q. Fu, M. Meyer, X. Gao, U. Stenzel, H. A. Burbano, J. Kelso, S. Pääbo, DNA analysis of an early modern human from Tianyuan cave, China. Proc. Natl. Acad. Sci. U.S.A. 110, 2223–2227 (2013). 23341637 10.1073/pnas.1221359110 PMC3568306

[49] Q. Fu, M. Hajdinjak, O. T. Moldovan, S. Constantin, S. Mallick, P. Skoglund, N. Patterson, N. Rohland, I. Lazaridis, B. Nickel, B. Viola, K. Prufer, M. Meyer, J. Kelso, D. Reich, S. Paabo, An early modern human from Romania with a recent Neanderthal ancestor. Nature 524, 216–219 (2015). 26098372 10.1038/nature14558 PMC4537386

[50] G. Renaud, U. Stenzel, J. Kelso, leeHom: Adaptor trimming and merging for Illumina sequencing reads. Nucleic Acids Res. 42, e141 (2014). 25100869 10.1093/nar/gku699 PMC4191382

[51] H. Li, R. Durbin, Fast and accurate short read alignment with Burrows-Wheeler transform. Bioinformatics 25, 1754–1760 (2009). 19451168 10.1093/bioinformatics/btp324 PMC2705234

[52] Q. Fu, A. Mittnik, P. L. F. Johnson, K. Bos, M. Lari, R. Bollongino, C. Sun, L. Giemsch, R. Schmitz, J. Burger, A. M. Ronchitelli, F. Martini, R. G. Cremonesi, J. Svoboda, P. Bauer, D. Caramelli, S. Castellano, D. Reich, S. Paabo, J. Krause, A revised timescale for human evolution based on ancient mitochondrial genomes. Curr. Biol. 23, 553–559 (2013). 23523248 10.1016/j.cub.2013.02.044 PMC5036973

[53] T. S. Korneliussen, A. Albrechtsen, R. Nielsen, ANGSD: Analysis of next generation sequencing data. BMC Bioinformatics 15, 356 (2014). 25420514 10.1186/s12859-014-0356-4 PMC4248462

[54] P. J. Reimer, W. E. N. Austin, E. Bard, The IntCal20 northern hemisphere radiocarbon age calibration curve (0–55 cal kBP). Radiocarbon 62, 725–757 (2020).

[55] P. Skoglund, J. Storå, A. Götherström, M. Jakobsson, Accurate sex identification of ancient human remains using DNA shotgun sequencing. J. Archaeol. Sci. 40, 4477–4482 (2013).

[56] M. van Oven, M. Kayser, Updated comprehensive phylogenetic tree of global human mitochondrial DNA variation. Hum. Mutat. 30, E386–E394 (2009). 18853457 10.1002/humu.20921

[57] H. Weissensteiner, D. Pacher, A. Kloss-Brandstätter, L. Forer, G. Specht, H.-J. Bandelt, F. Kronenberg, A. Salas, S. Schönherr, HaploGrep 2: Mitochondrial haplogroup classification in the era of high-throughput sequencing. Nucleic Acids Res. 44, W58–W63 (2016). 27084951 10.1093/nar/gkw233 PMC4987869

[58] M. S. Blouin, DNA-based methods for pedigree reconstruction and kinship analysis in natural populations. Trends Ecol. Evol. 18, 503–511 (2003).

[59] S. Mallick, H. Li, M. Lipson, I. Mathieson, M. Gymrek, F. Racimo, M. Zhao, N. Chennagiri, S. Nordenfelt, A. Tandon, P. Skoglund, I. Lazaridis, S. Sankararaman, Q. Fu, N. Rohland, G. Renaud, Y. Erlich, T. Willems, C. Gallo, J. P. Spence, Y. S. Song, G. Poletti, F. Balloux, G. van Driem, P. de Knijff, I. G. Romero, A. R. Jha, D. M. Behar, C. M. Bravi, C. Capelli, T. Hervig, A. Moreno-Estrada, O. L. Posukh, E. Balanovska, O. Balanovsky, S. Karachanak-Yankova, H. Sahakyan, D. Toncheva, L. Yepiskoposyan, C. Tyler-Smith, Y. Xue, M. S. Abdullah, A. Ruiz-Linares, C. M. Beall, A. Di Rienzo, C. Jeong, E. B. Starikovskaya, E. Metspalu, J. Parik, R. Villems, B. M. Henn, U. Hodoglugil, R. Mahley, A. Sajantila, G. Stamatoyannopoulos, J. T. S. Wee, R. Khusainova, E. Khusnutdinova, S. Litvinov, G. Ayodo, D. Comas, M. F. Hammer, T. Kivisild, W. Klitz, C. A. Winkler, D. Labuda, M. Bamshad, L. B. Jorde, S. A. Tishkoff, W. S. Watkins, M. Metspalu, S. Dryomov, R. Sukernik, L. Singh, K. Thangaraj, S. Pääbo, J. Kelso, N. Patterson, D. Reich, The Simons genome diversity project: 300 genomes from 142 diverse populations. Nature 538, 201–206 (2016). 27654912 10.1038/nature18964 PMC5161557

[60] S. Purcell, B. Neale, K. Todd-Brown, L. Thomas, M. A. Ferreira, D. Bender, J. Maller, P. Sklar, P. I. De Bakker, M. J. Daly, PLINK: A tool set for whole-genome association and population-based linkage analyses. Am. J. Hum. Genet. 81, 559–575 (2007). 17701901 10.1086/519795 PMC1950838

[61] P. Skoglund, C. Posth, K. Sirak, M. Spriggs, F. Valentin, S. Bedford, G. R. Clark, C. Reepmeyer, F. Petchey, D. Fernandes, Q. Fu, E. Harney, M. Lipson, S. Mallick, M. Novak, N. Rohland, K. Stewardson, S. Abdullah, M. P. Cox, F. R. Friedlaender, J. S. Friedlaender, T. Kivisild, G. Koki, P. Kusuma, D. A. Merriwether, F. X. Ricaut, J. T. Wee, N. Patterson, J. Krause, R. Pinhasi, D. Reich, Genomic insights into the peopling of the Southwest Pacific. Nature 538, 510–513 (2016). 27698418 10.1038/nature19844 PMC5515717

[62] P. Damgaard, N. Marchi, S. Rasmussen, M. Peyrot, G. Renaud, T. Korneliussen, J. V. Moreno-Mayar, M. W. Pedersen, A. Goldberg, E. Usmanova, N. Baimukhanov, V. Loman, L. Hedeager, A. G. Pedersen, K. Nielsen, G. Afanasiev, K. Akmatov, A. Aldashev, A. Alpaslan, G. Baimbetov, V. I. Bazaliiskii, A. Beisenov, B. Boldbaatar, B. Boldgiv, C. Dorzhu, S. Ellingvag, D. Erdenebaatar, R. Dajani, E. Dmitriev, V. Evdokimov, K. M. Frei, A. Gromov, A. Goryachev, H. Hakonarson, T. Hegay, Z. Khachatryan, R. Khaskhanov, E. Kitov, A. Kolbina, T. Kubatbek, A. Kukushkin, I. Kukushkin, N. Lau, A. Margaryan, I. Merkyte, I. V. Mertz, V. K. Mertz, E. Mijiddorj, V. Moiyesev, G. Mukhtarova, B. Nurmukhanbetov, Z. Orozbekova, I. Panyushkina, K. Pieta, V. Smrcka, I. Shevnina, A. Logvin, K. G. Sjogren, T. Stolcova, K. Tashbaeva, A. Tkachev, T. Tulegenov, D. Voyakin, L. Yepiskoposyan, S. Undrakhbold, V. Varfolomeev, A. Weber, N. Kradin, M. E. Allentoft, L. Orlando, R. Nielsen, M. Sikora, E. Heyer, K. Kristiansen, E. Willerslev, 137 ancient human genomes from across the Eurasian steppes. Nature 557, 369–374 (2018). 29743675 10.1038/s41586-018-0094-2

[63] P. Damgaard, R. Martiniano, J. Kamm, J. V. Moreno-Mayar, G. Kroonen, M. Peyrot, G. Barjamovic, S. Rasmussen, C. Zacho, N. Baimukhanov, V. Zaibert, V. Merz, A. Biddanda, I. Merz, V. Loman, V. Evdokimov, E. Usmanova, B. Hemphill, A. Seguin-Orlando, F. E. Yediay, I. Ullah, K. G. Sjogren, K. H. Iversen, J. Choin, C. de la Fuente, M. Ilardo, H. Schroeder, V. Moiseyev, A. Gromov, A. Polyakov, S. Omura, S. Y. Senyurt, H. Ahmad, C. McKenzie, A. Margaryan, A. Hameed, A. Samad, N. Gul, M. H. Khokhar, O. I. Goriunova, V. I. Bazaliiskii, J. Novembre, A. W. Weber, L. Orlando, M. E. Allentoft, R. Nielsen, K. Kristiansen, M. Sikora, A. K. Outram, R. Durbin, E. Willerslev, The first horse herders and the impact of early bronze age steppe expansions into Asia. Science 360, eaar7711 (2018). 29743352 10.1126/science.aar7711 PMC6748862

[64] M. Rasmussen, S. L. Anzick, M. R. Waters, P. Skoglund, M. DeGiorgio, T. W. Stafford Jr., S. Rasmussen, I. Moltke, A. Albrechtsen, S. M. Doyle, G. D. Poznik, V. Gudmundsdottir, R. Yadav, A.-S. Malaspinas, S. S. White, M. E. Allentoft, O. E. Cornejo, K. Tambets, A. Eriksson, P. D. Heintzman, M. Karmin, T. S. Korneliussen, D. J. Meltzer, T. L. Pierre, J. Stenderup, L. Saag, V. M. Warmuth, M. C. Lopes, R. S. Malhi, S. Brunak, T. Sicheritz-Ponten, I. Barnes, M. Collins, L. Orlando, F. Balloux, A. Manica, R. Gupta, M. Metspalu, C. D. Bustamante, M. Jakobsson, R. Nielsen, E. Willerslev, The genome of a Late Pleistocene human from a Clovis burial site in western Montana. Nature 506, 225, 229 (2014). 24522598 10.1038/nature13025 PMC4878442

[65] C. Ning, T. Li, K. Wang, F. Zhang, T. Li, X. Wu, S. Gao, Q. Zhang, H. Zhang, M. J. Hudson, G. Dong, S. Wu, Y. Fang, C. Liu, C. Feng, W. Li, T. Han, R. Li, J. Wei, Y. Zhu, Y. Zhou, C. C. Wang, S. Fan, Z. Xiong, Z. Sun, M. Ye, L. Sun, X. Wu, F. Liang, Y. Cao, X. Wei, H. Zhu, H. Zhou, J. Krause, M. Robbeets, C. Jeong, Y. Cui, Ancient genomes from northern China suggest links between subsistence changes and human migration. Nat. Commun. 11, 2700 (2020). 32483115 10.1038/s41467-020-16557-2 PMC7264253

[66] É. Harney, N. Patterson, D. Reich, J. Wakeley, Assessing the performance of qpAdm: A statistical tool for studying population admixture. bioRxiv, 2020.04.09.032664 (2020). 10.1093/genetics/iyaa045 PMC8049561 33772284

[67] B. M. Peter, 100,000 years of gene flow between Neandertals and Denisovans in the Altai mountains. bioRxiv, 2020.03.13.990523 (2020).

[68] K. Prufer, F. Racimo, N. Patterson, F. Jay, S. Sankararaman, S. Sawyer, A. Heinze, G. Renaud, P. H. Sudmant, C. de Filippo, H. Li, S. Mallick, M. Dannemann, Q. Fu, M. Kircher, M. Kuhlwilm, M. Lachmann, M. Meyer, M. Ongyerth, M. Siebauer, C. Theunert, A. Tandon, P. Moorjani, J. Pickrell, J. C. Mullikin, S. H. Vohr, R. E. Green, I. Hellmann, P. L. Johnson, H. Blanche, H. Cann, J. O. Kitzman, J. Shendure, E. E. Eichler, E. S. Lein, T. E. Bakken, L. V. Golovanova, V. B. Doronichev, M. V. Shunkov, A. P. Derevianko, B. Viola, M. Slatkin, D. Reich, J. Kelso, S. Paabo, The complete genome sequence of a Neanderthal from the Altai Mountains. Nature 505, 43–49 (2014). 24352235 10.1038/nature12886 PMC4031459

[69] K. Prüfer, C. d. Filippo, S. Grote, F. Mafessoni, A high-coverage Neandertal genome from Vindija cave in Croatia. Science 358, 4 (2017). 10.1126/science.aao1887 PMC6185897 28982794

[70] Y. Wang, F. Song, J. Zhu, S. Zhang, Y. Yang, T. Chen, B. Tang, L. Dong, N. Ding, Q. Zhang, Z. Bai, X. Dong, H. Chen, M. Sun, S. Zhai, Y. Sun, L. Yu, L. Lan, J. Xiao, X. Fang, H. Lei, Z. Zhang, W. Zhao, GSA: Genome sequence archive. Genomics Proteomics Bioinformatics 15, 14–18 (2017). 28387199 10.1016/j.gpb.2017.01.001 PMC5339404

[71] Big Data Center Members, Database resources of the BIG data center in 2018. Nucleic Acids Res. 46, D14–D20 (2018). 29036542 10.1093/nar/gkx897 PMC5753194

[72] H. H. Chen, “A study of the Zongri remains,” thesis, Peking University (Chinese) (1992).

[73] X. Guo, “The study of burial potteries from Zongri relic,” thesis, Xibei University (Chinese) (2014).

[74] Y. Liu, “Archaeobotanical study in Zongri Site, Qinghai Province, China,” thesis, Lanzhou University (Chinese) (2018).

[75] X. Y. Ren, Y. H. He, Q. W. Zhao, S. C. Pan, M. Tang, J. Y. Cao, C. Huang, H. Jiang, D. Tan, Y. M. Wang, L. H. Cai, X. J. Gu, Y. Song, Y. Qin, W. Du, C. Y. Ma, P. Li, D. J. Lu, Y. X. Zheng, X. H. Yu, The brief survery report of Pukagongma sarcophagus tomb in Zhiduo County, Qinghai Province. J. Tibet. 2017, 24–40 (2017).

[76] L. L. Ren, G. H. Dong, H. M. Li, D. Rhode, R. K. Flad, G. Q. Li, Y. Yang, Z. X. Wang, L. H. Cai, X. Y. Ren, D. J. Zhang, F. H. Chen, Dating human settlement in the east-central Tibetan Plateau during the Late Holocene. 60, 137–150 (2018).

[77] Z. Zhang, W. Xiage, H. Lv, C. n. Sodnam, Identification and interpretation of faunal remains from a prehistoric cist burial in Amdo County, North Tibet. Journal of Tibetology (Chinese), 1–18 (2015).

[78] W. Xiage, “Unveiling the veil of ancient civilization in Qiangtang Plateau,” *Journal of Tibet University (Chinese)* v01.20.NO.1, (2005).

[79] Y. Xiao, “Remains of tombs from the early 7th century to the early 8th century were found in bange County, Tibet Autonomous Region,” *Tibet Daily (Chinese)* (2019).

[80] X. M. C. H. Bureau, D. o. A. o. S. University, S. P. I. o. Archaeology, *Field Archaeology Report of Tibetan Section of Qinghai-Tibet Railway* (Science Press (Chinese), 2005).

[81] H. Wang, Y. Li, Y. Feng, L. Tu, H. Zhang, The age of human bones from Redilong, Qamdo County, Tibet. Acta Geoscinet. Sin. 24, 569–572 (2003).

[82] W. Xiage, Tentative analysis of the type and age of the prehistoric stone coffin burials in Tibet. Tibet Stud. v4, 40–44 (1998).

[83] X. M. C. H. Committee, The preliminary report of Xiaoenda Neolithic age ruins in Xizang autonomous region. *Archaeology and Relics (Chinese) *v1, (1990).

[84] Anonymous, “Agongrong cemetery in Bomi, Tibet.,” *Popular Archaeology (Chinese)* v4 (2017).

[85] Xinhuanet, “The largest number of pottery tombs unearthed in Shannan, Tibet,” *Identification and Appreciation to Cultural Relics (Chinese)* v6 (2018).

[86] Z. D. S. He, Wangdui, “Discovery of ancient tombs in Jiesang village, Naidong, Tibet,” *Archaeology (Chinese)* v12 (1985).

[87] Z. Luobu, “New discovery and preliminary understanding of early tombs in central Tibet,” *The second silk road archaeological Forum (Chinese Meeting)* (2018).

[88] F. B. Jiang, “Middle and late Tubo tombs discovered in Tibet,” *China News Service (Chinese)* (2018).

[89] Archaeological investigation and excavation report of water control project in Pundo County Linzhou City, in *Research on Cultural Relics and Archaeology in Tibet,* Habibu, F. Xu, L. Dunzhu, Eds. (Science Press, ed. 1, 2016), chap. 1, pp. 282.

[90] H. Wei, “Archaeological discovery and research of prehistoric tombs in Tibet plateau,” China Tibet v4 (1994).

[91] S. Wang, “Archaeological discoveries “outline” the prehistoric civilization of Western Tibet,” China Tibet Online (Chinese) (2018).

[92] X. Yu, “Archaeological discoveries and characteristics of funeral customs in Western Tibet in the pre Tubo era,” Journal of Northwest Minzu University 1 (2017).

[93] Y. X. Li, “A brief report on the investigation of Gebusailu cemetery in Zhada County, Tibet,” Archaeology (Chinese) v6 (2001).

[94] J. H. Yao, W, “A brief report on the excavation of ancient tombs at the piyang Dongga site in Zhada County, Tibet,” *Archaeology (Chinese)* v6 (2001).

[95] A. Bergstrom, S. A. McCarthy, R. Hui, M. A. Almarri, Q. Ayub, P. Danecek, Y. Chen, S. Felkel, P. Hallast, J. Kamm, H. Blanche, J. F. Deleuze, H. Cann, S. Mallick, D. Reich, M. S. Sandhu, P. Skoglund, A. Scally, Y. Xue, R. Durbin, C. Tyler-Smith, Insights into human genetic variation and population history from 929 diverse genomes. Science 367, eaay5012 (2020). 32193295 10.1126/science.aay5012 PMC7115999

[96] V. M. Narasimhan, N. Patterson, P. Moorjani, I. Lazaridis, L. Mark, S. Mallick, N. Rohland, R. Bernardos, A. Kim, N. Nakatsuka, I. Olalde, A. Coppa, J. Mallory, V. Moiseyev, J. Monge, L. Olivieri, N. Adamski, N. Broomandkhoshbacht, F. Candilio, O. Cheronet, B. Culleton, M. Ferry, D. Fernandes, B. Gamarra, D. Gaudio, M. Hajdinjak, E. Harney, T. Harper, D. Keating, A. Lawson, M. Michel, M. Novak, J. Oppenheimer, N. Rai, K. Sirak, V. Slon, K. Stewardson, Z. Zhang, G. Akhatov, A. Bagashev, B. Baitanayev, G. Bonora, T. Chikisheva, A. Derevianko, E. Dmitry, K. Douka, N. Dubova, A. Epimakhov, S. Freilich, D. Fuller, A. Goryachev, A. Gromov, B. Hanks, M. Judd, E. Kazizov, A. Khokhlov, E. Kitov, E. Kupriyanova, P. Kuznetsov, D. Luiselli, F. Maksudov, C. Meiklejohn, D. Merrett, R. Micheli, O. Mochalov, Z. Muhammed, S. Mustafakulov, A. Nayak, R. Petrovna, D. Pettner, R. Potts, D. Razhev, S. Sarno, K. Sikhymbaevae, S. Slepchenko, N. Stepanova, S. Svyatko, S. Vasilyev, M. Vidale, D. Voyakin, A. Yermolayeva, A. Zubova, V. Shinde, C. Lalueza-Fox, M. Meyer, D. Anthony, N. Boivin, K. Thangaraj, D. Kennett, M. Frachetti, R. Pinhasi, D. Reich, The genomic formation of South and Central Asia. bioRxiv 10.1101/292581 [Preprint]. 31 March 2018. 10.1101/292581.

[97] H. McColl, F. Racimo, L. Vinner, F. Demeter, T. Gakuhari, J. V. Moreno-Mayar, G. van Driem, U. Gram Wilken, A. Seguin-Orlando, C. de la Fuente Castro, S. Wasef, R. Shoocongdej, V. Souksavatdy, T. Sayavongkhamdy, M. M. Saidin, M. E. Allentoft, T. Sato, A. S. Malaspinas, F. A. Aghakhanian, T. Korneliussen, A. Prohaska, A. Margaryan, P. de Barros Damgaard, S. Kaewsutthi, P. Lertrit, T. M. H. Nguyen, H. C. Hung, T. Minh Tran, H. Nghia Truong, G. H. Nguyen, S. Shahidan, K. Wiradnyana, H. Matsumae, N. Shigehara, M. Yoneda, H. Ishida, T. Masuyama, Y. Yamada, A. Tajima, H. Shibata, A. Toyoda, T. Hanihara, S. Nakagome, T. Deviese, A. M. Bacon, P. Duringer, J. L. Ponche, L. Shackelford, E. Patole-Edoumba, A. T. Nguyen, B. Bellina-Pryce, J. C. Galipaud, R. Kinaston, H. Buckley, C. Pottier, S. Rasmussen, T. Higham, R. A. Foley, M. M. Lahr, L. Orlando, M. Sikora, M. E. Phipps, H. Oota, C. Higham, D. M. Lambert, E. Willerslev, The prehistoric peopling of Southeast Asia. Science 361, 88–92 (2018). 29976827 10.1126/science.aat3628

[98] T. Wang, W. Wang, G. Xie, Human population history at the crossroads of east and Southeast Asia since 11,000 years ago. Cell 184, 3829–3841.e21 (2021). 34171307 10.1016/j.cell.2021.05.018

[99] P. Skoglund, J. C. Thompson, M. E. Prendergast, A. Mittnik, K. Sirak, M. Hajdinjak, T. Salie, N. Rohland, S. Mallick, A. Peltzer, A. Heinze, I. Olalde, M. Ferry, E. Harney, M. Michel, K. Stewardson, J. I. Cerezo-Roman, C. Chiumia, A. Crowther, E. Gomani-Chindebvu, A. O. Gidna, K. M. Grillo, I. T. Helenius, G. Hellenthal, R. Helm, M. Horton, S. Lopez, A. Z. P. Mabulla, J. Parkington, C. Shipton, M. G. Thomas, R. Tibesasa, M. Welling, V. M. Hayes, D. J. Kennett, R. Ramesar, M. Meyer, S. Paabo, N. Patterson, A. G. Morris, N. Boivin, R. Pinhasi, J. Krause, D. Reich, Reconstructing prehistoric African population structure. Cell 171, 59–71.e21 (2017). 28938123 10.1016/j.cell.2017.08.049 PMC5679310

[100] I. Lazaridis, N. Patterson, A. Mittnik, G. Renaud, S. Mallick, K. Kirsanow, P. H. Sudmant, J. G. Schraiber, S. Castellano, M. Lipson, B. Berger, C. Economou, R. Bollongino, Q. Fu, K. I. Bos, S. Nordenfelt, H. Li, C. de Filippo, K. Prufer, S. Sawyer, C. Posth, W. Haak, F. Hallgren, E. Fornander, N. Rohland, D. Delsate, M. Francken, J. M. Guinet, J. Wahl, G. Ayodo, H. A. Babiker, G. Bailliet, E. Balanovska, O. Balanovsky, R. Barrantes, G. Bedoya, H. Ben-Ami, J. Bene, F. Berrada, C. M. Bravi, F. Brisighelli, G. B. Busby, F. Cali, M. Churnosov, D. E. Cole, D. Corach, L. Damba, G. van Driem, S. Dryomov, J. M. Dugoujon, S. A. Fedorova, I. Gallego Romero, M. Gubina, M. Hammer, B. M. Henn, T. Hervig, U. Hodoglugil, A. R. Jha, S. Karachanak-Yankova, R. Khusainova, E. Khusnutdinova, R. Kittles, T. Kivisild, W. Klitz, V. Kucinskas, A. Kushniarevich, L. Laredj, S. Litvinov, T. Loukidis, R. W. Mahley, B. Melegh, E. Metspalu, J. Molina, J. Mountain, K. Nakkalajarvi, D. Nesheva, T. Nyambo, L. Osipova, J. Parik, F. Platonov, O. Posukh, V. Romano, F. Rothhammer, I. Rudan, R. Ruizbakiev, H. Sahakyan, A. Sajantila, A. Salas, E. B. Starikovskaya, A. Tarekegn, D. Toncheva, S. Turdikulova, I. Uktveryte, O. Utevska, R. Vasquez, M. Villena, M. Voevoda, C. A. Winkler, L. Yepiskoposyan, P. Zalloua, T. Zemunik, A. Cooper, C. Capelli, M. G. Thomas, A. Ruiz-Linares, S. A. Tishkoff, L. Singh, K. Thangaraj, R. Villems, D. Comas, R. Sukernik, M. Metspalu, M. Meyer, E. E. Eichler, J. Burger, M. Slatkin, S. Paabo, J. Kelso, D. Reich, J. Krause, Ancient human genomes suggest three ancestral populations for present-day Europeans. Nature 513, 409–413 (2014). 25230663 10.1038/nature13673 PMC4170574

[101] S. R. Grossman, I. Shlyakhter, E. K. Karlsson, E. H. Byrne, S. Morales, G. Frieden, E. Hostetter, E. Angelino, M. Garber, O. Zuk, E. S. Lander, S. F. Schaffner, P. C. Sabeti, A composite of multiple signals distinguishes causal variants in regions of positive selection. Science 327, 883–886 (2010). 20056855 10.1126/science.1183863

[102] A. Fujimoto, J. Ohashi, N. Nishida, T. Miyagawa, Y. Morishita, T. Tsunoda, R. Kimura, K. Tokunaga, A replication study confirmed the EDAR gene to be a major contributor to population differentiation regarding head hair thickness in Asia. Hum. Genet. 124, 179–185 (2008). 18704500 10.1007/s00439-008-0537-1

[103] Y. G. Kamberov, S. Wang, J. Tan, P. Gerbault, A. Wark, L. Tan, Y. Yang, S. Li, K. Tang, H. Chen, A. Powell, Y. Itan, D. Fuller, J. Lohmueller, J. Mao, A. Schachar, M. Paymer, E. Hostetter, E. Byrne, M. Burnett, A. P. McMahon, M. G. Thomas, D. E. Lieberman, L. Jin, C. J. Tabin, B. A. Morgan, P. C. Sabeti, Modeling recent human evolution in mice by expression of a selected EDAR variant. Cell 152, 691–702 (2013). 23415220 10.1016/j.cell.2013.01.016 PMC3575602

[104] J. H. Park, T. Yamaguchi, C. Watanabe, A. Kawaguchi, K. Haneji, M. Takeda, Y. I. Kim, Y. Tomoyasu, M. Watanabe, H. Oota, T. Hanihara, H. Ishida, K. Maki, S. B. Park, R. Kimura, Effects of an Asian-specific nonsynonymous EDAR variant on multiple dental traits. J. Hum. Genet. 57, 508–514 (2012). 22648185 10.1038/jhg.2012.60

[105] R. A. Sturm, D. L. Duffy, Z. Z. Zhao, F. P. Leite, M. S. Stark, N. K. Hayward, N. G. Martin, G. W. Montgomery, A single SNP in an evolutionary conserved region within intron 86 of the HERC2 gene determines human blue-brown eye color. Am. J. Hum. Genet. 82, 424–431 (2008). 18252222 10.1016/j.ajhg.2007.11.005 PMC2427173

[106] C. A. Guenther, B. Tasic, L. Luo, M. A. Bedell, D. M. Kingsley, A molecular basis for classic blond hair color in Europeans. Nat. Genet. 46, 748–752 (2014). 24880339 10.1038/ng.2991 PMC4704868

[107] F. Liu, K. van Duijn, J. R. Vingerling, A. Hofman, A. G. Uitterlinden, A. C. Janssens, M. Kayser, Eye color and the prediction of complex phenotypes from genotypes. Curr. Biol. 19, R192–R193 (2009). 19278628 10.1016/j.cub.2009.01.027

[108] F. Liu, A. Wollstein, P. G. Hysi, G. A. Ankra-Badu, T. D. Spector, D. Park, G. Zhu, M. Larsson, D. L. Duffy, G. W. Montgomery, D. A. Mackey, S. Walsh, O. Lao, A. Hofman, F. Rivadeneira, J. R. Vingerling, A. G. Uitterlinden, N. G. Martin, C. J. Hammond, M. Kayser, Digital quantification of human eye color highlights genetic association of three new loci. PLOS Genet. 6, e1000934 (2010). 20463881 10.1371/journal.pgen.1000934 PMC2865509

[109] R. L. Lamason, M. A. Mohideen, J. R. Mest, A. C. Wong, H. L. Norton, M. C. Aros, M. J. Jurynec, X. Mao, V. R. Humphreville, J. E. Humbert, S. Sinha, J. L. Moore, P. Jagadeeswaran, W. Zhao, G. Ning, I. Makalowska, P. M. McKeigue, D. O'Donnell, R. Kittles, E. J. Parra, N. J. Mangini, D. J. Grunwald, M. D. Shriver, V. A. Canfield, K. C. Cheng, SLC24A5, a putative cation exchanger, affects pigmentation in zebrafish and humans. Science 310, 1782–1786 (2005). 16357253 10.1126/science.1116238

[110] M. Soejima, Y. Koda, Population differences of two coding SNPs in pigmentation-related genes SLC24A5 and SLC45A2. Int. J. Leg. Med. 121, 36–39 (2007). 10.1007/s00414-006-0112-z 16847698

[111] S. Walsh, F. Liu, A. Wollstein, L. Kovatsi, A. Ralf, A. Kosiniak-Kamysz, W. Branicki, M. Kayser, The HIrisPlex system for simultaneous prediction of hair and eye colour from DNA. Forensic Sci. Int. Genet. 7, 98–115 (2013). 22917817 10.1016/j.fsigen.2012.07.005

[112] P. Sulem, D. F. Gudbjartsson, S. N. Stacey, A. Helgason, T. Rafnar, K. P. Magnusson, A. Manolescu, A. Karason, A. Palsson, G. Thorleifsson, M. Jakobsdottir, S. Steinberg, S. Palsson, F. Jonasson, B. Sigurgeirsson, K. Thorisdottir, R. Ragnarsson, K. R. Benediktsdottir, K. K. Aben, L. A. Kiemeney, J. H. Olafsson, J. Gulcher, A. Kong, U. Thorsteinsdottir, K. Stefansson, Genetic determinants of hair, eye and skin pigmentation in Europeans. Nat. Genet. 39, 1443–1452 (2007). 17952075 10.1038/ng.2007.13

[113] N. S. Enattah, T. Sahi, E. Savilahti, J. D. Terwilliger, L. Peltonen, I. Jarvela, Identification of a variant associated with adult-type hypolactasia. Nat. Genet. 30, 233–237 (2002). 11788828 10.1038/ng826

[114] C. J. Ingram, M. F. Elamin, C. A. Mulcare, M. E. Weale, A. Tarekegn, T. O. Raga, E. Bekele, F. M. Elamin, M. G. Thomas, N. Bradman, D. M. Swallow, A novel polymorphism associated with lactose tolerance in Africa: Multiple causes for lactase persistence? Hum. Genet. 120, 779–788 (2007). 17120047 10.1007/s00439-006-0291-1

[115] S. A. Tishkoff, F. A. Reed, A. Ranciaro, B. F. Voight, C. C. Babbitt, J. S. Silverman, K. Powell, H. M. Mortensen, J. B. Hirbo, M. Osman, M. Ibrahim, S. A. Omar, G. Lema, T. B. Nyambo, J. Ghori, S. Bumpstead, J. K. Pritchard, G. A. Wray, P. Deloukas, Convergent adaptation of human lactase persistence in Africa and Europe. Nat. Genet. 39, 31–40 (2007). 17159977 10.1038/ng1946 PMC2672153

[116] K. Eaton, M. Edwards, S. Krithika, G. Cook, H. Norton, E. J. Parra, Association study confirms the role of two OCA2 polymorphisms in normal skin pigmentation variation in East Asian populations. Am. J. Hum. Biol. 27, 520–525 (2015). 25809079 10.1002/ajhb.22678

[117] H. Rajeevan, U. Soundararajan, J. R. Kidd, A. J. Pakstis, K. K. Kidd, ALFRED: An allele frequency resource for research and teaching. Nucleic Acids Res. 40, D1010–D1015 (2012). 22039151 10.1093/nar/gkr924 PMC3245092

[118] P. J. Brooks, M. A. Enoch, D. Goldman, T. K. Li, A. Yokoyama, The alcohol flushing response: An unrecognized risk factor for esophageal cancer from alcohol consumption. PLOS Med. 6, e50 (2009). 19320537 10.1371/journal.pmed.1000050 PMC2659709

[119] A. Yoshida, I. Y. Huang, M. Ikawa, Molecular abnormality of an inactive aldehyde dehydrogenase variant commonly found in Orientals. Proc. Natl. Acad. Sci. U.S.A. 81, 258–261 (1984). 6582480 10.1073/pnas.81.1.258 PMC344651

[120] H. Li, S. Borinskaya, K. Yoshimura, N. Kal'ina, A. Marusin, V. A. Stepanov, Z. Qin, S. Khaliq, M. Y. Lee, Y. Yang, A. Mohyuddin, D. Gurwitz, S. Q. Mehdi, E. Rogaev, L. Jin, N. K. Yankovsky, J. R. Kidd, K. K. Kidd, Refined geographic distribution of the oriental *ALDH2*504Lys* (nee *487Lys*) variant. Ann. Hum. Genet. 73, 335–345 (2009). 19456322 10.1111/j.1469-1809.2009.00517.x PMC2846302

[121] H. Li, N. Mukherjee, U. Soundararajan, Z. Tarnok, C. Barta, S. Khaliq, A. Mohyuddin, S. L. Kajuna, S. Q. Mehdi, J. R. Kidd, K. K. Kidd, Geographically separate increases in the frequency of the derived ADH1B*47His allele in eastern and western Asia. Am. J. Hum. Genet. 81, 842–846 (2007). 17847010 10.1086/521201 PMC2227934

[122] Y. Peng, H. Shi, X. B. Qi, C. J. Xiao, H. Zhong, R. L. Ma, B. Su, The ADH1B Arg47His polymorphism in east Asian populations and expansion of rice domestication in history. BMC Evol. Biol. 10, 15 (2010). 20089146 10.1186/1471-2148-10-15 PMC2823730

