# Supplementary material for: Human genetic history on the Tibetan Plateau in the past 5100 years
Source: Sci Adv. 2023 Mar 17;9(11):eadd5582. doi: 10.1126/sciadv.add5582 (PMC10022901; doi:10.1126/sciadv.add5582)
Supplement: Supplementary file 1 — Supplementary Text Figs. S1 to S33 Legends for tables S1 to S3, S8 to S10, S14, S15, S17, S20, S21, S23 Tables S4 to S7, S11 to S13, S16, S18, S19, S22 References [file sciadv.add5582_sm.pdf]

Science Advances
AAAS

Supplementary Materials for

Human genetic history on the Tibetan Plateau in the past 5100 years

Hongru Wang *et al.*

Corresponding author: Qiaomei Fu, fuqiaomei@ivpp.ac.cn

Sci. Adv. **9**, eadd5582 (2023)
DOI: 10.1126/sciadv.add5582

The PDF file includes:

Supplementary Text
Figs. S1 to S33
Legends for tables S1 to S3, S8 to S10, S14, S15, S17, S20, S21, S23
Tables S4 to S7, S11 to S13, S16, S18, S19, S22
References

Other Supplementary Material for this manuscript includes the following:

Tables S1 to S3, S8 to S10, S14, S15, S17, S20, S21, S23

Supplementary Text

Samples description

Overview

In this study, we obtained 97 human specimens from 30 archaeological sites spanning across the Tibetan Plateau, including both the Tibet Autonomous Region (Xizang) and Qinghai Province of China. It covers all seven prefecture-level divisions of Tibet Autonomous Region, i.e. Ngari, Shigatse, Lhasa, Shannan, Nagqu, Nyingchi and Chamdo; and two prefectures of Qinghai, i.e., Yushu and Hainan. Sites were grouped into six sub-regions as defined in the Methods. The locations of archaeological sites are listed below:

- *northeastern Tibetan Plateau*: Zongri, Pukagongma and Galacun sites;
- *central Tibetan Plateau*: Ousui, Butaxiongqu, Ounie, Gangre and Chaxiutang sites;
- *southeastern Tibetan Plateau*: Redilong, Xiaoenda, Agangrong, Kangyu and Gutong sites;
- *southern Tibetan Plateau*: Luozhating, Jiesang, Yusa, Nudagang, Dama, Shigou, Rangjun, Longsangquduo, Latuotanggu, Jawutang, Gachong and Lajue sites.
- *western Tibetan Plateau*: Piyangjiweng and Gelintang.
- *southwestern Tibetan Plateau*: Sila, Sding Chung and Zhangshu sites.

Zongri site from the Hainan prefecture, Qinghai

We analyzed 22 human samples from the Zongri site, from the Hainan prefecture in Qinghai province. We retained 12 unrelated individuals, who are clustered into six genetic groups (Supplementary Text). Outgroup-f_3 statistics show that these groups share the highest genetic drift with present-day populations on or near the Tibetan Plateau (Fig. S2), especially the ones from the southeastern Tibetan Plateau.

Zongri (n = 22, the number of specimens examined)

The Zongri site is located at an elevation of 2800–3000 meters above sea level (masl) in Tongde County, Hainan Prefecture, Qinghai. The site was first discovered in 1982,

followed by an exploratory excavation in 1983 and major excavations in 1994 and 1996 (*23*). The pottery excavated at the Zongri site are related to three different cultures: the local Zongri Culture, the Majiayao Culture (5,300-4,000 BP) from neighboring regions to the east, and the Qijia Culture (*23, 73*). The Zongri Culture overlapped in time with the Majiayao Culture, which was succeeded by the Qijia Culture (*74*). The 22 human samples included in this study are from different burials which cover four temporal phases of this site (*75*). From phase I burials, we sampled C4783 and C202 from Grave M297 who was buried with early Zongri style pottery, C056 from Grave M299 buried with Majiayao style pottery, and C208 from Grave M273 with pottery related to both the Zongri and Majiayao Cultures. From phase II-III burials, we sampled CSP046 from Grave M27, who was buried with one Majiayao style pot and several Zongri style pots. We also sampled CSP048 from Grave M33, C050 from Grave M1, CSP047 from Grave M75, and CSP057 from Grave M22, and these graves contain typical Zongri style potteries. From phase IV burials, we sampled C205 and C4775 from Grave M32, CSP049 from Grave M80, and these two graves contained pottery likely related to the Qijia Culture. Ten specimens were sampled from burials with unassigned phases: C051 from Grave M251, CSP054 and C4776 from Grave M78, C4782 and C4778 from Grave M223, C4777 and C4780 from Grave M225, C4779 from Grave M224, C4781 from Grave M226, and C4774 from Grave M14. Radiocarbon dates were generated for 13 specimens, which showed they lived 5213-3716 calibrated years before present (cal BP, Table S2).

In a familial analysis (Table S4), we found two pairs were identified as deriving from the same individual ("Self"). C4783 and C202 are from the same grave, and assuming the two specimens derived from the same individual, we merged their genomic data (denoted hereafter as C4783_C202). In contrast, the other pair, C205 and C4777, are from different graves. We thus only kept the one with greater endogenous DNA (C205) for downstream analysis. We found seven pairs of familial relationships, and for each pair, we retained only the one with greater endogenous DNA for downstream population genetic analysis. We additionally found that CSP049 showed high evidence of contamination and thus data from this specimen was excluded from further analysis (Table S1). After filtering, 12 of 20

successfully sequenced specimens were identified as deriving from distinct individuals and were retained for population genetic analysis.

Yushu prefecture, Qinghai

We analyzed six humans from two sites, Pukagongma and Galacun from the Yushu prefecture in Qinghai province. We retained six unrelated individuals, who are clustered into two genetic groups, Yushu2.8k and Yushu0.5k, for downstream population genetic analyses. The outgroup-f_3 statistics analyses show that the Yushu0.5k is most closely related to present-day Tibetans from the southeastern Tibetan Plateau and present-day Qiang populations from Sichuan province (Fig. S3B). Yushu2.8k shows a similar pattern (Fig. S3A), but lower genetic similarity to them overall relative to Yushu0.5k. The archaeological sites and specimens are described below.

Pukagongma (n = 5)

The Pukagongma site is located in Zhidoi County, Yushu Prefecture, Qinghai, in the northern region of the Tibetan Plateau. The elevation of this site is 4,177 masl. Excavation at this site revealed a large number of stone coffins, and burial objects included bronze wares, pottery, animal bones and stone artifacts. The site was estimated to date to 2,720-2,426 BP (*76*). We generated genomic data for five specimens: CSP133, CSP134, CSP135, CSP136 and CSP137. Radiocarbon dates were generated for three specimens: 2997-2785 cal BP (2800±35 BP, CSP136), 2861-2760 cal BP (2720±25 BP, CSP133), and 2869-2755 cal BP (2710±35 BP, CSP135). All sampled specimens showed no relationship to each other in a familial relationship analysis. They were clustered as the “Yushu2.8k” group.

Galacun (n = 1)

The Galacun site is a temple site located in Galacun Village from the Yushu prefecture in Qinghai Province. It is estimated to date to the Ming Dynasty (1,368-1,644 AD) (*77*). The elevation of this site is 4,180 masl. We included one specimen, C514, whom we directly dated to 553-511 cal BP, consistent with the archaeological context.

Nagqu Prefecture, Tibet

We analyzed eight human samples from five sites (Ousui, Butaxiongqu, Gangre, Ounie, and Chaxiutang) from the Nagqu prefecture in the Tibet Autonomous Region. We retained seven unrelated individuals, who are clustered into five genetic groups, i.e. Nagqu2.7k, Nagqu2.5k, Nagqu1.6k, Nagqu1.4k, and Nagqu1.1k, for downstream population genetic analyses. Outgroup-f_3 statistics show that Nagqu2.7k and Nagqu2.5k are most closely related to present-day Tibetan populations from the southeastern Tibetan Plateau, and Nagqu1.6k, Nagqu1.4k, and Nagqu1.1k are most closely related to present-day Tibetan populations from the southeastern Tibetan Plateau (Fig. S4). The archaeological sites and specimens are described below.

Ousui (n = 1)

The Ousui cemetery, with an elevation of around 3,900 masl, is located in Lejiakucun Village, Biru county from Nagqu prefecture. We sampled C3991, for which we also generated a radiocarbon date of 2767-2709 cal BP (2600±30 BP). We refer to the individual represented by this specimen as Nagqu2.7k.

Butaxiongqu (n = 1)

The Butaxiongqu site is located in the Nagqu prefecture of the Tibet Autonomous Region. The elevation of this site is 4,623 masl. Stone coffins found at this site are dated to 2,460±30 BP (uncalibrated) using carbon-14 dating (*78*). Remains from animals including dogs, sheep, and horses were also found (*78*). We included one specimen, CSP144, who

was directly radiocarbon dated to 2683-2352 cal BP (2415±25 BP). This individual is designated as Nagqu2.4k.

Gangre (n = 1)

The Gangre cemetery, with an elevation of 4,740 masl, is located in Nima county from the Nagqu prefecture. Based on pottery and other artifacts found in stone tombs, the site is presumed to represent a Tubo or pre-Tubo culture (*79*). We included specimen C5085, for which we also generated a radiocarbon date range of 1738-1590 cal BP (1780±30 BP). This individual is designated as Nagqu1.6k.

Ounie (n = 4)

The Ounie cemetery is located in Baingoin county from the Nagqu prefecture (*80*). The average elevation is above 4,450 masl. The cemetery is famous for its sand pits and stone coffins, and is estimated to date to the early 7th-8th centuries, around the Sui or Tang dynasties. We included four specimens: C5172 and C3992 from Grave M2, C3993 from Grave M4, and C5173 from Grave M5, among which C5172 and C3993 were directly radiocarbon dated to 1520-1363 cal BP (1550±30 BP) and 1359-1290 cal BP (1420±30 BP), respectively.

In a familial analysis, specimens C5172 and C3992 were identified as deriving from the same individual (Table S4). We thus merged genomic data from these two individuals and refer to the merged set as individual C5172_C3992. The remaining two individuals did not show a familial genetic relationship. Collectively, these three individuals were clustered as the “Nagqu1.4k” group.

Chaxiutang (n = 1)

The Chaxiutang site is a ritual site also from Nagqu. It is estimated to be from the Tubo Period (618-842 AD) (*81*). The elevation of this site is 4,589 masl. We obtained CSP132 from this site and directly radiocarbon dated the specimen to 1179-963 cal BP (1170±40 BP). This individual is designated as Nagqu1.1k.

Chamdo Prefecture, Tibet

We analyzed four human samples from two sites (Redilong and Xiaoenda) from the Chamdo prefecture in the Tibet Autonomous Region. We retained four unrelated individuals, who are clustered into three groups, i.e. Chamdo2.8k_1, Chamdo2.8k_2, and Chamdo2.7k, for downstream population genetic analyses. Outgroup-f_3 statistics show that they are most closely related to present-day Tibetan and Qiang populations from the same or nearby regions (Fig. S5). The archaeological sites and specimens are described below.

Redilong (n = 2)

The Redilong site is located in the Chamdo prefecture of the Tibet Autonomous Region. The site is estimated to date to ~2,000 BP based on archaeological remains (*82*). The elevation of this site is 3,250 masl. Specimens CSP141 and CSP142 were included in this study, and CSP142 was directly radiocarbon dated to: 2850-2757 cal BP (2700±25 BP). The two individuals showed an asymmetric relationship to two tested reference individuals (Table S6), so they were assigned two independent genetic groups: Chamdo2.8k_1 and Chamdo2.8k_2.

Xiaoenda (n = 2)

The Xiaoenda site is located in the Chamdo prefecture of the Tibet Autonomous Region. The elevation of this site is 3,100 masl. It is a Neolithic site, and stone coffins were found at this site (*83, 84*). Two specimens, C1036 and C1037, were included in this study. Both

specimens were radiocarbon dated, to 2842-2736 cal BP (2645±25 BP) and 2722-2472 cal BP (2490±25 BP), respectively. In a familial analysis, the two individuals showed no familial relationship to each other, indicating that they were unrelated. They were clustered as the “Chamdo2.7k” group.

Nyingchi Prefecture, Tibet

We analyzed seven humans from three sites, i.e. Agangrong, Kangyu, and Gutong from this prefecture in the Tibet Autonomous Region. We retained six unrelated individuals, who are clustered into four groups (Nyingchi2k, Nyingchi2k_o, Nyingchi0.8k, Nyingchi0.1k) for downstream population genetic analyses. Outgroup-f_3 statistics show that Nyingchi2k, Nyingchi2k_o, and Nyingchi0.8k are most genetically similar to present-day Tibetan and Qiang populations on or nearby the plateau, Nyingchi0.1k is most closely related to present-day Han populations (Fig. S6). The archaeological sites and specimens are described below.

Agangrong (n = 5)

The Agangrong site is from Bome county in the Nyingchi prefecture from the southern Tibetan Plateau, with an elevation of around 3,000 masl. The estimated date is 2700-1800BP (*85*) based on the archeological context of 13 excavated tombs from the site. We obtained five specimens from the site: C3443, C3444, C3445, C3447 and C5186. We also generated radiocarbon dates for three specimens: 1992-1830 cal BP (1980±30 BP, C3444), 2100-1889 cal BP (2040±30 BP, C3447), and 2096-1882 cal BP (2030±30 BP, C3445).

In a familial analysis (Table S4), specimens C3447 and C5186 were identified as deriving from the same individual. They are also from the same grave (Table S2). Thus, we merged their genomic data and refer to the merged set as individual C3447_C5186. Among the three remaining specimens, no familial genetic relationships were found.

C3444 produced an asymmetric signal (Table S6) when compared with the other two individuals, and was assigned to a distinct “Nyingchi2k_o” group. The remaining two individuals were clustered as the “Nyingchi2k” group.

Kangyu (n = 1)

The Kangyu cemetery is from Bome county in the Nyingchi prefecture. Human specimens were excavated in 2019 from this site. We included one specimen, C5190, and generated a radiocarbon date of 909-732 cal BP (900±30 BP). This individual is designated as Nyingchi0.8k.

Gutong (n = 1)

Gutong is a cave site from Bome County in the Nyingchi prefecture. Human specimens were discovered in 2019. We included one specimen, C5169, for whom we generated the radiocarbon date range of 275-8 cal BP (130±30BP BP). This individual is designated as Nyingchi0.1k.

Shannan Prefecture, Tibet

We analyzed seven human samples from four sites (Tingcun, Yusa, Jiesang, Dama) from the Shannan prefecture in the Tibet Autonomous Region. We retained six unrelated individuals, who are clustered into three groups, i.e., Shannan2.2k_1, Shannan2.2k_2, and Shannan_1.3k, for downstream population genetic analyses. Outgroup-f_3 statistics show that they are most closely related to present-day Tibetan populations on the same or nearby regions (Fig. S7). The archaeological sites and specimens are described below.

Tingcun (n = 1)

The Tingcun cemetery is in Lhozhag county from the Shannan prefecture, with an average elevation of 3,800 masl. The site was first excavated in 2017, and a large amount of pottery was unearthed from this site (*86*). We included one specimen, for which we also generated radiocarbon dates: 3063-2873 cal BP (2850±30 BP). This individual is designated as Shannan3k.

Jiesang (n = 3)

The Jiesang cemetery is from the Naidong district in the Shannan prefecture, with an elevation of around 3,700 masl. Coffin chambers, oriented to the west with trapezoidal or rectangular planes, are found at this site. Stone coffins are found inside the chambers but burial objects are scarce (*87*). We included three samples (C3450, C3455, C3456) and directly radiocarbon dated the C3455 and C3456 specimens. They were dated to 2313-2099 cal BP (2180±30 BP) and 2326-2144 cal BP (2210±30 BP), respectively. After quality control, only two specimens were retained for genetic analysis, C3455 and C3456. They showed no familial genetic relationship to each other, and were clustered together as the Shannan2.2k_1 group.

Yusa (n = 1)

The Yusa cemetery is located in the Naidong district of the Shannan prefecture. We collected one specimen and directly radiocarbon dated the specimen, C5145, to 2341-2153 cal BP (2250±30BP BP). This individual is designated as Shannan2.2k_2.

Dama (n = 2)

The Dama cemetery is in the Shannan prefecture, and it was excavated in 2018. The site is estimated to date back to the East Han dynasty based on burial excavations (*88*). We sampled two specimens, C5187 and C5189, and generated radiocarbon dates for each of 1400-1307 cal BP (1480±30 BP) and 1290-1176 cal BP (1300±30 BP), respectively. In

a familial analysis, the two individuals do not show a familial genetic relationship, suggesting that they are unrelated to each other. They were clustered as the “Shannan1.3k” group.

Lhasa Prefecture, Tibet

We analyzed five humans from four sites (Lajue, Gachong, Jawutang, Shigou) from the Lhasa prefecture in the Tibet Autonomous Region. We retained three unrelated individuals, who are clustered into three groups, i.e., Lhasa1.1k, Lhasa1k, and Lhasa0.7k, for downstream population genetic analyses. Outgroup-f_3 statistics show that they are most closely related to present-day Tibetan populations from the same or nearby regions (Fig. S8). The archaeological sites and specimens are described below.

Lajue (n = 2)

Lajue cemetery is in Lhasa, the capital of the Tibet Autonomous Region, with an average elevation of 3,650 masl. The date of this site is estimated to be around the Tubo time period (*81*). We sampled two specimens, C5077 and C1357, and generated radiocarbon dates for C1357 of 1178-977 cal BP (1170±30 BP). The C5077 individual was excluded from downstream analyses due to high contamination. The C1357 individual was designated as “Lhasa1.1k”.

Gachong (n = 1)

The Gachong cemetery was excavated in 2018 in Duilongdeqing county from the Lhasa prefecture. We included one specimen, C5144, from this site and directly radiocarbon dated C5144 to 1055-916 cal BP (1050±30BP). This individual was designated as Lhasa1k.

Jawutang (n = 1)

Jawutang site is located in the Semai village from the Lhasa prefecture. The popular tomb structure found in Jawutang cemetery is rarely seen in Lhasa Valley or Yarlung Tsangpo River Valley (*89*). We included one specimen, C3441, from this site and directly dated C3441 to 1291-1174 cal BP (1290±30BP). Very little genomic data was generated for this specimen, and C3441 was not included in downstream genetic analyses.

Shigou (n = 1)

The Shigou site is located in the Lhasa prefecture, and it is estimated that the site dates back to no earlier than AD 1200 based on Tibetan texts excavated along with the human samples (*90*) . Specimen CSP130 was sampled from this site. This individual is designated as Lhasa0.7k.

Eastern part of the Shigatse Prefecture, Tibet

The eastern and western parts of Shigatse prefecture in the Tibet Autonomous Region were referred to differently throughout this study, with western Shigatse referred to as the southwestern Tibetan Plateau while the eastern Tibetan Plateau was combined with Lhasa and Shannan Prefecture to be collectively referred to as southern Tibetan Plateau. We analyzed 24 humans from four sites, i.e. Nudagang, Rangjun, Longsangquduo and Latuotanggu of eastern Shigatse. We retained 20 unrelated individuals, who are clustered into four groups, i.e., Shigatse2.1k, Shigatse1.2k, Shigatse0.9k and Shigatse0.7k, for downstream population genetic analyses. Outgroup-f_3 statistics show that they are most closely related to present-day Tibetan populations from the same or nearby regions, including Lhasa, Shannan, Shigatse, Nagqu and Nyingchi (Fig. S9). The archaeological sites and specimens are described below.

Nudagang (n=3)

The Nudagang cemetery is located in the Rinbung County from the Shigatse prefecture. The site was excavated in 2019, and we sampled three specimens for which we also generated radiocarbon dates: 2302-2003 cal BP (2150±30 BP, C5146), 2295-2000 cal BP (2130±30BP BP, C5148), and 2336-2149 cal BP (2230±30BP BP, C5149). In a familial analysis, the three individuals were unrelated to each other. They were clustered as the “Shigatse2.1k” group.

Rangjun (n = 2)

The Rangjun cemetery is located in Rinbung county from the Shigatse prefecture. This site has archaeological similarities to the Xiaoenda cemetery in Chamdo (*91*). We included two specimens from this site, C5150 and C5151. C5150 and C5151 were both directly radiocarbon dated to 1291-1174 cal BP (1290±30BP BP) and 1277-1073 cal BP (1250±30 BP), respectively. C5151 was not included in downstream analyses, as too little genomic data were obtained to evaluate levels of modern human contamination. The individual C5150 was named “Shigatse1.2k” for genetic analysis.

Longsangquduo (n = 14)

The Longsangquduo cemetery was discovered in 2018 in Namling county from the Shigatse prefecture. We sampled 14 specimens from this site: C5152, C5153, C5154, C5155, C5156, C5157, C5158, C5159, C5160, C5161, C5162, C5163, C5164, and C5165. Four specimens were radiocarbon dated to 922-779 cal BP (940±30 BP, C5159), 960-797 cal BP (1010±30 BP, C5165), 959-797 cal BP (1000±30 BP, C5157), and 973-799 cal BP (1020±30 BP, C5153).

In a familial analysis (Table S4), C5163 and C5164 were identified as possessing a 1-degree familial relationship, which corresponds most parsimoniously to a mother and son or a father and daughter. We retained C5164 for downstream analyses, as C5163 had lower levels of genomic data. C5164 and the remaining 12 specimens were collectively clustered as “Shigatse0.9k”.

Latuotanggu (n = 5)

The Latuotanggu site is in Laluo village from the Sa'gya county in the Shigatse prefecture. We included five specimens from this site: C3427, C5171, C3425, C3428, and C3426. Radiocarbon dates were generated for four of them: 772-675 cal BP (810±30BP BP, C5171), 675-560 cal BP (680±30BP BP, C3425), 683-563 cal BP (700±30BP BP, C3428), and 909-732 cal BP (900±30BP BP, C3426).

In a familial analysis (Table S4), C3426 and C3428, both from Grave M5, were identified to possess a 1-degree familial relationship, which corresponds most parsimoniously to a father and son. We further identified that C5171 shows a 1-degree familial relationship with C3426 and a 2-degree familial relationship with C3428 (Table S4). Due to the extended familial genetic relationships between these three specimens, we retained only the specimen with the highest levels of genomic data, C5171. We thus removed C3426 and C3428 from further downstream analyses. C5171 and the two individuals were clustered as "Shigatse0.7k".

Western part of the Shigatse prefecture, Tibet

We analyzed five humans from three sites (Sila, Sding Chung, Zhangcun) from the western part of the Shigatse prefecture. We retained five unrelated individuals who are clustered into four genetic groups: Shigatse2.6k, Shigatse1.9k, Shigatse1.5k_1 and Shigatse1.5k_2, for downstream population genetic analyses. Outgroup-f_3 statistics show that Shigatse2.6k, Shigatse1.9k, and Shigatse1.5k_2 are most closely related to present-day Tibetan and Sherpa populations on the plateau (Fig. S10), while Shigatse1.5k_1 is most closely related to Han populations. The archaeological sites and specimens are described below.

Sila (n = 2)

The Sila site is a stone chamber cemetery, which is located in the western regions of the Shigatse prefecture. The elevation of this region is ~4,000 masl. We sampled two specimens, C403 and C404, and directly generated radiocarbon dates for these two specimens, 2745-2496 cal BP (2540±30 BP) and 2721-2425 cal BP (2480±30 BP), respectively. In a familial analysis, the two individuals showed no familial genetic relationship to each other, suggesting that they were unrelated. We clustered them as the "Shigatse2.6k" group.

Sding Chung (n = 1)

The Sding Chung site is a cave burial in Sding Chung county located on the western edge of the Shigatse prefecture, with an elevation of 5,000 masl. We sampled one specimen, C5417, for whom we also generated a radiocarbon date range of 1928-1745 cal BP (1930±30 BP). The genetic group "Shigatse1.9k" was assigned to the individual.

Zhangcun (n = 2)

The Zhangcun cemetery is in Gyirong city in the Shigatse prefecture, with an elevation of 2,793 masl. The site was excavated in 2019 and two human specimens, C5185 and C5184, were excavated. We sampled both specimens and generated radiocarbon dates for C5184 of 1520-1363 cal BP (1550±30 BP). The two individuals showed an asymmetric genetic relationship to two tested reference individuals (Table S6). Thus, we assigned them to separate genetic groups, Shigatse1.5k_1 and Shigatse1.5k_2.

Ngari Prefecture, Tibet

We analyzed ten humans from two sites (Piyangjiweng and Gelintang) from the Ngari prefecture in the Tibet Autonomous Region. We retained seven unrelated individuals, who were clustered into four groups: Ngari2.3k, Ngari2.3k_o1, Ngari2.3k_o2, and Ngari0.1k,

for downstream population genetic analyses. Outgroup-f_3 statistics show that they are most closely related to present-day Tibetan and Sherpa populations on the plateau (Fig. S11). The archaeological sites and specimens are described below.

Piyangjiweng (n = 9)

The Piyangjiweng cemetery is found in the Ngari prefecture (*92*) and is estimated to date back to around the 1st century AD, corresponding to the Han and Jin dynasties. An archaeological survey showed evidence of secondary burial at this site. We sampled nine specimens from different graves: C4563, C4564, C4565, C456, C4567, C4568, C4569, C4570 and C4571. We directly radiocarbon dated C4564 to 2344-2155 cal BP (2260±30 BP).

In a familial analysis (Table S4), three specimens (C4565, C4566 and C4570) were identified as deriving from the same individual, but they are from different graves, possibly due to a secondary burial (Table S2). We retained only the specimen with the most genomic data available for downstream analysis, i.e. C4566. For the remaining six specimens, none were found to share a familial genetic relationship. We found that four individuals (C4564, C4567, C4569, and C4571) clustered together, designated as "Ngari2.3k". The remaining two were outliers that did not cluster with each other in an f_4-analysis (Table S6), with C4566 designated as "Ngari2.3k_o1" and C4563 designated as "Ngari2.3k_o2".

Gelintang (n = 1)

The Gelintang cemetery is in the Ngari prefecture from the Tibet Autonomous Region, with an average elevation of 4,500 masl. Several other cemeteries in the vicinity share many common features (*93*): for example, bronze swords bearing similarities to those found in the northern steppe were excavated from Gelintang and other cemeteries,

suggesting a connection to steppe cultures (*94*). We sampled the specimen CSP147, who is dated to 262-27 cal BP (110±20 BP) (*95*).

Principle Component Analysis (PCA)

In Fig. 2 and Fig. S12, we showed a PCA constructed with diverse Asian populations, including from East Asia, Central Asia, South Asia and Southeast Asia. To further investigate the relationship between the ancient individuals and different East Asian groups, we also performed PCA with a present-day panel including only East and Southeast Asian populations. On this PCA (Fig. S13), all the projected ancient Tibetan Plateau individuals overlap with or are close to present-day Tibetan and Qiang populations. Ancient individuals on the northeastern Tibetan Plateau shift slightly towards present-day Mongolians and Siberians (Fig. S13). Individuals from the western Tibetan Plateau are shifted towards present-day northern East Asians.

ADMIXTURE analysis

We performed an unsupervised structure analysis with ADMIXTURE (version 1.3.0) to examine the genetic relationship between ancient individuals of the Tibetan Plateau and worldwide present-day and ancient humans. For present-day populations, we included diverse worldwide populations from the 929 Diverse Genomes Project (*96*) (Fig. S14), as well as present-day Han and Tibetans (*6*) that were sampled from across China, divided by province (Han) or prefecture (Tibetan). For ancient humans, we included individuals spanning from the Neolithic to historical times from regions around the Tibetan Plateau, such as the Eastern Eurasian Steppe (*62, 63*), Central and South Asia (*97*), and East and Southeast Asia (*7, 36, 65, 98*). ADMIXTURE analyses were performed with K varying from 2 to 14, and for each K, 10 random-seed replicates were run (Fig. S14). For each K, the admixture output with the lowest CV value is plotted (Fig. S15). The result for K = 11 is presented in the main text which has the lowest CV values (Table S5).

Genetic clustering

As described in the Methods section, we tested f_4-statistics in the form of f_4 (X1, X2, Z, Mbuti) where X1 and X2 are individuals from the same test site and Z are individuals from a reference panel (see Methods). In most cases, we found that individuals from the same archaeological site show a symmetric relationship to reference panel populations with the exception of groups from the archaeological sites of Zongri, Piyangjiweng, Zhangcun, Longsangquduo, Agangrong and Redilong, which are described in detail below (Table S6).

At the Zongri site, multiple asymmetric signals were observed (Table S6), indicating high genetic heterogeneity across the 1,000-year period sampled. Many signals were driven by the oldest individual (C4783_C202) and the C4781 individual. In a PCA, all the Zongri individuals cluster together excepting the oldest individual. In outgroup f_3 analyses, all Zongri individuals except C4783_C202 and C4776 cluster together. Thus, we assigned a distinct genetic group, Zongri5.1k, to C4783_C202. We also assigned three other individuals different group names based on their dating, with C050 assigned to Zongri4.7k, and C051 and CSP054 assigned to Zongri4.1k. The remaining individuals were grouped as Zongri4.5k. C4781 and C4776 are outliers, whom we denoted as Zongri4.5k_o1 and Zongri4.5k_o2, respectively.

Notably, in the f_4-statistic tests (Table S6), we found that the C4783_C202 individual (Zongri5.1k) shows a connection with a 45,000-year-old Siberian (UstIshim), a pattern not found in other Zongri individuals. We further investigated this connection by comparing Zongri5.1k with other Neolithic East Asians and with f_4-statistics using both Mbuti and Chimp as outgroups. We confirmed this signal (Table S7) and found this signal is particularly strong when compared with other northern Neolithic East Asians.

At the Piyangjiweng site, asymmetric signals among the six individuals were mostly driven by the C4566 and C4563 individuals (Table S6). Comparison to the reference panel

showed that the C4563 individual shares excess similarity to the Afanasievo from the Eastern Eurasian steppe. In comparisons with C4566, all other Piyangjiweng individuals share more genetic similarity with both the 40,000-year-old Tianyuan individual and the Middle Neolithic East Asians from the Yellow River region (YR_MN), than with C4566. Given the deep divergence between Tianyuan and YR_MN, the results support influence from an unknown deeply diverged ancestry. With the main group "Ngari2.3k", we used distinct names for C4566 and C4563 of "Ngari2.3k_o1" and "Ngari2.3k_o2", respectively.

At the Zhangcun site, the two individuals C5185 and C5184 do not cluster in a PCA (Fig. 1F), and C5185 shows excess genetic similarity with Chokhopani and Luozhating in f_4-statistics (Table S6). Furthermore, they show different genetic profiles in an *ADMIXTURE* analysis (Fig. S14). Therefore, we denoted them separately as "Shigatse1.5k_1" and "Shigatse1.5k_2".

At the Longsangquduo site, two marginal asymmetric signals were found (Table S6). However, no consistent pattern can be determined from PCA, ADMIXTURE, or outgroup f_3 analyses to suggest differentiation across ancient individuals from this site. We therefore kept all individuals in a single "Shigatse0.9k" group.

At the Agangrong site, the C3447_C5186 and C3445 individuals share excess alleles with 19,000 and 11,000-year-old individuals from northern (AR19K) and southern (Qihe3) China relative to C3444 (Table S6). This suggests that C3444 does not cluster with the other two individuals, and we therefore identify this individual as an outlier, denoted as 'Nyingchi2k_o'. We clustered the other two as "Nyingchi2k".

At the Redilong site, CSP142 shows excess genetic similarity to the Pukagongma and Qihe3 individuals (*99*) relative to CSP141 (Table S6). We thus denote them separately as "Chamdo2.8k_1" and "Chamdo2.8k_2".

TreeMix analysis

To investigate the genetic relationships among populations from the Tibetan Plateau, we performed TreeMix analysis. We included populations that are relevant and are representative of different modern human lineages, including Mbuti, UstIshim and Tianyuan. We included Gonur1_BA to account for the West Eurasian ancestry found in Ngari samples. We also densely sampled from East Asian populations to capture different human lineages. From the Tibetan Plateau, we included populations that are representative of different clusters.

When allowing migration edges to vary from 0 to 3, we found that all the ancient Tibetan Plateau populations consistently clustered with East Asian populations (Fig. S19). With migration edges varying from 0 to 2, ancient Tibetan Plateau populations consistently formed a clade with ancient northern East Asians (Fig. S19). We showed a model with one migration edge to account for discernible shared ancestry between Gonur1_BA and Ngari2.3k (Fig. 2A). We also obtained statistical support from each clade in this model by performing 1,000 bootstraps.

For the Treemix analysis, there is 42.2% support for the plateau clade. However, this is primarily because Zongri5.1k, Yushu2.8k, and to a lesser extent Ngari2.3k are not as consistently grouping solely with the other ancient plateau groups. In the 1,000 bootstrap replicates, 43.5% of trees showed Zongri5.1k and/or Yushu2.8k as outgroup to <10,000-year-old northern East Asians and plateau populations, AR19k, other northern East Asians and plateau populations, or all plateau and lowland East Asians. Thus, the support for other plateau populations forming a clade is actually 85.7%. Allowing Ngari2.3k to also fall outside of the plateau-related clade in addition to Zongri5.1k and Yushu2.8k increase support for a plateau-related clade to 94.4%. Given that these three sets are not grouping exclusively with another East Asian phylogeny, we expect that the low support for the plateau clade is influenced by Treemix not capturing the unsampled ancestry well, as there are no representative source populations to include in the analysis.

Outgroup-f3 analysis

We performed outgroup-f_3 statistics, f_3 (X, Y; Mbuti), where for X, we included all Early Ancient Tibetans, and for Y, we included all present-day and ancient individuals from Asia, particularly East Asia. This analysis is useful in identifying who shares high genetic similarity and inferring past population migration. We also note that the interpretation of the analysis depends on the availability of samples tested as Y.

We examined the 2200-1900 BP individuals from the southern and southwestern Tibetan Plateau, and we found the 3,400-2,200-year-old individuals from the same region shares the most genetic similarity (Fig. S21), which is suggestive of local genetic continuity. For 2,000-year-old individuals from the Nyingchi prefecture in the southeastern Tibetan Plateau, the ancient individuals from the southern and southwestern Tibetan Plateau share the highest genetic similarity (Fig. S22). The 1,600-1,100-year-old individuals from the Nagqu prefecture also share the most genetic similarity to ancient individuals from the southern and southwestern Tibetan Plateau, rather than the local populations that preceded them (Fig. S23).

Admixture-f3 analysis

To investigate the admixture history associated with ancient Tibetan Plateau populations, we performed f_3-statistics of the form f_3 (Ref1, Ref2; Test). When the value is negative, that means the Test population is admixed from source populations related to Ref1 and Ref2. We exhaustively searched admixture signals for Zongri4.5k, Zongri4.1k, Shigatse2.6k, Yushu2.8k, Chamdo2.7k, Ngari2.3k, Shannan2.2k_1, Shigatse2.1k, Nyingchi2k, Nagqu1.4k, Shannan1.3k, Shigatse0.9k and Shigatse0.7k (Test groups). Ref1 and Ref2 consisted of pairs of relevant ancient populations from East, Southeast, and Central Asia, as well as one the plateau at Ref1 and Ref2. No significantly negative pairs were found for Chamdo2.7k, Nagqu1.4k, Shannan2.2k_1, Shigatse2.1k, Shigatse2.6k, Yushu2.8k and Zongri4.1k. We found significantly negative pairs for

Zongri4.5k, Ngari2.3k, Nyingchi2k, Shannan1.3k, Shigatse0.9k and Shigatse0.7k (Table S10).

For Zongri4.5k, significantly negative f_3 values occurred when one source was a local Zongri group, and another was an ancient East or Southeast Asian group. For Ngari2.3k, significantly negative values occurred when one source is related to ancient Tibetan Plateau individuals, and the other source is related to populations harboring West Eurasian ancestry. For Nyingchi2k, significantly negative f_3 values show that one source is related to ancient Tibetan Plateau individuals, and the other source is related to Early or Middle Neolithic East Asians. For Shannan1.3k, Shigatse0.9k and Shigatse0.7k, they share one of the following patterns: (1) has one source related to ancient Tibetan Plateau individuals, and another other source related to diverse populations from East, Southeast, and Central Asia.

We also calculated admixture-f_3 statistics for present-day populations on the Tibetan Plateau, including Tibetan_Chamdo, Tibetan_Nyingchi, Tibetan_Shannna, Tibetan_Lhasa, Tibetan_Nagqu, Tibetan_Shigatse and Sherpa_Shigatse (Table S11). No significant pairs were found for Tibetan_Nyingchi, while for other populations, one source is related to ancient populations on the Tibetan Plateau, and the other source is related to population out of the plateau. Particularly, for Tibetans on the southern and southwestern Tibetan Plateau, including Tibetan_Shannan, Tibetan_Lhasa, and Tibetan_Shigatse, the other source is widely related to populations from East Asia, Southeast Asia and Eastern Eurasian Steppe.

qpAdm modeling

i. QpAdm modeling of Tibetan ancestry in Early Ancient Tibetans

We adopted the "rotating scheme" to perform *qpAdm* modeling (*14, 16, 30, 67, 100, 101*) ancestry in ancient plateau populations older than 2,500 BP. In this design, N populations that are related to the ancestry sources of target populations were assembled as a

RightAll set. For each target population, we iteratively fit one, two and three-source mixture models. When testing models with n (n = 1, 2, 3) sources for each target, we randomly drew n populations from the RightAll set as potential sources, and all other populations (N-n) in RightAll were retained as an outgroup population, i.e. the right populations for a *qpAdm* run. For each n-source model, we tested all "N choose n" possible models. We started by testing all one-source models, and we retained only feasible models, defined as those with a p-value greater than 0.05, and where the estimated mixture proportions up to +/- 2 standard errors are within the (0,1) range. If feasible models are found for the one-source model, then two- or three-source models are not examined for that target population. If all one-source models are rejected, we then start testing two-way source models and so on. This modeling strategy is powerful in distinguishing between plausible and implausible models, allowing identification of the best-fitting models from a panel of source populations.

To obtain a distal model for the ancestries in the ancient Tibetan Plateau populations (Table S14), we included diverse ancient and present-day populations to the RightAll sets, particularly adding a variety of ancient East Asian individuals of diverse ancestries sampled to date. We considered three lines of criteria. First, we included populations that are temporally and geographically close to the target population we studied. Second, we included populations that show relevance to the target population in the exploratory PCA and *ADMIXTURE* analysis. Thrid, we restricted to groups with >600,000 SNPs available to maximize the statistical power of the analysis.

We first included populations that are representative of different lineages of modern humans:

Mota: a 4,500-year-old Ethiopian;

UstIshim: a 45,000-year-old Siberian that is basal to Eurasians;

Onge: a present-day indigenous population from the Andaman Islands that represents a divergent lineage from Asians;

Tianyuan: a 40,000-year-old East Asian from Beijing, China;

Malta1: a 24,000-year-old Siberian individual that is more related to present-day western Eurasians and Native American than East Asians;
Clovis: an ancient American individual related to the Clovis complex;
CHG: Caucus Hunter gatherers, including two individuals, Satsurblia and Kotias;

In *ADMIXTURE* and PCA, ancient Tibetan Plateau populations are most closely related to East Asians, we therefore include a diverse set of ancient East Asians that are representative of different lineages:

Shamanka_EN: Early Neolithic population of East Asian ancestry found near the Lake Baikal region
Yumin: 8000-year-old individual from Northern China
Shandong_EN: Early Neolithic individuals from Shandong, China. It includes Bianbian, Boshan and Xiaogao
Fujian_EN: Early Neolithic individuals from Fujian, China. It includes Liangdao2 and Qihe, Liangdao1 is not included since it harbors ancestry related to ancient northern East Asians.
YR_MN: Middle Neolithic population from the lower reaches of the Yellow River
Atayal: present-day aboriginal population from the island of Taiwan

Ancient Tibetan Plateau populations also harbor ancestry related to Neolithic and Bronze-age Central Asians. We therefore included:

Ganj_Dareh_N: the Neolithic Iranian individuals;
Gonur1_BA: the bronze-age central Asian individual from Turkmenistan.

In summary, 15 populations (R15) were included for distal modeling of Tibetan ancestry:
R15: *Mota*, *UstIshim*, *Tianyuan*, *Malta1*, *Clovis*, *CHG*, *Ganj_Dareh_N*, *Gonur1_BA*, *Shamanka_EN*, *Yumin*, *Shandong_EN*, *Fujian_EN*, *YR_MN*, *Atayal* and *Onge*

To obtain more proximal models, we added Zongri5.1k to the rotating outgroup to model the gene flow in Zongri region ("R15 + Zongri5.1k", Table S15). We also tested two

additional rotating outgroups: “R15 + Lajia4k + Zongri5.1k” and “R15 + Lajia4k + Lubrak”, where Lajia4k is late Neolithic populations from the upper reaches of Yellow River, and Lubrak is one the earliest plateau populations from the Himalayan arc. These two additional outgroup sets were used to analyze proximal models for Early Ancient Tibetans younger than 3,000 BP.

ii. Modeling of ancestries in ancient plateau populations after 2800 BP

To understand the relationship between plateau populations after 2800 BP (target population) and the preceding ancient plateau populations, we model the target populations as a mixture of five possible ancestries: South/Central Asian related ancestry represented by Gonur1_BA; East Asian lowland ancestry represented by Dacaozi1.9k from Upper Yellow River. Three different plateau ancestries represented by Chamdo2.8k_1, Yushu2.8k and Lubrak. Ten populations including Mota, UstIshim, Tianyuan, Clovis, Ganj_Dareh_N, Yumin, Tanshishan, Lajia4k, Suila and Zongri5.1k were used as outgroup populations. Using *qpWave*, we first show that the five ancestries in the 5 representative source sets can be discerned by the outgroup population as f4rank = 4 was rejected at $P = 0.015$. For each target population, models with 1-, 2- and 3-way mixtures were successively tested until the best models were found for each target population (Tables S17). Criteria for a feasible model is the same as described above. When fitting models for present-day populations, we masked CpG sites to avoid potential bias introduced through DNA damage.

<u>Estimating archaic related ancestry</u>

To investigate archaic ancestry in ancient Tibetan Plateau individuals, we first tested for excess shared alleles with the Altai Neanderthal and the Denisovan using f_4-statistics (Table S18). The results show that archaic humans share a similar number of alleles with all ancient individuals and present-day East Asians, but they share more alleles with Papuans than ancient individuals. We further estimate the proportion of archaic ancestry

in each individual by inferring ancestry fragments with Admixfrog (Table S20), and we found that the estimated proportion is comparable to that found in present-day East Asians. This indicates that all of the ancient plateau individuals harbor similar amount of archaic ancestry as that in present-day East Asians, and no evidence of elevated archaic ancestry can be found.

Testing the longitudinal cline on populations from the Tibetan Plateau

In Jeong et al. (*22*), the authors described a longitudinal cline in present-day populations on the Tibetan Plateau, with the eastern populations tend to share closer genetic affinity with lowland East Asians. The observation is underlain by the correlation between longitudinal differences and differences in allele-sharing with lowland East Asians, which was measured by D-statistics in the form of D (Tibetan 1, Tibetan 2; Han, African). Here we test to what extent this cline can be found in ancient Tibetan samples, we calculated statistics in the form of f4(Tibetan 1, Tibetan 2; lowland East Asian, African). For lowland East Asian, we tested representative East Asians from different locations and different time periods.

We found a significant correlation (unadjusted P = 0.02-0.03, Mantel test) among present-day populations (Fig. S26), supporting previous observations (*22*). Moreover, the correlation signals are consistent when testing different lowland East Asian populations. We then test this signal in ancient populations using different time transects. For each time transect, we only included populations that are of similar age to be comparable with each other. We also tried to maximize the geographic span of samples, and we eliminated outlier samples. For the ~2,800 BP transect, we included Yushu2.8k, Nagqu2.7k, Chamdo2.8k_1, Shigatse2.6k, Lubrak and Shannan3k (Fig. S27). For the ~2,000 BP transect, we included Shannan2.2k_1, Shigatse2.1k, Shigatse1.9k, Nyingchi2k and Mebrak (Fig. S28). For both transects, there is no correlation between the longitudinal difference and differences in allele-sharing with lowland East Asians (unadjusted P = 0.41-0.68 and P = 0.36-0.55, Mantel test). Ancient Tibetans from different longitudes

consistently show similar levels of genetic affinity with lowland East Asians. For the ~1500 BP transect, we included Samdzong, Shigatse1.5k_2, Nagqu1.4k, Shannan1.3k (Fig. S29). For ~1000 BP time transects, we included Nagqu1.1k, Lhasa1.1k, Lhasa1k, Shigatse0.9k and Nyingchi0.8k (Fig. S30). They show a trend of correlation, but no robust correlation signal can be observed (unadjusted P = 0.07-0.36 and P = 0.05-0.2, Mantel test). These results show that the longitudinal cline in Tibetan populations formed after 1,000 BP, consistent with our *qpAdm* modeling analysis showing that extensive gene flow from lowland East Asians can only be detected in the recent past few hundred years.

<u>Allele frequency and genotype at phenotypic SNPs</u>

We first retrieved the bases at each SNP for each individual from the sequence read data. Using the sequenced reads, we estimated the allele frequency at each SNP and performed genotype calling at the the *EPAS1* locus using the following equation:

Error rate estimate for ancient DNA

The error rate (ε) is an important parameter in estimating genotype and allele frequencies, and the error rate in ancient DNA is often high due to DNA damage. To obtain an empirical estimate of ε, we first estimated the error rate in genome-wide data by considering all the bases in reads supporting the third allele across all SNPs as sequencing errors. We found that the error rate estimates range from 0.0003-0.0027 (Table S3). We also took advantage of the high number of aggregated read information at the *EPAS1* locus to independently obtain an empirical error rate. We took all the ancient individuals that have more than ten read counts and were called as homozygotes (Table S21), treating the reads supporting the alternative allele as sequencing errors. In total, we found 26 errors among 4354 bases, suggesting an error rate of 0.006. To test the robustness of the maximum likelihood framework in estimating the allele frequency given the observed range of the error rate, we varied the parameter epsilon (ε) from 0.0001 to 0.01 to estimate the allele frequency of *EPAS1* in the Early Ancient Tibetans (Fig. S32). We find that the allele frequency estimates are robust to changes in the error rate. When

estimating the genotypes and population allele frequencies, we make conservative estimates by using an error rate of 0.005, which is close to the high end of the error rate estimate.

EPAS1

In the *EPAS1* gene region, twenty SNPs are highly differentiated between present-day Tibetans and other modern humans (*32*). These have been used to define an *EPAS1* haplotype common to present-day Tibetan populations. Because our data is low coverage, we cannot confidently assign genotypes at each SNP. Instead, we assumed that the 20 SNPs are in complete linkage since this haplotype was introgressed from an archaic human and high F_{ST} values were observed between Tibetans and other populations across these SNPs (*32*). Assuming this, we gathered available read information at these SNPs to infer the haplotype. These twenty SNPs are at least 270 bp apart, longer than the length of a single read. Thus, we aggregated the number of mapped reads across all twenty SNPs and calculated the frequency of the common Tibetan allele in each individual (Table S21). We used these data to determine the genotype of each individual: either homozygous for the adaptive Denisovan-like haplotype, homozygous for the non-adaptive haplotype found in most modern humans, or heterozygous.

To track the allele frequency change of *EPAS1* over time, we grouped individuals from the central, southeast, southern and southwest Tibetan Plateau into one of four time periods: 3,400-2,500 BP, 2,200-1,900 BP, 1,600-700 BP, and the present day. Of note, the population allele frequencies in present-day Tibetans were calculated using the genomic data of 33 present-day Tibetans from the Chamdo, Nyingchi, Lhasa, Shannan, Nagqu and Shigatse prefectures (*6*). Details can be found in Table S21.

When comparing the allele frequency between the Early Ancient and present-day Tibetans, we observed a substantial increase in allele frequency (δ = 0.50). To test whether the increase is due to natural selection, we compared the change in allele frequency for the Denisovan-like haplotype to changes in allele frequency for random

SNPs presumed to be primarily shaped by genetic drift. We constructed a null distribution of allele frequency change for SNPs that have a similar initial frequency as that of *EPAS1* in the 3,400-2,500 BP group (Fig. S31). To do this, we first estimated the allele frequency for each SNP in the 3,400-2,500 BP group using maximum likelihood methods described above (Eq. 3) and retained 65,296 SNPs with an allele frequency between 0.31-0.41, the frequency range determined for the Denisovan-like haplotype in the 3,400-2,500 BP group (f=0.0.36). We then calculated the allele frequency of these SNPs in present-day Tibetans. The change in allele frequency was obtained by taking the difference between ancient and present-day populations. We found the change in allele frequency at *EPAS1* is significant ($P = 0.0055$) under the null distribution (Fig. S31).

Other phenotypically relevant genes

We also explored the allelic patterns at several other genes related to appearance and diet, including *EDAR*, *HERC2*, *KITLG*, *MCM6*, *IRF4*, *SLC24A5*, *SLC45A2*, *TYR*, *OCA1*, *MC1R*, *ALDH2,* and *ADH1B*.

In the *EDAR* gene, the EDAR*V370A mutation in the rs3827760 SNP is found in high frequency in present-day East Asian populations (*102*) and is associated with thick hair and shovel teeth (*102-105*). The 40,000-year-old Tianyuan individual shows the ancestral allele for EDAR (*48*), but most ancient western East Asians are homozygous for the derived allele, including the 5,100-year-old Zongri individual (Table S23). The two 2,700-2,500-year-old individuals from Nagqu are both homozygous for the ancestral allele (Table S23).

In *HERC2*, rs12913832 is associated with blue eyes in humans (*106*). In *KITLG*, rs12821256 is associated with blonde hair (*107*). In *IRF4*, rs12203592 is associated with lighter eye and hair color (*108, 109*). In *SLC24A5* and *SLC45A2*, rs1426654 and rs16891982 are associated with light skin (*110, 111*). In *MC1R*, rs1805005 and rs1805006 are associated with light blonde and red hair (*112*). In *TYR*, rs1042602 and rs1393350 are associated with freckles, skin and eye color (*113*). The derived alleles of

these SNPs are primarily found in Europeans and are rarely found in East Asians (*106-113*). Three SNPs (rs4988235, rs182549 and rs145946881) located on the *MCM6* gene are associated with lactase persistence in adults (*114-116*) and the derived alleles are primarily found in present-day Europeans and Africans, and are rare in present-day East Asians (*114-116*). In ancient individuals from the Tibetan Plateau, the derived alleles for *HERC2*, *KITLG*, *IRF4*, *SLC24A5*, *SLC45A2*, *MC1R*, *MCM6*, and *TYR* are mostly absent (Table S23), consistent with observations from present-day Tibetans.

The SNP rs1800414 from *OCA2* contributes to skin tone difference between Africans and East Asians (*117*). The derived allele is mainly found in East Asians, with present-day Han populations possessing an allele frequency of 0.45-0.77 (*118*). Present-day Tibetans show an allele frequency of 0.09 (n = 33). The derived allele is rare in ancient individuals from the Tibetan Plateau, with only six individuals possessing more than two reads supporting the presence of the derived allele (Table S23).

ADH1B*47His and ALDH2*504Lys are derived mutations on the alcohol metabolizing pathway. Ethanol is first converted into acetaldehyde by alcohol dehydrogenase (ADH), and then acetaldehyde is metabolized into acetate by aldehyde dehydrogenase (ALDH2) (*119*). The derived allele for ALDH2 at rs671 greatly reduces activity of the ALDH2 enzyme (*120*). As a result, this allele contributes to the accumulation of acetaldehyde, which is a mutagen and animal carcinogen. This allele is associated with facial flushing after drinking alcohol, and it is also associated with disorders including alcohol liver disease and esophageal cancer. Population genetic study showed that this allele is specific to East Asians, with the highest allele frequency found in southeastern China (*121*). The derived allele for *ALDH2* is not found in ancient individuals of the Tibetan Plateau except for one read supporting the derived allele in Chokhopani and Mebrak, respectively (Table S23). Present-day Tibetans show an allele frequency of 0.015 (n = 33), which is similarly low. The derived allele at rs1229984 in *ADH1B*, the gene that is coding for alcohol dehydrogenase, speeds up the breakdown of ethanol into acetaldehyde and its frequency is high in East Asians, but rare in Africans, Europeans

and Americans (*122*). In China, the derived allele has an east to west cline, with the highest frequency of 98.5% in the southeast Chinese province of Zhejiang (*123*). The derived allele is only found in four individuals with more than two reads supporting the presence of the derived allele (Table S23). Present-day Tibetans show a higher frequency of the derived allele, of 16.7%, suggesting that the derived allele has increased over the past 3,000 years.

<u>qpGraph analysis</u>

To model ancestry among the ancient plateau individuals, we constructed admixture graphs using qpGraph, with CpG sites excluded. To capture deeply diverged ancestries sampled to date from Asia, we included Tianyuan, La368 (from Southeast Asia (*98*)), and the present-day Onge to construct the base graph. To the base graph, we added Bianbian, an early Neolithic individual from Shandong, which was modeled as a mixture of the lineages related to Tianyuan and the Onge (Fig. S33A). To model the ancient plateau populations, we added Shannan3k. The important observation for this model shows that the major source of Tibetan ancestry is related to Bianbian, and the minor source is from the trifurcation point where the three deep lineages diverged (Fig. S33B). This is in agreement with the *qpAdm* models which show that the deep lineage could be related to Onge or Tianyuan (Table S14-S15). We subsequently added Zongri5.1k and Chamdo2.8k_1 to capture the three plateau ancestries characterized in this study (Fig. 2E).

Supplementary Figures

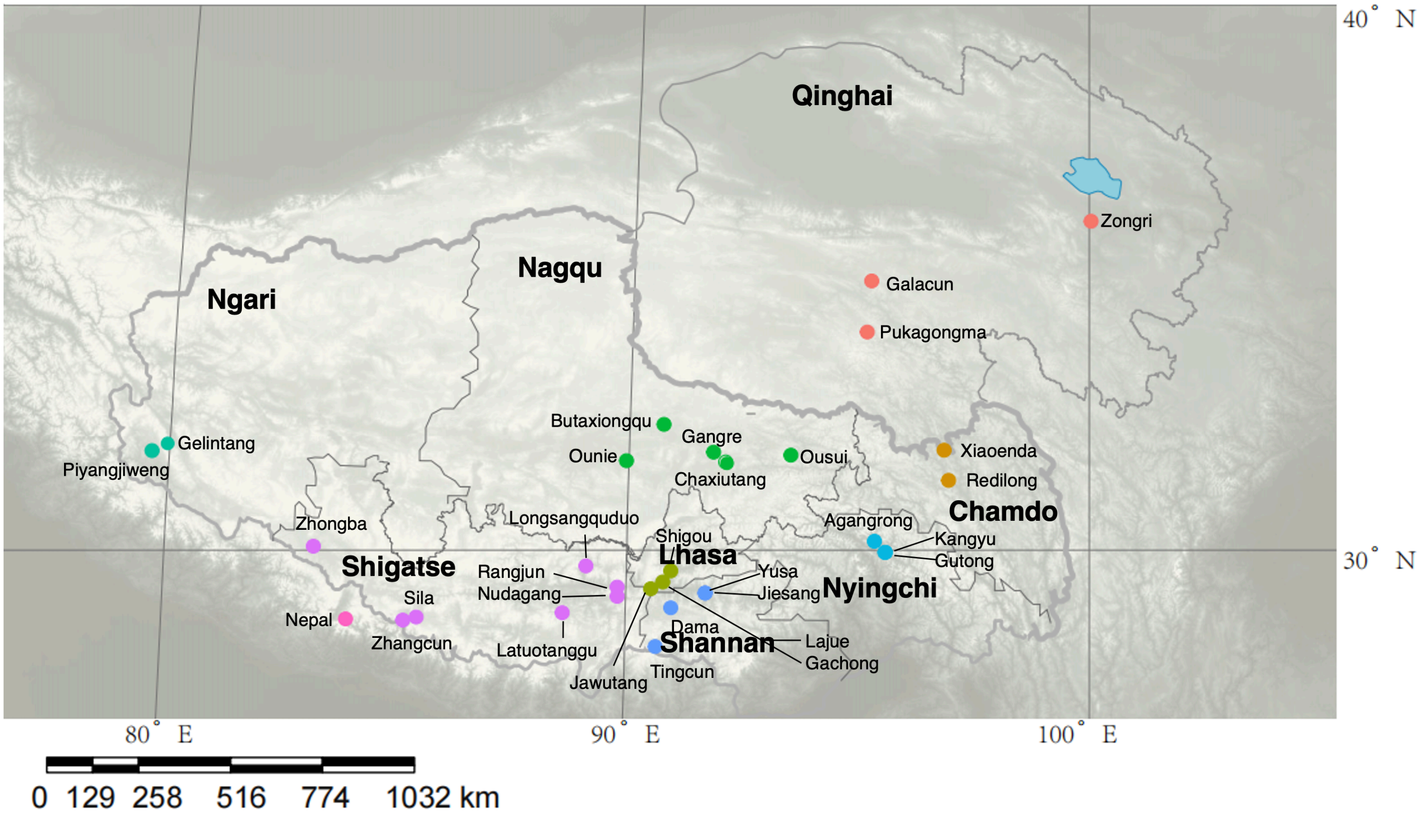

Fig. S1. Map of the archaeological sites in this study. The seven prefectures of Tibet Autonomous Region are shown.

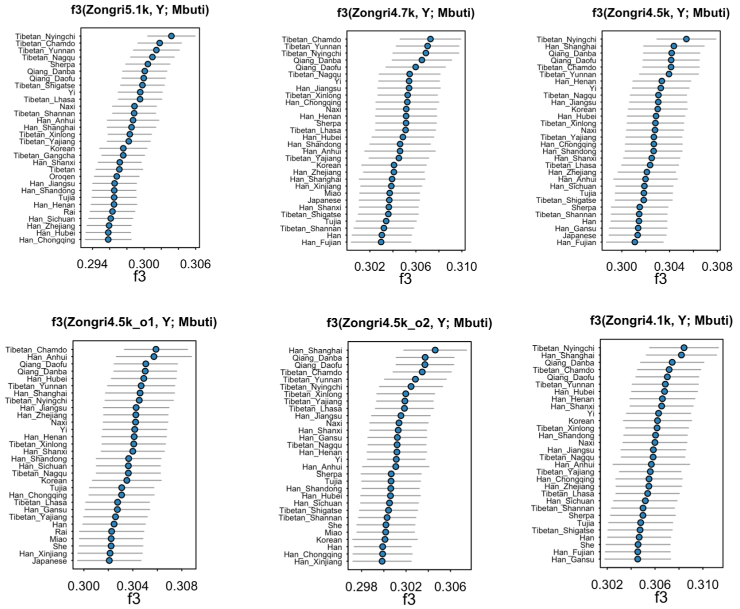

Fig. S2. Outgroup *f₃*-statistics for the Zongri groups. For each comparison of six different genetic groups from the Zongri site, we show the top 30 present-day populations that share the highest genetic drift with the corresponding Zongri group. The bar represents ±1 standard error.

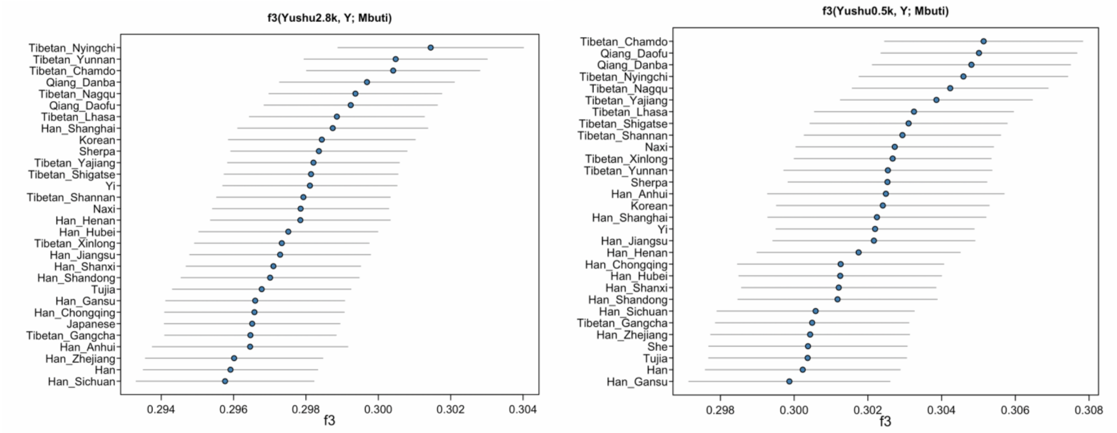

Fig. S3. Outgroup *f₃*-statistics for groups from the Yushu prefecture, dating to 2,800 BP and 500 BP. We show the top 30 present-day populations that show the highest genetic similarity to the (A) Yushu2.8k group and (B) Yushu0.5k group. The bar represents ±1 standard error.

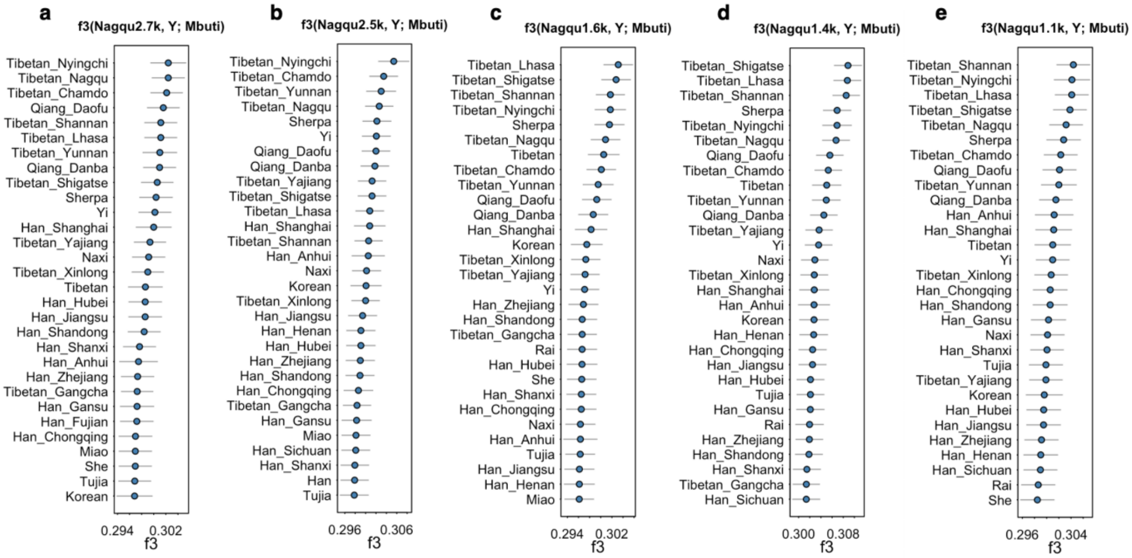

Fig. S4. Outgroup f_3-statistics for groups from the Nagqu prefecture. We show the top 30 present-day populations that show the highest genetic similiarity to (A) Nagqu2.7k (Ousui), (B) Nagqu2.5k (Butaxiongqu), (C) Nagqu1.6k (Gangre), (D) Nagqu1.4k (Ounie), and (E) Nagqu1.1k (Chaxiutang) groups. The bar represents ±1 standard error.

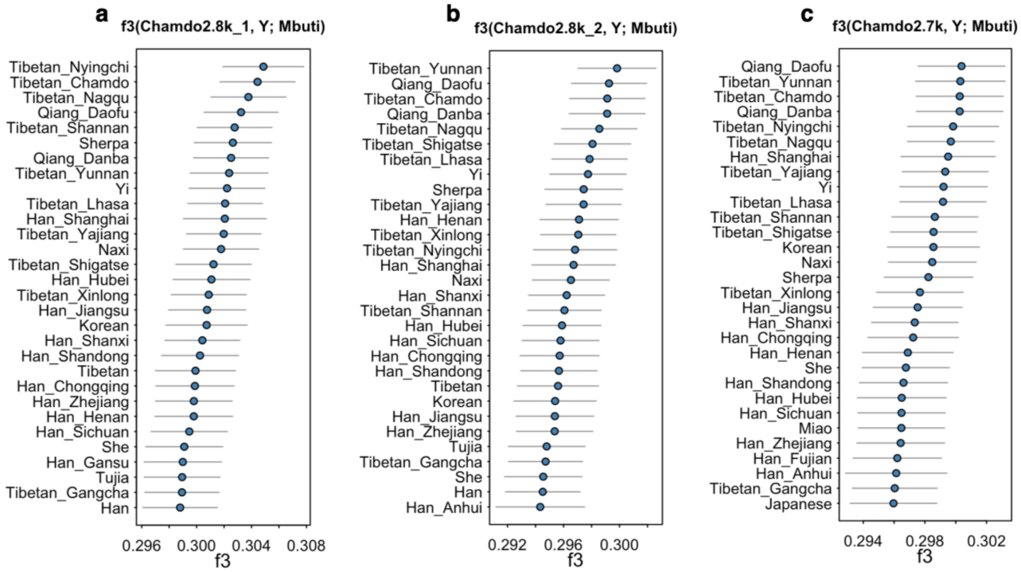

Fig. S5. Outgroup f_3-statistics for ancient individuals from the Chamdo prefecture. We show the top 30 present-day populations that show the highest genetic similarity to (A) Chamdo2.8k_1, (B) Chamdo2.8k_2, and (C) Chamdo2.7k groups. The bar represents ±1 standard error.

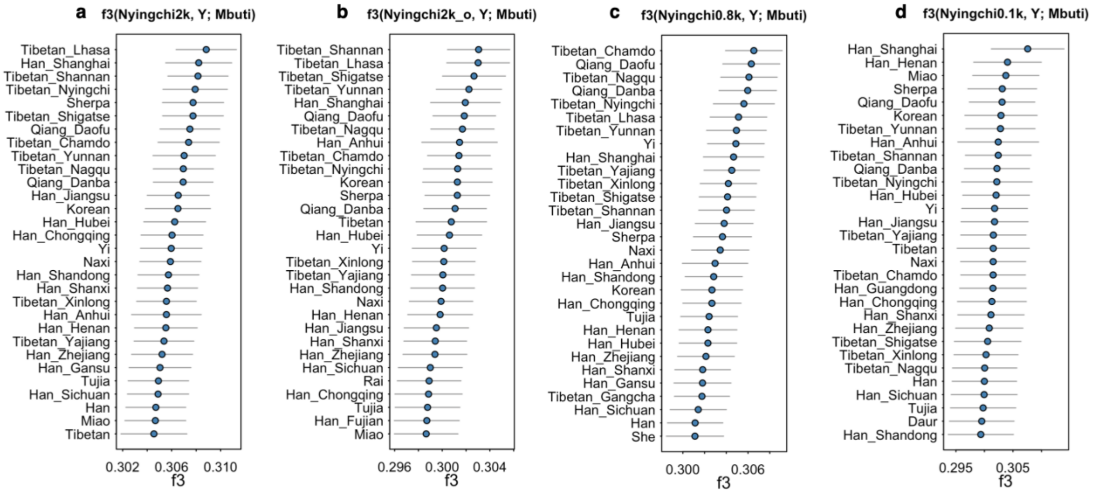

Fig. S6. Outgroup f_3-statistics for ancient individuals from the Nyingchi prefecture. We show the top 30 present-day populations that show the highest genetic similarity to the (A) Nyingchi_2k, (B) Nyingchi_2k_o, (C) Nyingchi_0.8k, and (D) Nyingchi_0.1k groups. The bar represents ±1 standard error.

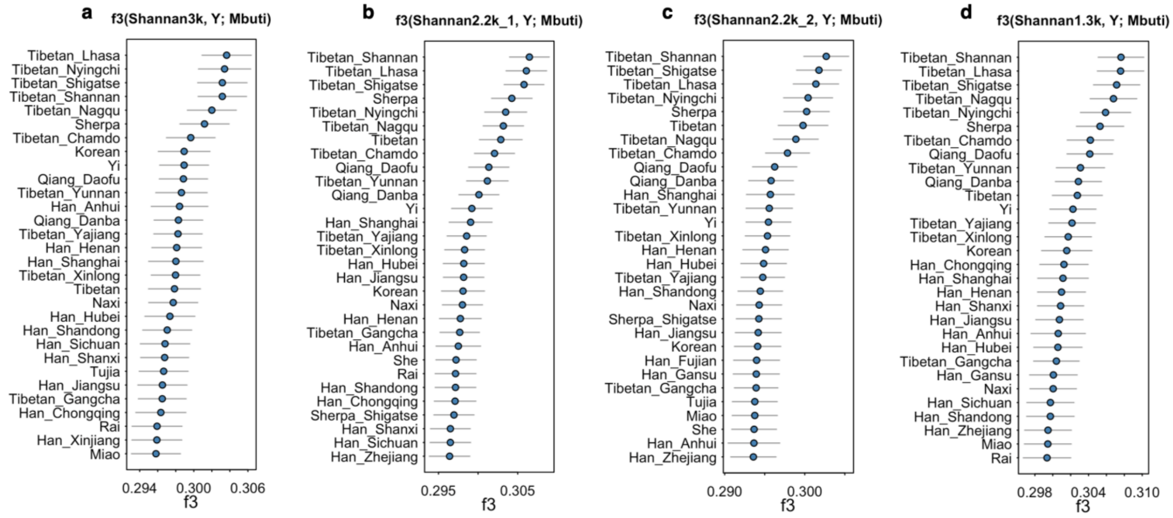

Fig. S7. Outgroup f_3-statistics for ancient individuals from the Shannan prefecture. We show the top 30 present-day populations that show the highest genetic similiarity to (A) Shannan_3k, (B) Shannan_2.2k_1, and (C) Shannan_2.2k_2 groups. The bar represents ±1 standard error.

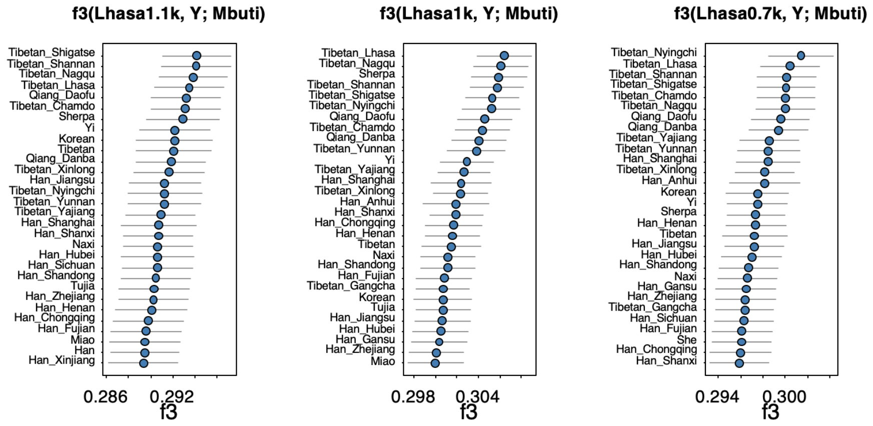

Fig. S8. Outgroup f_3-statistics for ancient individuals from Lhasa. We show the top 30 present-day populations that show the highest genetic similarity to the (A) Lhasa1.1k (Rangjun), (B) Lhasa1k (Gachong), and (C) Lhasa0.7k (Shigou) groups. The bar represents ±1 standard error.

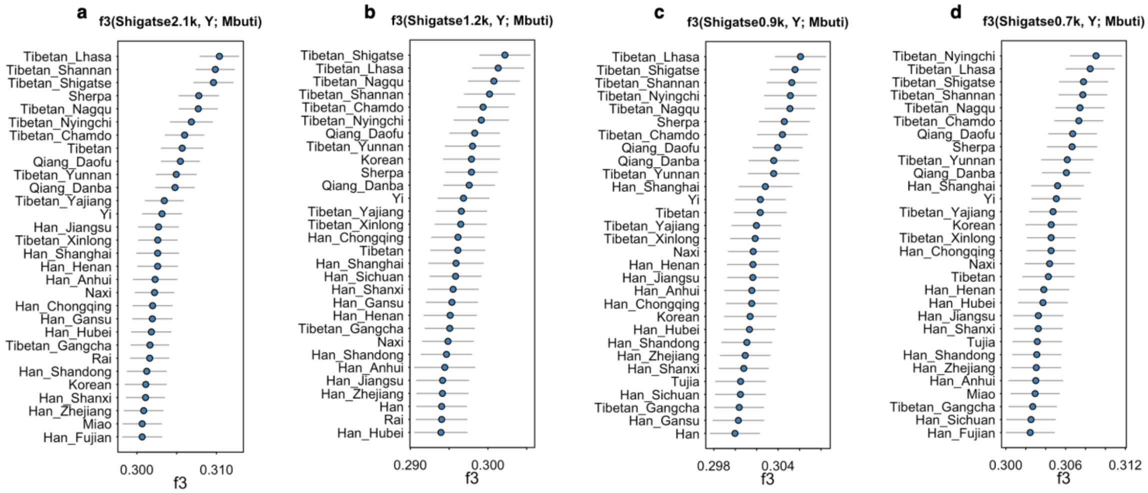

Fig. S9. Outgroup f_3-statistics for ancient individuals from the eastern part of the Shigatse prefecture. We show the top 30 present-day populations that show the highest genetic similiarity to (A) Shigatse_2.1k (Nudagang), (B) Shigatse_1.2k (Rangjun), (C) Shigatse_0.9k (Longsangquduo), and (D) Shigatse_0.7k (Latuotanggu) groups. The bar represents ±1 standard error.

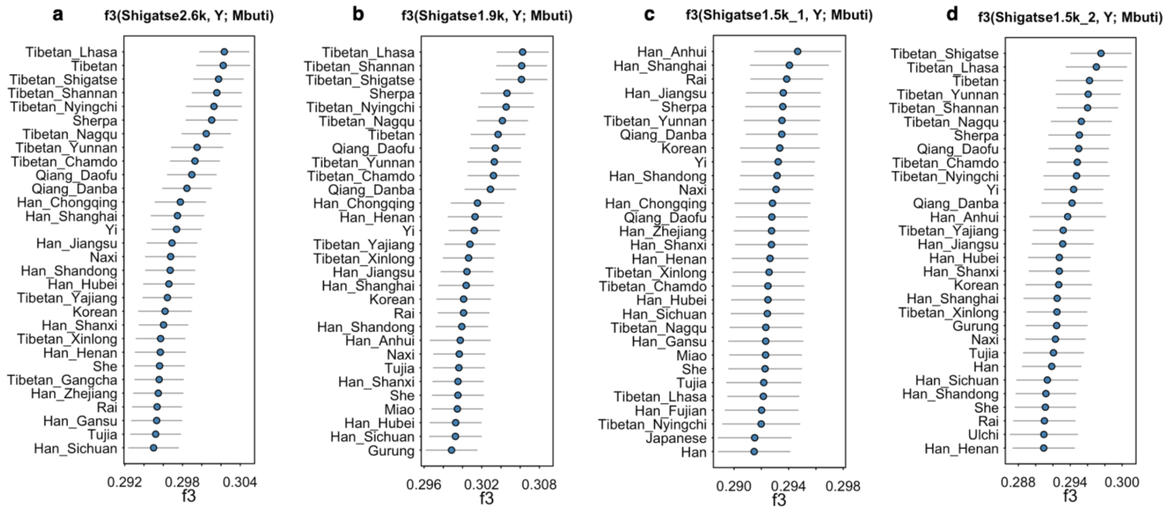

Fig. S10. Outgroup *f*$_3$-statistics for ancient individuals from the western part of the Shigatse prefecture. We show the top 30 present-day populations that show the highest genetic similarity to (A) Shigatse_2.6k (Sila), (B) Shigatse_1.9k (Sding Chung), (C) Shigatse_1.5k_1 (Zhangcun), (D) Shigatse_1.5k_2 (Zhangcun). The bar represents ±1 standard error.

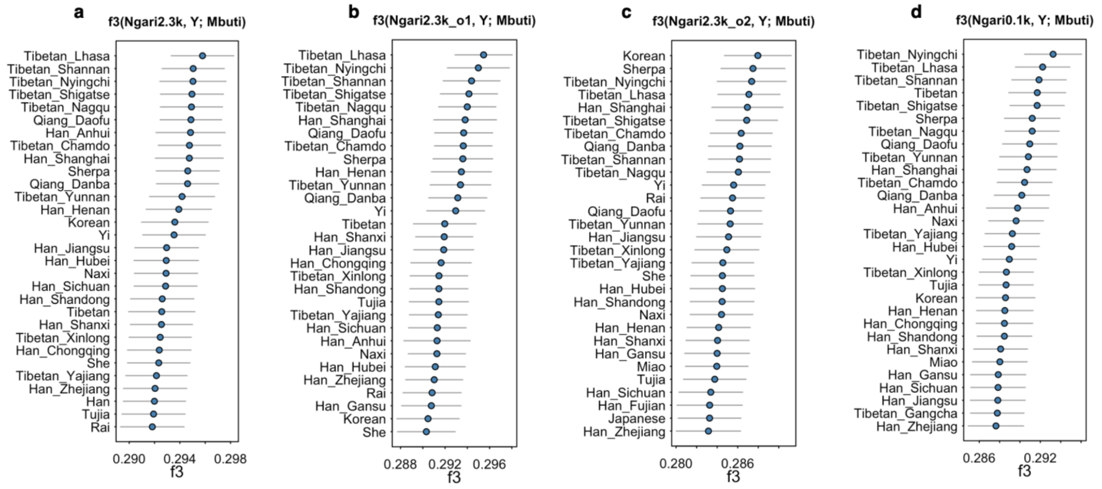

Fig. S11. Outgroup *f*$_3$-statistics for ancient individuals from the Ngari prefecture. We show the top 30 present-day populations that show the highest genetic similiarity to (A) Ngari2.3k, (B) Ngari2.3k_o1, and (C) Ngari2.3k_o2 groups from Piyangjiweng; and the (D) Ngari0.1k group from Gelintang. The bar represents ±1 standard error.

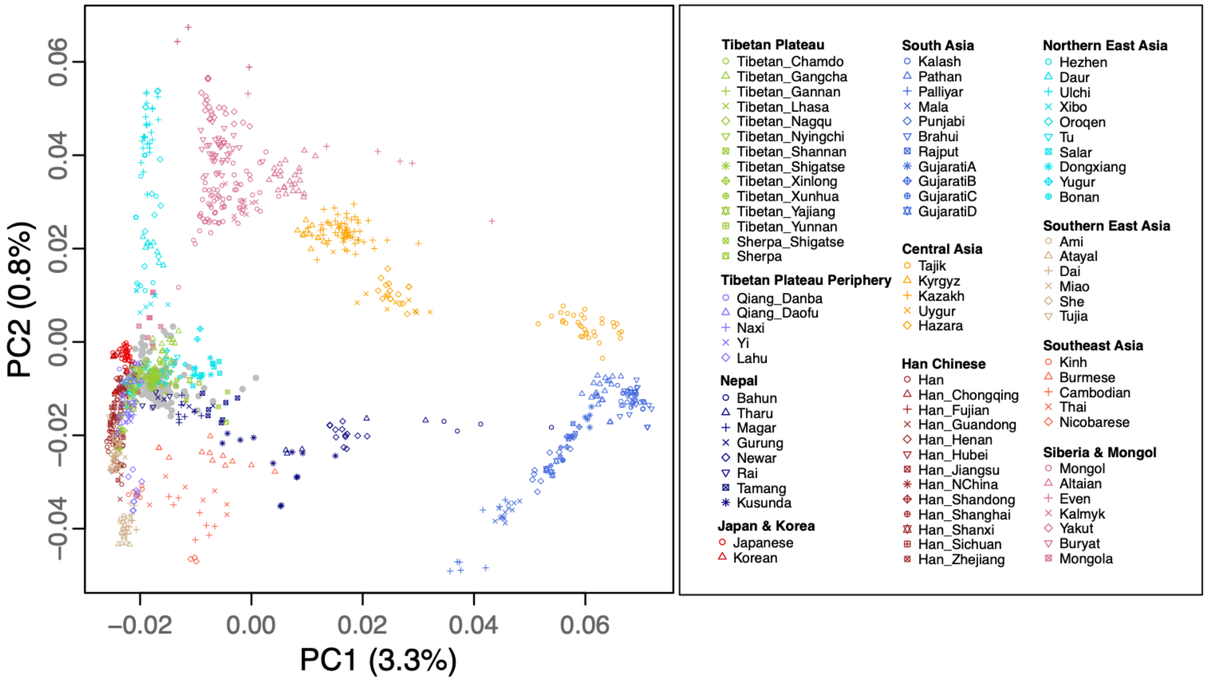

Fig. S12. Principle component analysis present-day populations on or near the Tibetan Plateau onto which ancient individuals were projected. The PCA is the same as that reported in Fig. 2B-2D, but with ancient individuals shown in gray and present-day populations colored according to their geography, as indicated in the legend. The gray dots denote ancient samples.

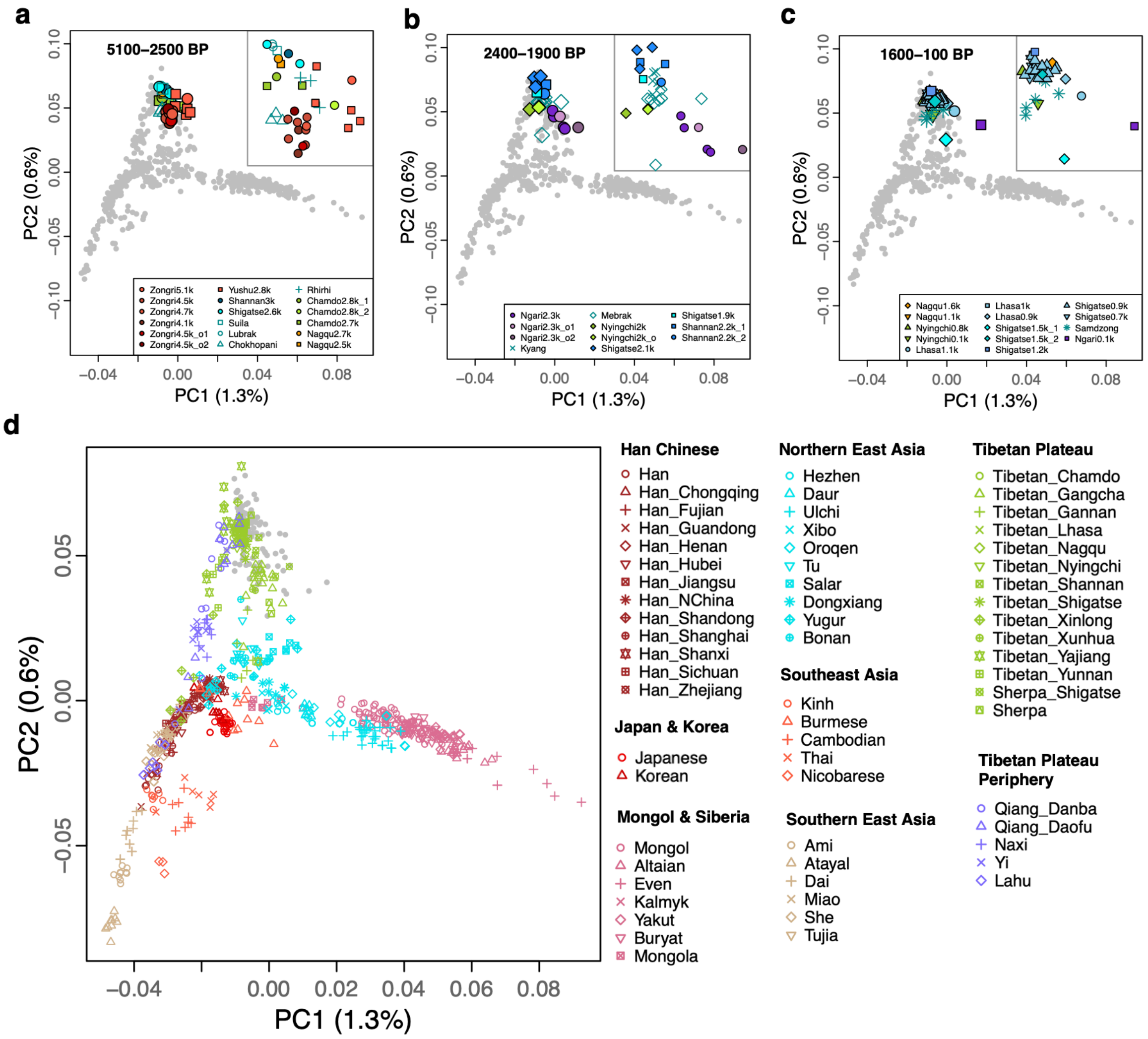

Fig. S13. PCA of present-day Southeast and East Asian populations with ancient individuals projected in (A-C). Present-day populations are denoted in gray in (A-C) and denoted by a color corresponding to their region (D).

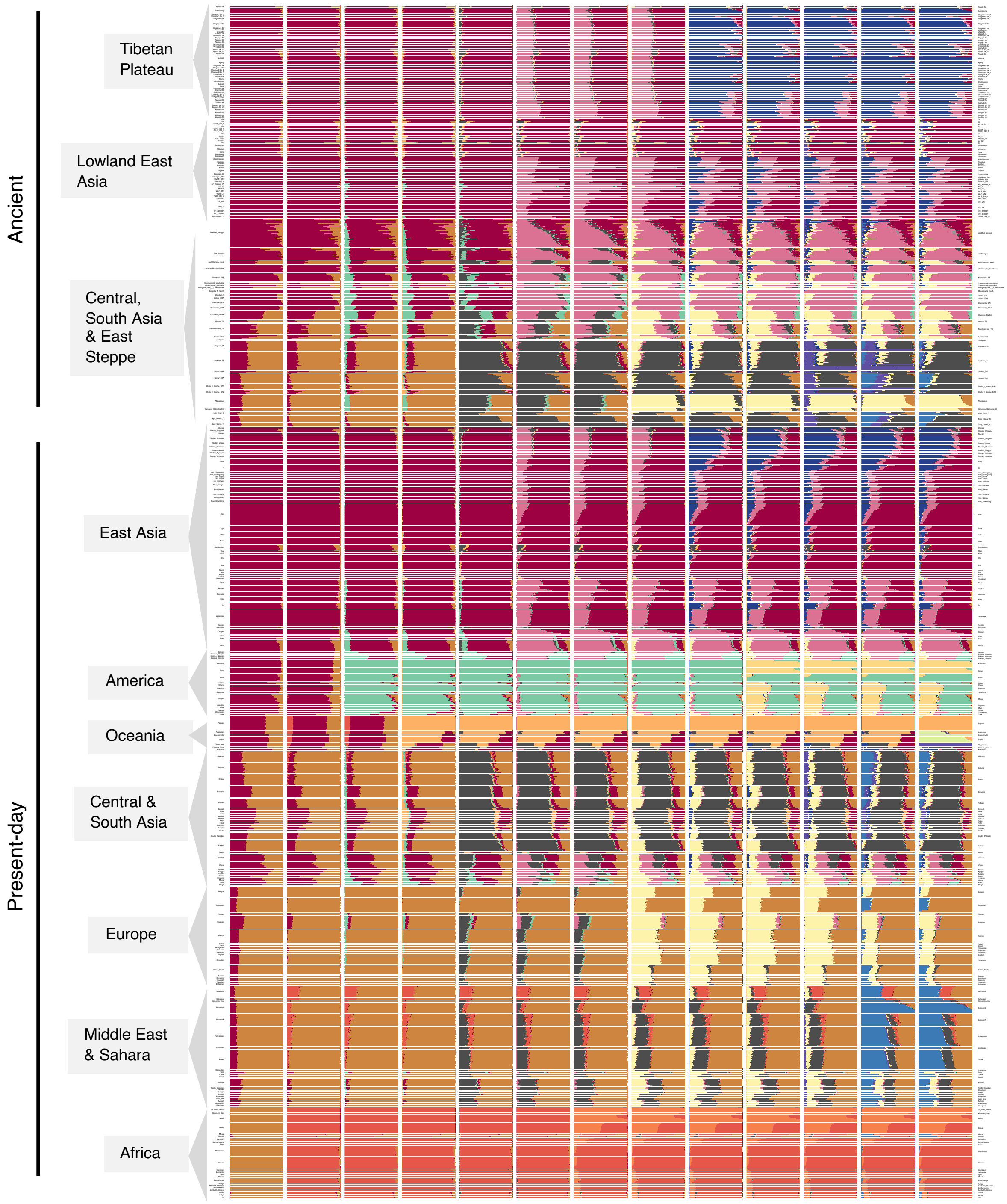

Fig. S14. ADMIXTURE analysis including diverse ancient and present-day humans ranging from K=2 to K=14.

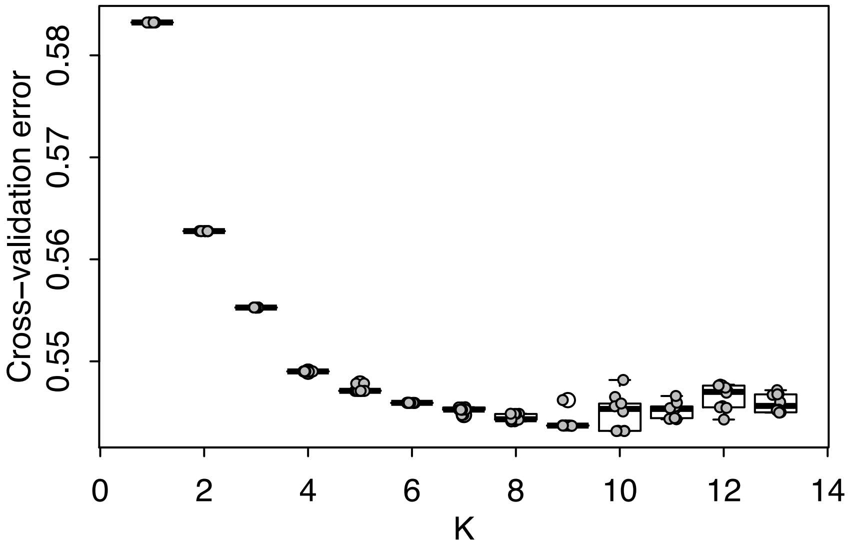

Fig. S15. Cross-validation errors of admixture analysis varying K from 2 to 14.

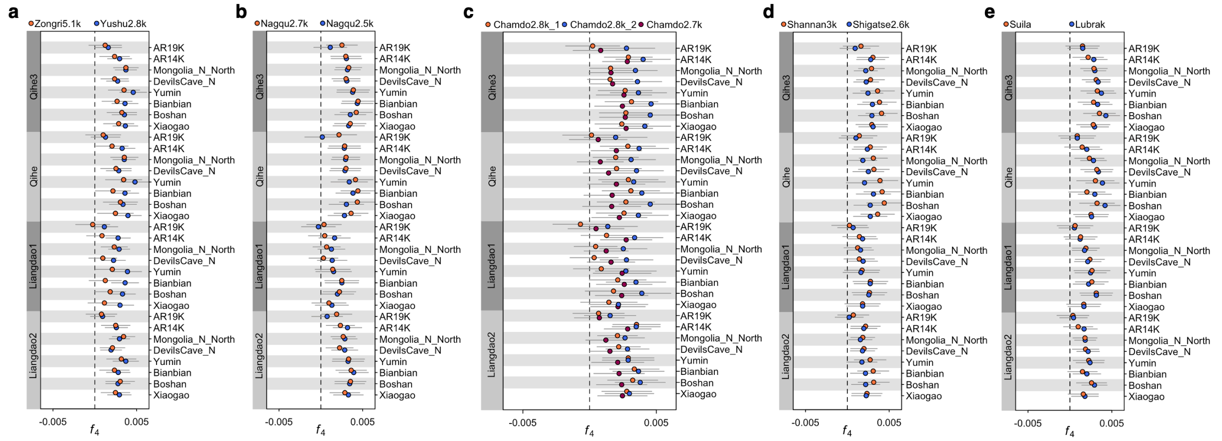

Fig. S16. F_4-statistics showing that Early Ancient Tibetans (5,100-2,500 BP) share more alleles with lowland Neolithic northern East Asians (NEA) than lowland Neolithic southern East Asians (SEA). The statistics f_4 (NEA, SEA; Early Ancient Tibetans, Mbuti) are shown with ±3 standard errors, with the Early Ancient Tibetans shown at the top, the NEA shown on the right, and the SEA shown on the left. A more positive f_4 value indicates that the Early Ancient Tibetan group shares more alleles with the SEA than the NEA.

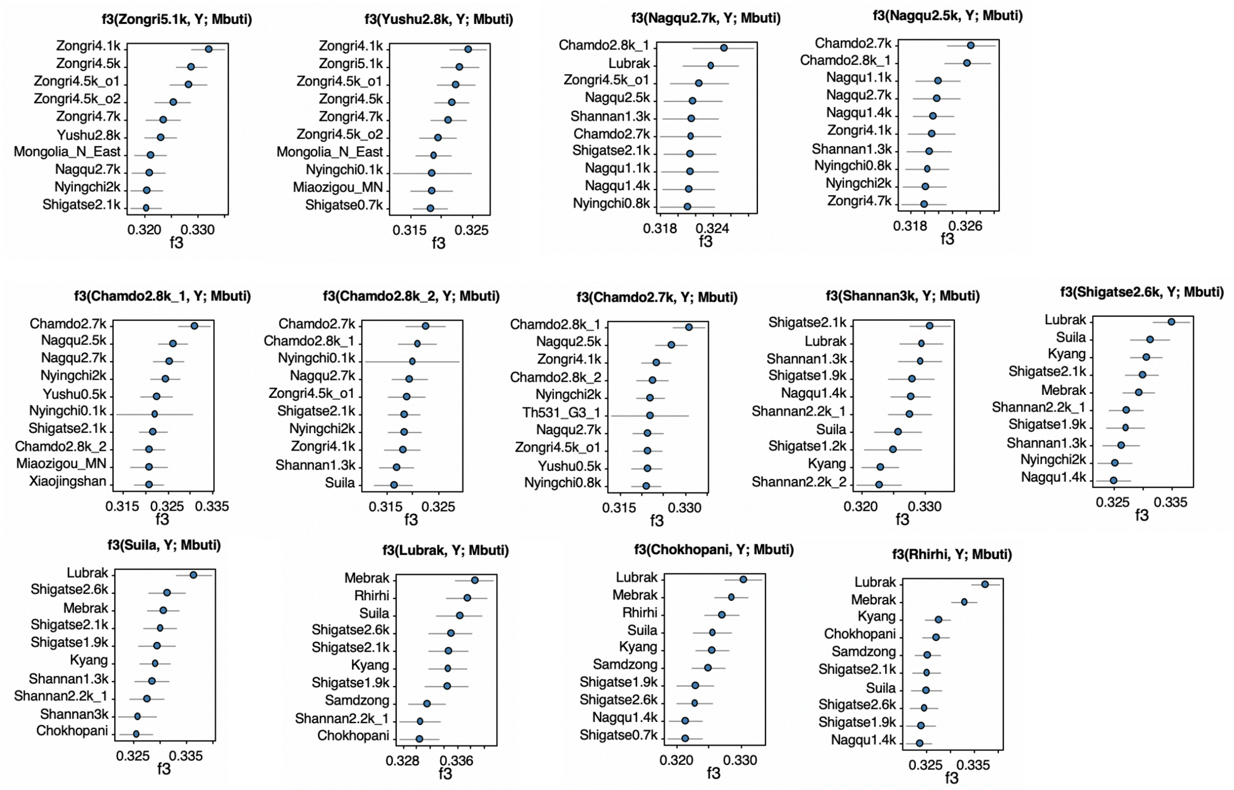

Fig. S17. The top 10 populations among all present-day and ancient populations showing the highest outgroup-f_3 statistics with Early Ancient Tibetans.

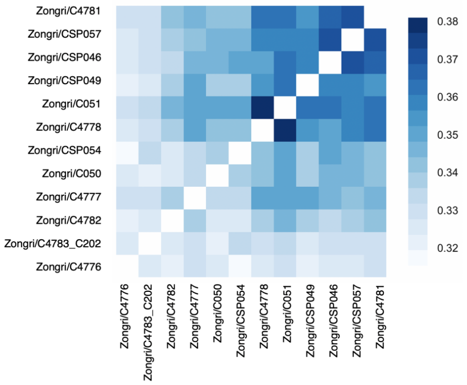

Fig. S18. The clustering of Zongri individuals using outgroup-f_3 statistics. Outgroup-f_3 analysis for f_3 (X, Y; Mbuti) where Mbuti is a central African present-day population and X and Y are individuals from the Zongri site. Darker blue indicates higher genetic similarity.

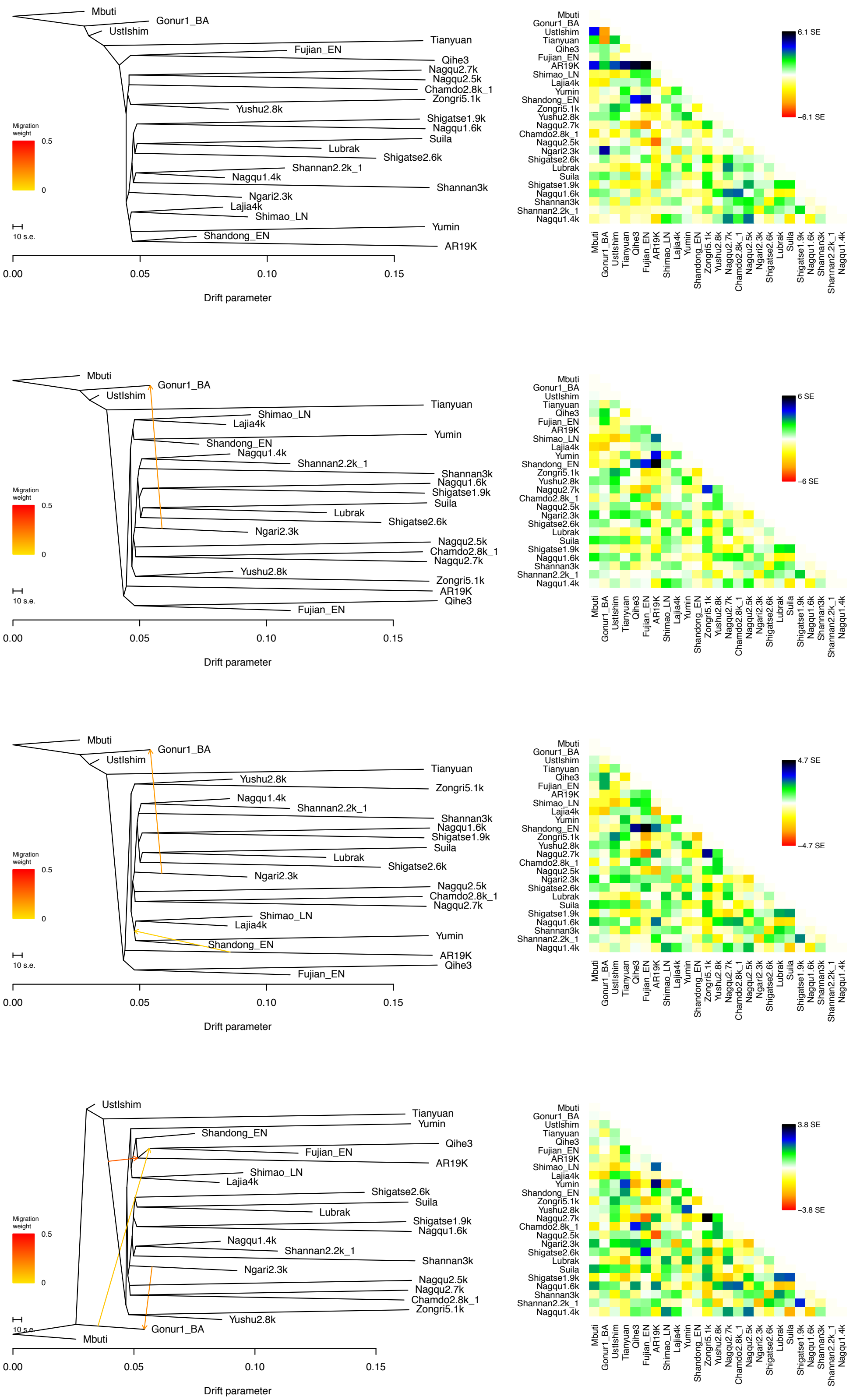

Fig. S19. TreeMix analysis with migration edges ranging from 0 to 3.

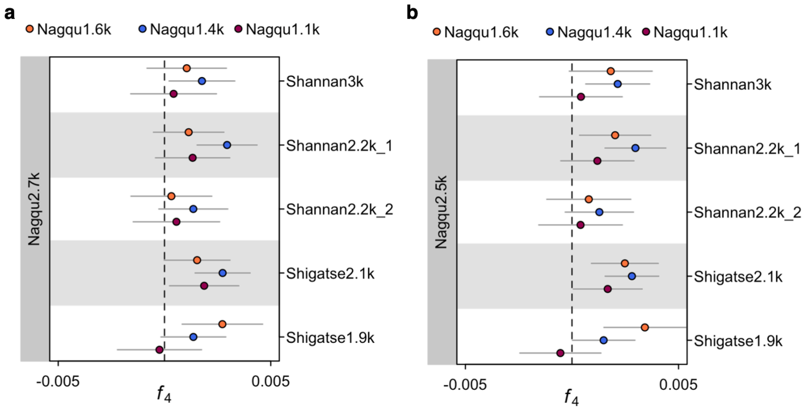

Fig. S20. F_4-statistics showing a genetic shift in the Nagqu prefecture of increased southern plateau ancestry. The statistics f_4(Shannan and Shigatse groups, older Nagqu groups; younger Nagqu groups, Mbuti) are shown with ±3 standard errors. In (A), the Nagqu2.7k group was used, while in (B) the Nagqu2.5k group was used to represent southeastern plateau ancestry, while the 3,000-1,900-year-old individuals from the Shannan and Shigatse prefectures were used to represent the southwestern and southern plateau ancestry. A more positive f_4 value indicates that the 1,600-1,100-year-old individuals from the Nagqu prefecture shares more alleles with the Shannan/Shigatse groups than the older Nagqu group.

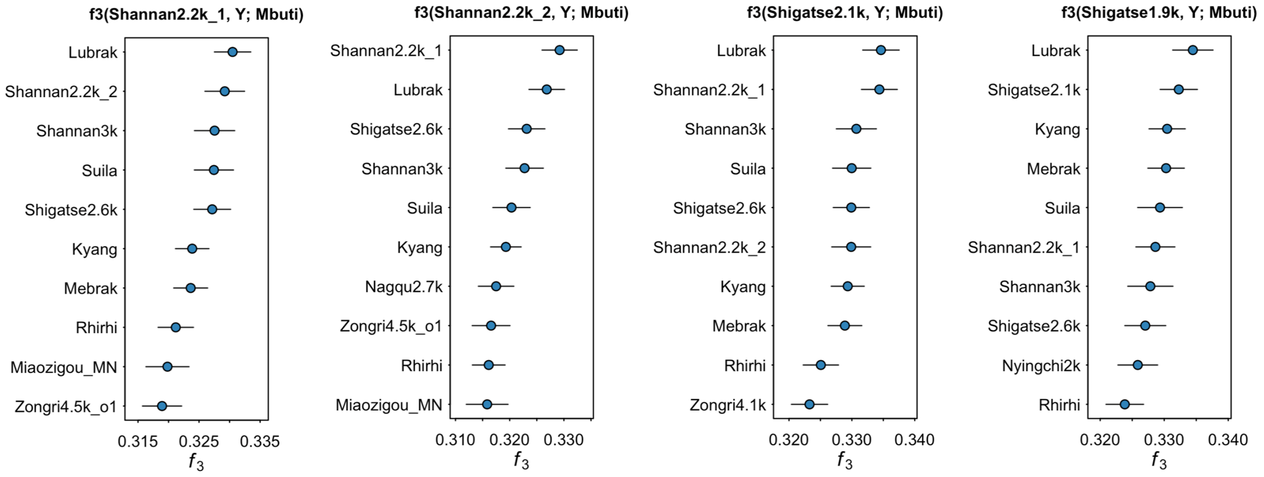

Fig. S21. The top 10 populations showing the highest outgroup-f_3 statistics for 2,200-1,900-year-old groups from the Shannan and Shigatse prefectures. Ancient individuals younger than the test population were excluded.

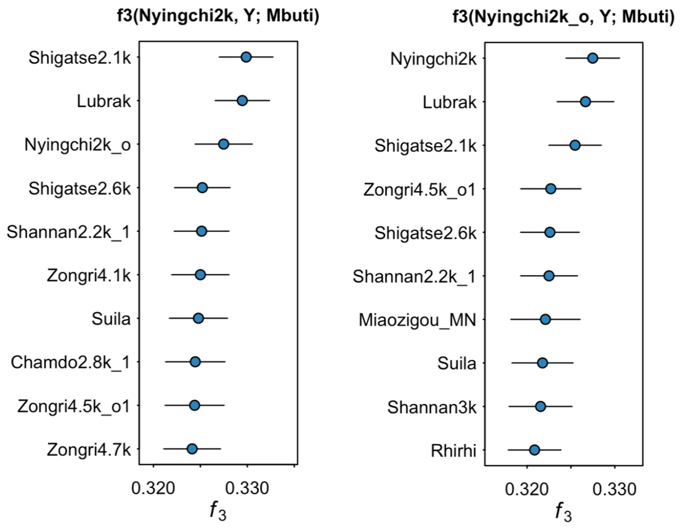

Fig. S22. The top 10 populations showing the highest outgroup-*f₃* statistics for the 2,000-year-old group from the Nyingchi prefecture. Ancient individuals younger than the test population were excluded.

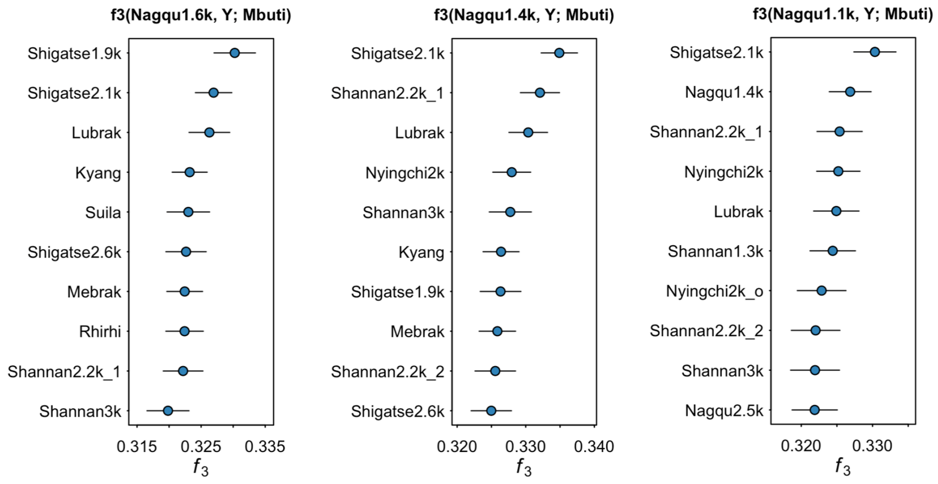

Fig. S23. The top 10 populations showing the highest outgroup-*f₃* statistics for 1,600-1,100-year-old groups from the Nagqu prefectures. Ancient individuals younger than the test population were excluded.

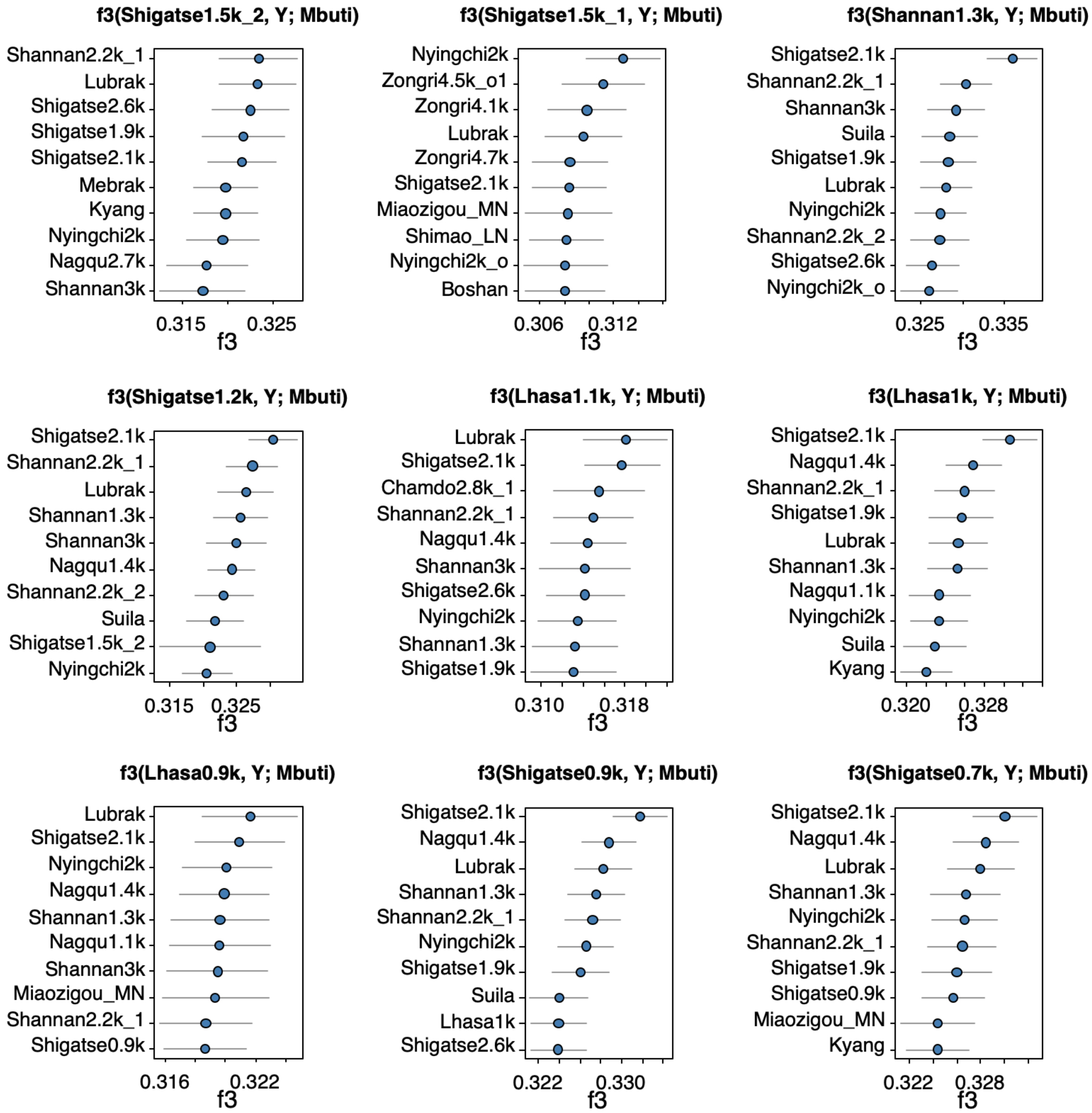

Fig. S24. The top 10 populations showing the highest outgroup-f3 statistics for 1,500-700-year-old groups from Shigatse and Shannan prefectures. Ancient individuals younger than the test population were excluded.

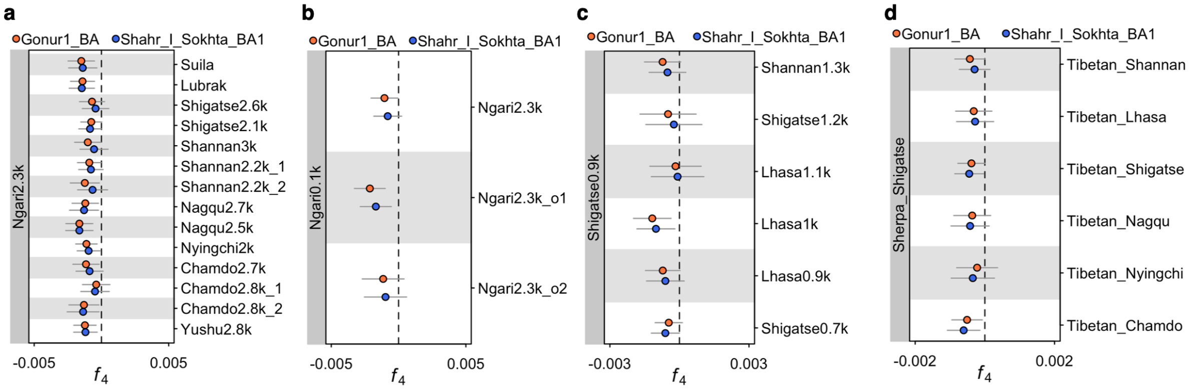

Fig. S25. F_4-statistics showing the influence of Central Asian ancestry on the Tibetan Plateau. F_4-statistics are shown with ±3 standard errors. (A) shows f_4 (Ngari2.3k, diverse ancient plateau individuals; Bronze Age Central Asians, Mbuti); (B) shows f_4 (Ngari0.1k, Ngari2.3k and outliers; Bronze Age Central Asians, Mbuti); (C) shows f_4 (1,300-700-year-old individuals from the southern and southwestern plateau, Shigatse0.9k; Bronze Age Central Asians, Mbuti); and (D) shows f_4 (present-day Tibetans, present-day Sherpa; Bronze Age Central Asians, Mbuti). A more negative f_4 value indicates the plateau individuals listed on the left shares more alleles with Bronze Age Central Asians than the plateau individuals listed on the right. Bronze-age Central Asian populations are from Turkmenistan (Gonur1_BA) and Iran (Shahr_I_Sokhta_BA1).

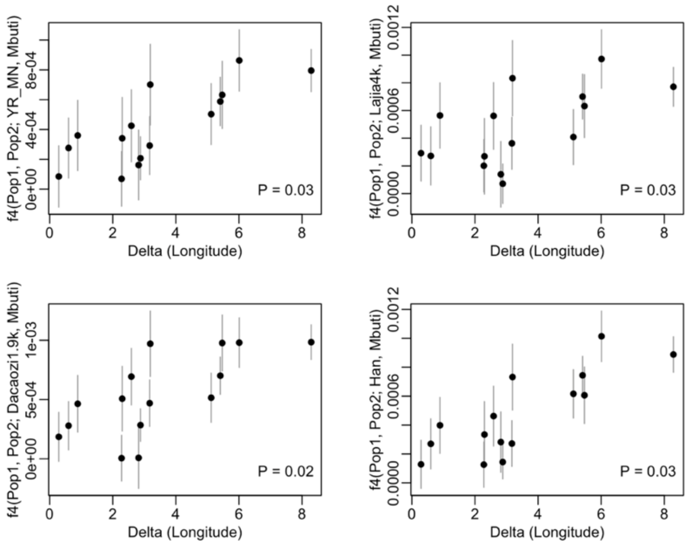

Fig. S26. Testing the correlation between longitudinal distance and differences in allele sharing with lowland East Asians for present-day Tibetan population pairs. Each dot represents data from a pair of Tibetan populations. The vertical bar represents ± 1SE. The P values were calculated using the Mantel test which accounted for the non-independence of pairwise data. The plots are based on data from 6 populations: Tibetan_Chamdo, Tibetan_Lhasa, Tibetan_Nyingchi, Tibetan_Shannan, Tibetan_Shigatse and Tibetan_Nagqu.

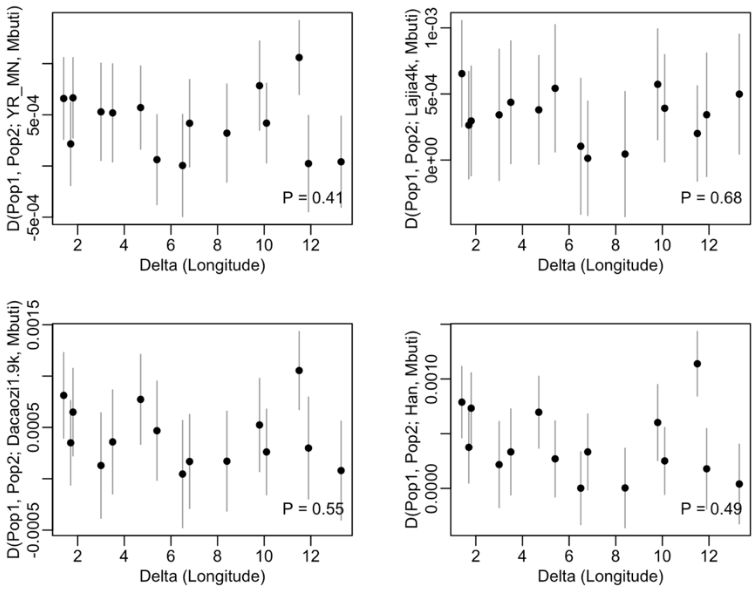

Fig. S27. Testing the correlation between longitudinal distance and differences in allele sharing with lowland East Asians for pairs of ancient Tibetans spanning 3,000-2,600 BP. Each dot represents data from a pair of Tibetan populations. The vertical bar represents ± 1SE. The P values were calculated using the Mantel test which accounted for the non-independence of pairwise data. The plots are based on data from 6 populations: Yushu2.8k, Nagqu2.7k, Chamdo2.8k_1, Shigatse2.6k, Lubrak and Shannan3k.

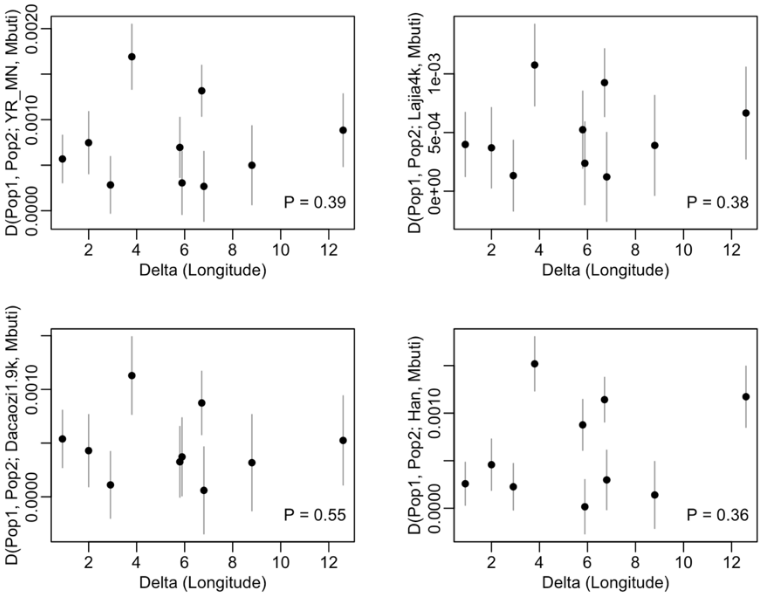

Fig. S28. Testing the correlation between longitudinal distance and differences in allele sharing with lowland East Asians for pairs of ancient Tibetans spanning 2,200-1,700 BP. Each dot represents data from a pair of Tibetan populations. The vertical bar represents ± 1SE. The P values were calculated using the Mantel test which accounted for the non-independence of pairwise data. The plots are based on data from 5 populations: Shannan2.2k_1, Shigatse2.1k, Shigatse1.9k, Nyingchi2k and Mebrak.

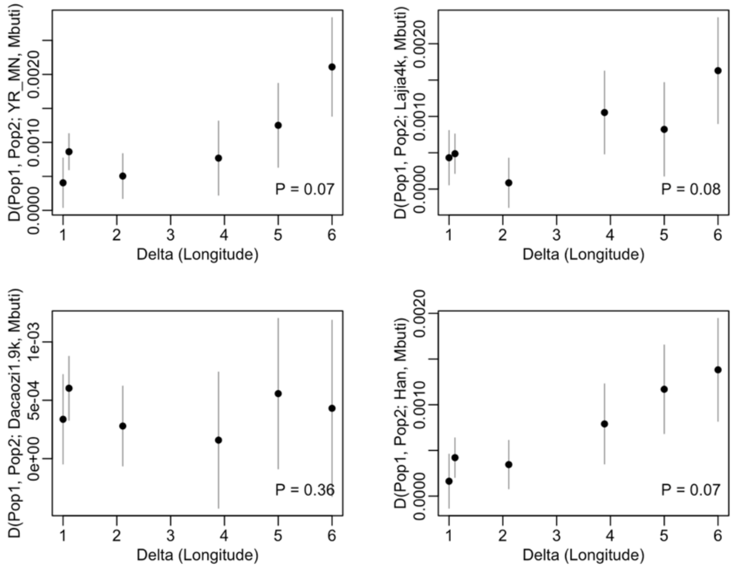

Fig. S29. Testing the correlation between longitudinal distance and differences in allele sharing with lowland East Asians for pairs of ancient Tibetans spanning 2,500-1,200 BP. Each dot represents data from a pair of Tibetan populations. The vertical bar represents ± 1SE. The P values were calculated using the Mantel test which accounted for the non-independence of pairwise data. The plots are based on data from 4 populations: Samdzong, Shigatse1.5k_2, Nagqu1.4k, and Shannan1.3k.

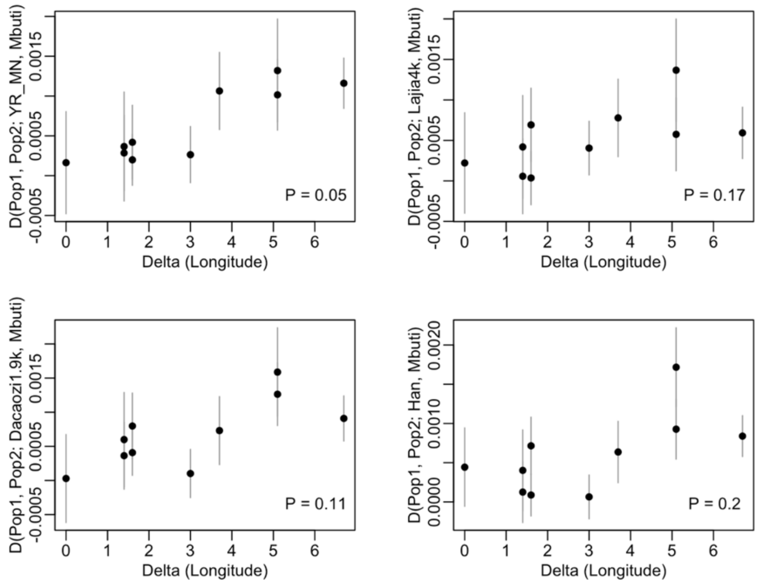

Fig. S30. Testing the correlation between longitudinal distance and differences in allele sharing with lowland East Asians for pairs of ancient Tibetans spanning 1,200-800 BP. Each dot represents data from a pair of Tibetan populations. The vertical bar represents ± 1SE. The P values were calculated using the Mantel test which accounted for the non-independence of pairwise data. The plots are based on data from 5 populations: Nagqu1.1k, Lhasa1.1k, Lhasa1k, Shigatse0.9k and Nyingchi0.8k.

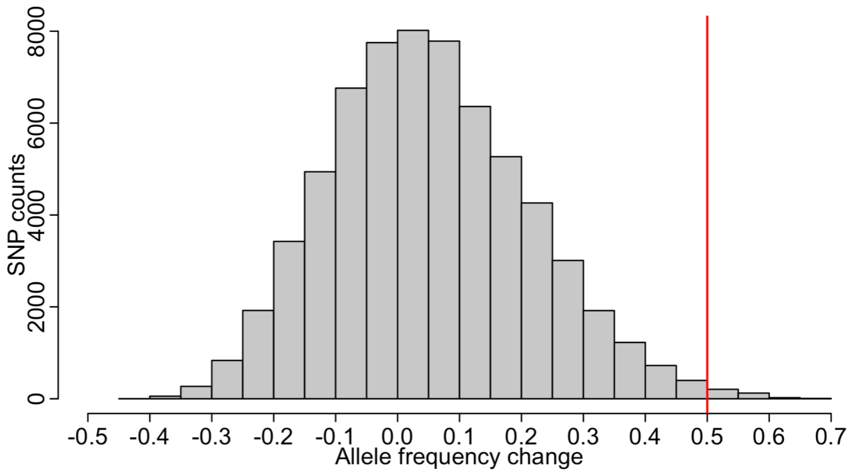

Fig. S31. The null distribution of frequency changes for an allele with initial frequency at 0.35-0.55 in populations over the past 3,400 years on the Tibetan Plateau. The null distribution was constructed by taking SNPs with allele frequency similar to that of the *EPAS1* allele which is 0.36. In total, 65,296 SNPs were obtained and the allele frequency change at these SNPs between ancient and present-day populations were calculated.

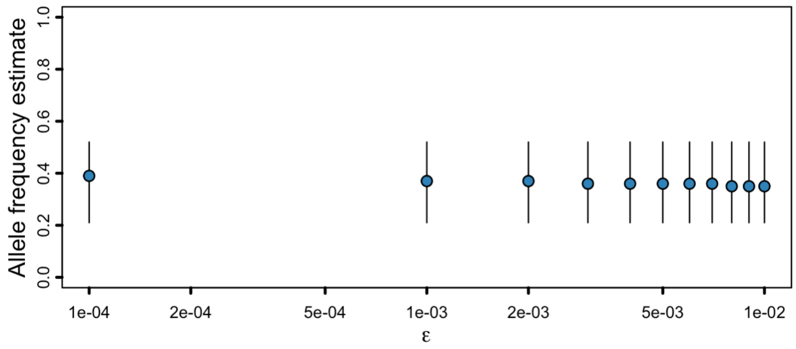

Fig. S32. *EPAS1* allele frequency estimate in the Early Ancient Tibetans using different ancient DNA error rate parameters (epsilon). Varying the error rate, the allele frequency estimate remains highly consistent, showing that the method is robust to the ancient DNA error rate. The bars represent the 95% confidence intervals.

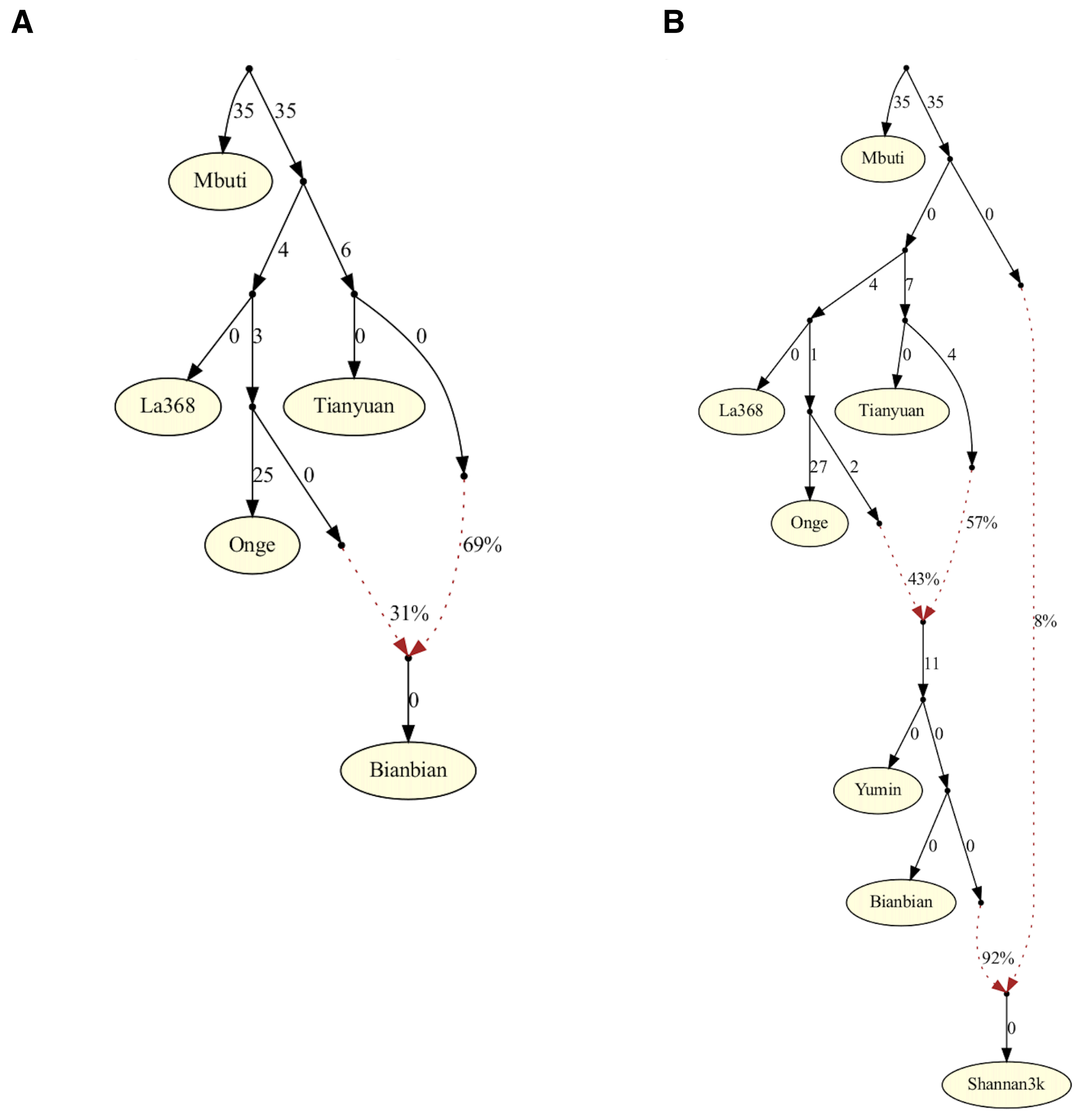

Fig. S33. Intermediate admixture graphs. In (A), an Early Neolithic northern East Asian individual (Bianbian) was added. The worst |Z|-score is 0.341, observed for f4 (Mbuti, Tianyuan; La368, Onge). In (B), Yumin, and Shannan3k were added, to model ancestry on the plateau. The worst |Z|-score is 2.349, observed for f4 (Tianyuan, La368; Bianbian, Shannan3k). The final admixture graph is shown in Fig. 2E.

Supplementary Tables

Table S1. The sample information for all the ancient individuals sequenced in this study.
See Excel.

Table S2. The site, carbon dating (C14), cemetery and skeletal description information of the specimens.
See Excel.

Table S3. The library preparation, sequencing and quality control information for each ancient individual.
See Excel.

Table S4. The kinship estimates for pairs of ancient Tibetan Plateau specimens using lcMLkin. When two samples show familial relationships, the one with higher data coverage was retained. When two samples were identified as from the same individual, they are merged when they are from the same burial, otherwise, only data from the sample with higher data coverage was retained. Details can also be found in the "Sample description" section in Supplementary text.

Site	Specimen1	Specimen2	lcMLkin					Familial	Data processing
			k0_hat	k1_hat	k2_hat	pi_hat	nSNP	Relationship*	
Agangrong	C3447	C5186	0.016	0.085	0.899	0.942	4090	Self	merge data as C3447_C5186
Latuotanggu	C5171	C3426	0.372	0.431	0.197	0.412	35637	1°	keep C5171 data
Latuotanggu	C5171	C3428	0.602	0.389	0.009	0.203	34100	2°	keep C5171 data
Latuotanggu	C3426	C3428	0.049	0.945	0.007	0.479	30541	1°	Both removed, keep C5171 data
Longsangquduo	C5164	C5163	0.284	0.474	0.242	0.479	13298	1°	keep C5164 data
Nudagang	C5148	C5147	0.019	0.023	0.958	0.97	1443	Self	keep C5148 data
Ounie	C5172	C3992	0.003	0.054	0.943	0.97	30226	Self	merge data as C5172_C3992
Piyangjiweng	C4566	C4565	0.014	0.123	0.863	0.924	21282	Self	keep C4566 data
Piyangjiweng	C4570	C4565	0	0.03	0.97	0.985	3043	Self	Both removed, keep C4566 data
Piyangjiweng	C4570	C4566	0.005	0.112	0.883	0.939	4624	Self	keep C4566 data
Zongri	C050	C208	0.048	0.947	0.006	0.479	18286	1°	keep C050 data
Zongri	C056	C202	0.537	0.433	0.029	0.246	14832	2°	keep C202 data
Zongri	C4783	C202	0.006	0.154	0.84	0.917	27999	Self	merge data as C4783_C202
Zongri	C4783	C056	0.52	0.462	0.019	0.249	16772	2°	Both removed, keep C202 data
Zongri	C4775	C205	0.44	0.553	0.007	0.283	31508	2°	Both removed, keep C4777 data
Zongri	C4777	C205	0.013	0.168	0.819	0.903	31877	Self	keep C4777 data
Zongri	C4777	C4775	0.421	0.568	0.011	0.295	39429	2°	keep C4777 data
Zongri	C4778	C4774	0.532	0.438	0.03	0.249	42721	2°	keep C4778 data
Zongri	C4779	C4778	0.432	0.536	0.031	0.299	38431	2°	keep C4778 data
Zongri	C4780	C4779	0.338	0.616	0.046	0.354	35999	2°	Both removed, keep C4778 data

Zongri	CSP048	CSP046	0.46	0.525	0.015	0.277	23022	2°		keep CSP046 data

*determined from Blouin et al (*58*)., where k2=1 indicates Self, k1=1 indicates Parent-child, k0=k2=0.25 and k1=0.5 are Full Siblings, k0=k1=0.5 are 2° relatives (e.g. half-siblings), k0=0.75 and k1=0.25 indicates 3° relatives (e.g. full cousins) and k0=1 indicates Unrelated.

Table S5. The lowest cross-validation (CV) error values of ten random-seed replicates for K=2-14.

K	2	3	4	5	6	7	8	9	10	11	12	13	14
lowest CV error	0.5832	0.5628	0.5553	0.5490	0.5471	0.5459	0.5447	0.5441	0.5437	0.5432	0.5443	0.5443	0.5450

Table S6. F_4-statistics testing the genetic homogeneity among individuals from the same archaeological site.

Site	X	Y	Z	O	f_4	Z	SNPs
Zongri	Zongri/C050	Zongri/CSP054	Zongri/C4783_C202	Mbuti	-0.003218	-3.464	137894
	Zongri/C4776	Zongri/C4781	Afanasievo	Mbuti	-0.001157	-3.178	638021
	Zongri/C4776	Zongri/C4782	Yumin	Mbuti	-0.001781	-3.027	650203
	Zongri/C4776	Zongri/C4783_C202	YR_MN	Mbuti	0.00137	3.234	734577
	Zongri/C4777	Zongri/C4781	Afanasievo	Mbuti	-0.001115	-3.086	644127
	Zongri/C4778	Zongri/C4781	Afanasievo	Mbuti	-0.001095	-3.269	694222
	Zongri/C4781	Zongri/CSP047	Afanasievo	Mbuti	0.001521	4.132	428018
	Zongri/C4781	Zongri/CSP047	Gonur1_BA	Mbuti	0.001299	3.327	418823
	Zongri/C4781	Zongri/CSP047	Lajia4k	Mbuti	0.001723	3.678	423331
	Zongri/C4781	Zongri/CSP047	Onge	Mbuti	0.001799	3.743	428016
	Zongri/C4781	Zongri/CSP047	Shamanka_EN	Mbuti	0.001568	3.872	428027
	Zongri/C4781	Zongri/CSP047	YR_MN	Mbuti	0.001888	4.287	427017
	Zongri/C4781	Zongri/CSP047	Pukagongma/CSP136	Mbuti	0.002233	3.246	248885
	Zongri/C4781	Zongri/CSP057	Afanasievo	Mbuti	0.001289	3.048	230591
	Zongri/C4783_C202	Zongri/C050	Atayal	Mbuti	-0.001973	-3.553	745975
	Zongri/C4783_C202	Zongri/C051	YR_MN	Mbuti	-0.001486	-3.08	463902
	Zongri/C4783_C202	Zongri/C4777	Atayal	Mbuti	-0.001975	-3.496	744184
	Zongri/C4783_C202	Zongri/C4777	YR_MN	Mbuti	-0.001359	-3.048	742048
	Zongri/C4783_C202	Zongri/C4778	Atayal	Mbuti	-0.001667	-3.002	817284
	Zongri/C4783_C202	Zongri/C4778	YR_MN	Mbuti	-0.001479	-3.558	814742
	Zongri/C4783_C202	Zongri/C4781	Atayal	Mbuti	-0.002073	-3.594	663396
	Zongri/C4783_C202	Zongri/C4781	Lajia4k	Mbuti	-0.001513	-3.214	655789
	Zongri/C4783_C202	Zongri/C4781	YR_MN	Mbuti	-0.002216	-4.939	661586
	Zongri/C4783_C202	Zongri/C4782	UstIshim	Mbuti	0.001648	3.213	740233
	Zongri/C4783_C202	Zongri/C4782	YR_MN	Mbuti	-0.001496	-3.331	739754
	Zongri/C4783_C202	Zongri/CSP046	UstIshim	Mbuti	0.001638	3.046	609011
	Zongri/C4783_C202	Zongri/CSP046	YR_MN	Mbuti	-0.001682	-3.754	608743
	Zongri/C4783_C202	Zongri/CSP047	UstIshim	Mbuti	0.00186	3.28	479997
	Zongri/C4783_C202	Zongri/CSP047	Pukagongma/CSP136	Mbuti	0.002284	3.266	265972

	Zongri/C4783_C202	Zongri/CSP054	Lajia4k	Mbuti	-0.002156	-3.439	145172
	Zongri/CSP046	Zongri/CSP047	YR_MN	Mbuti	0.001665	3.468	405735
	Zongri/CSP046	Zongri/CSP057	Onge	Mbuti	0.001723	3.125	220711
	Zongri/CSP047	Zongri/CSP054	Zongri/C4783_C202	Mbuti	-0.00355	-3.481	109096
Piyangjiweng	Piyangjiweng/C4563	Piyangjiweng/C4564	Redilong/CSP142	Mbuti	-0.002907	-3.307	126134
	Piyangjiweng/C4563	Piyangjiweng/C4564	Lajia4k	Mbuti	-0.002426	-3.7	156064
	Piyangjiweng/C4563	Piyangjiweng/C4564	Qihe3	Mbuti	-0.002832	-3.327	128439
	Piyangjiweng/C4563	Piyangjiweng/C4564	YR_MN	Mbuti	-0.002478	-3.865	157278
	Piyangjiweng/C4563	Piyangjiweng/C4564	Yumin	Mbuti	-0.002825	-3.474	150364
	Piyangjiweng/C4563	Piyangjiweng/C4564	Pukagongma/CSP136	Mbuti	-0.00436	-4.422	96477
	Piyangjiweng/C4563	Piyangjiweng/C4566	Afanasievo	Mbuti	0.001553	3.373	197169
	Piyangjiweng/C4563	Piyangjiweng/C4567	Luozhating/C3430	Mbuti	-0.003577	-3.492	86740
	Piyangjiweng/C4564	Piyangjiweng/C4566	Gonur1_BA	Mbuti	0.001744	4.398	438676
	Piyangjiweng/C4564	Piyangjiweng/C4566	Tianyuan	Mbuti	0.002146	3.564	433140
	Piyangjiweng/C4564	Piyangjiweng/C4566	YR_MN	Mbuti	0.001707	3.552	452270
	Piyangjiweng/C4566	Piyangjiweng/C4567	Tianyuan	Mbuti	-0.002682	-4.046	265625
	Piyangjiweng/C4566	Piyangjiweng/C4567	YR_MN	Mbuti	-0.001619	-3.016	274149
Zhangcun	Zhangcun/C5184	Zhangcun/C5185	Chokhopani	Mbuti	-0.003153	-3.138	101088
	Zhangcun/C5184	Zhangcun/C5185	Luozhating/C3430	Mbuti	-0.003696	-3.388	81386
Longsangquduo	Longsangquduo/C5159	Longsangquduo/C5164	Pukagongma/CSP136	Mbuti	-0.001989	-3.032	335938
	Longsangquduo/C5160	Longsangquduo/C5161	Onge	Mbuti	0.001474	3.004	455573
Agangrong	Agangrong/C3447_C5186	Agangrong/C3444	Qihe3	Mbuti	0.001993	3.191	354209
	Agangrong/C3445	Agangrong/C3444	AR19K	Mbuti	0.002295	3.637	353413
	Agangrong/C3445	Agangrong/C3444	Qihe3	Mbuti	0.002775	4.134	300516
Redilong	Redilong/CSP141	Redilong/CSP142	Qihe3	Mbuti	-0.002262	-3.285	229322
	Redilong/CSP141	Redilong/CSP142	Pukagongma/CSP136	Mbuti	-0.002557	-3.443	195554

Table S7. The f4-statistics f4(X, Y; Z, O) supporting Zongri5.1k show connection with UstIshim.

X	Y	Z	O = Mbuti			O = Chimp		
			f4	Z	SNPs	f4	Z	SNPs
Zongri5.1k	AR19K	UstIshim	0.00101	1.78	659,511	0.00093	1.48	632,515
Zongri5.1k	AR14K	UstIshim	0.00134	2.79	530,830	0.00181	3.33	507,414
Zongri5.1k	Mongolia_N_North	UstIshim	0.00127	2.95	860,684	0.00116	2.45	824,913
Zongri5.1k	DevilsCave_N	UstIshim	0.00134	2.83	874,625	0.00156	3.06	838,387
Zongri5.1k	Yumin	UstIshim	0.00154	2.81	755,888	0.00192	3.15	724,067
Zongri5.1k	Bianbian	UstIshim	0.00101	1.88	635,628	0.00148	2.4	608,264
Zongri5.1k	Boshan	UstIshim	0.00108	2.04	819,055	0.00092	1.58	785,252
Zongri5.1k	Xiaogao	UstIshim	0.00127	2.34	791,749	0.00133	2.19	758,815
Zongri5.1k	YR_MN	UstIshim	0.00141	3.15	871,706	0.00108	2.26	835,615
Zongri5.1k	Qihe3	UstIshim	0.00116	2.04	550,415	0.00157	2.44	528,341
Zongri5.1k	Qihe	UstIshim	0.00152	2.49	312,767	0.00079	1.06	299,521
Zongri5.1k	Man_Bac	UstIshim	0.00199	3.63	292,779	0.00257	3.87	279,660

Zongri5.1k	Xitoucun	UstIshim	0.00189	3.9	518,762	0.00204	3.54	497,026

Table S8. F4-statistics showing the relationship of Lajia4k to Early Ancient Tibetans and ancient northern East Asians.

See Excel.

Table S9. F4-statistics testing relationships within Early Ancient Tibetans or relative to world wide present-day and ancient human populations. (A) Z-scores for f4(Ancient Tibetan 1, Ancient Tibetan 2; world wide present-day/ancient, Mbuti) statistics were calculated, where present-day populations include French, Mixe, Onge, Tu, Naxi, Han, Tibetan_Shannna, Sherpa_Shigatse and Atayal and ancient populations include Clovis, Ganj_Dareh_N, Gonur1_BA, AR19k, Bianbian, Yumin, Qihe3, La368, Dushan, Miaozigou_MN and Lajia4k. (B) Z-scores for f4(early ancient Tibetans, diverse lowland populations; early ancient Tibetans, Mbuti) where lowland populations include La368 (Hoabinhian), Qihe3 (southern East Asian), Bianbian, Yumin, YR_MN and Lajia4k (northern East Asians). (C) Z-scores for f4(early ancient Tibetans, select Early Ancient Tibetans; early ancient Tibetans, Mbuti) where the selected populations include Yushu2.8k, Chamdo2.8k_1, Nagqu2.7k, Shannan3k and Lubrak. (D) Z-scores and f4 values for f4(select Early Ancient Tibetans, Ngari2.3k/Ngari1.1k; ancient Central and South Asians, Mbuti) where the selected populations include Yushu2.8k, Chamdo2.8k_1, Nagqu2.7k, Shannan3k and Lubrak.

See Excel.

Table S10. Top 30 significantly negative admixture-f_3 pairs for Zongri4.5k, Ngari2.3k, Nyingchi2k, Shannan1.3k, Shigatse0.9k, Shigatse0.7k.

See Excel.

Table S11. Significant admixture-f_3 pairs for present-day plateau populations. Only the top 30 pairs are shown.

Ref1	Ref2	Test	f_3	std	Z	nSNPs
Shigatse0.7k	Vt719_G3_1	Tibetan_Chamdo	-0.004576	0.001428	-3.205	155421
Shigatse2.1k	HMMH_MN	Tibetan_Nagqu	-0.005548	0.001556	-3.567	347538
Lhasa1k	Dacaozi1.9k	Tibetan_Nagqu	-0.004385	0.001344	-3.263	530491

Shannan2.2k_1	Nomad_Med_TS	Tibetan_Nagqu	-0.003514	0.001154	-3.044	431624
Shigatse2.1k	HMMH_MN	Tibetan_Shannan	-0.00584	0.001241	-4.706	391133
Shannan2.2k_2	HMMH_MN	Tibetan_Shannan	-0.005502	0.001809	-3.041	189593
Shannan1.3k	Nomad_IA_TS	Tibetan_Shannan	-0.005251	0.001225	-4.287	588804
Shannan1.3k	WLR_BA	Tibetan_Shannan	-0.005124	0.001318	-3.888	327667
Shannan1.3k	Miaozigou_MN	Tibetan_Shannan	-0.004975	0.001582	-3.144	177027
Shannan2.2k_2	DevilsCave_N	Tibetan_Shannan	-0.004825	0.001287	-3.747	322909
Shannan2.2k_2	Shimao_LN	Tibetan_Shannan	-0.004818	0.001285	-3.748	318124
Shannan2.2k_1	YR_MN	Tibetan_Shannan	-0.004635	0.000911	-5.085	454439
Shannan2.2k_1	Ikawazu	Tibetan_Shannan	-0.00432	0.001424	-3.034	349806
Shannan2.2k_1	WLR_BA	Tibetan_Shannan	-0.004233	0.001166	-3.631	259717
Shannan2.2k_1	Kangju_TS	Tibetan_Shannan	-0.004194	0.001118	-3.752	381653
Shannan1.3k	Kangju_TS	Tibetan_Shannan	-0.004174	0.001221	-3.419	491907
Shannan2.2k_1	Shimao_LN	Tibetan_Shannan	-0.004168	0.000954	-4.37	448511
Shannan1.3k	YR_3100BP	Tibetan_Shannan	-0.004117	0.001168	-3.526	535928
Shannan1.3k	Dacaozi1.9k	Tibetan_Shannan	-0.004097	0.001071	-3.827	565643
Shannan2.2k_1	Nomad_Med_TS	Tibetan_Shannan	-0.004023	0.000825	-4.879	466715
Shannan1.3k	Karluk_TS	Tibetan_Shannan	-0.00402	0.001183	-3.398	591274
Shannan2.2k_1	Dacaozi1.9k	Tibetan_Shannan	-0.003969	0.000936	-4.242	440441
Shannan2.2k_1	Karluk_TS	Tibetan_Shannan	-0.003961	0.001001	-3.955	458926
Shannan2.2k_1	Nomad_IA_TS	Tibetan_Shannan	-0.003772	0.001091	-3.456	457033
Shannan2.2k_1	DevilsCave_N	Tibetan_Shannan	-0.003714	0.001021	-3.638	455841
Shannan2.2k_1	YR_3100BP	Tibetan_Shannan	-0.003714	0.001063	-3.493	418372
Shannan2.2k_2	YR_MN	Tibetan_Shannan	-0.003647	0.001161	-3.141	322365
Lubrak	Kangju_TS	Tibetan_Shannan	-0.003583	0.001178	-3.041	418161
Shannan1.3k	TianShanSaka_TS	Tibetan_Shannan	-0.003504	0.000973	-3.603	629852
Shannan2.2k_1	YR_2200BP	Tibetan_Shannan	-0.003478	0.000892	-3.899	445654
Shannan2.2k_2	YR_LN	Tibetan_Shannan	-0.003394	0.000943	-3.598	332723
Shannan2.2k_1	Lajia4k	Tibetan_Shannan	-0.003137	0.000895	-3.503	450019
Shannan2.2k_1	Wusun_TS	Tibetan_Shannan	-0.003101	0.00103	-3.011	467743
Shannan1.3k	Nomad_Med_TS	Tibetan_Shannan	-0.00308	0.000897	-3.435	601708
Shigatse2.1k	HMMH_MN	Tibetan_Lhasa	-0.007371	0.001501	-4.911	347731
Shannan2.2k_1	Kangju_TS	Tibetan_Lhasa	-0.007001	0.001379	-5.078	345249
Shannan2.2k_1	Karluk_TS	Tibetan_Lhasa	-0.006595	0.00129	-5.114	415526
Shannan2.2k_1	La_G2	Tibetan_Lhasa	-0.006433	0.001887	-3.409	129858
Shigatse1.9k	G6	Tibetan_Lhasa	-0.00638	0.001836	-3.476	237245
Shannan2.2k_1	Ikawazu	Tibetan_Lhasa	-0.00637	0.001656	-3.848	309892
Shannan2.2k_1	DevilsCave_N	Tibetan_Lhasa	-0.006175	0.001268	-4.868	411830
Shannan2.2k_2	DevilsCave_N	Tibetan_Lhasa	-0.00615	0.001636	-3.758	289913
Lubrak	Kangju_TS	Tibetan_Lhasa	-0.006149	0.001458	-4.217	377574
Shannan2.2k_1	G6	Tibetan_Lhasa	-0.006111	0.001619	-3.776	191908
Shannan2.2k_1	YR_MN	Tibetan_Lhasa	-0.005867	0.001174	-4.997	411901
Shigatse1.2k	Dacaozi1.9k	Tibetan_Lhasa	-0.005741	0.001872	-3.067	87558
Shannan2.2k_1	Nomad_Med_TS	Tibetan_Lhasa	-0.005677	0.001063	-5.341	432006
Shannan2.2k_1	Nomad_IA_TS	Tibetan_Lhasa	-0.005532	0.00136	-4.068	414156
Shannan2.2k_1	G4	Tibetan_Lhasa	-0.005489	0.00142	-3.865	225030
Lubrak	Mongolia_EBA_2_Chemurchek	Tibetan_Lhasa	-0.005486	0.001486	-3.693	418410
Shigatse1.9k	DevilsCave_N	Tibetan_Lhasa	-0.005416	0.001589	-3.408	506279
Shigatse1.9k	Karluk_TS	Tibetan_Lhasa	-0.005403	0.001533	-3.525	510916
Shannan1.3k	Nomad_IA_TS	Tibetan_Lhasa	-0.005376	0.001545	-3.479	530498
Shannan2.2k_1	YR_3100BP	Tibetan_Lhasa	-0.005374	0.001283	-4.188	373326
Shigatse2.1k	TianShanHun_TS	Tibetan_Lhasa	-0.005316	0.001014	-5.24	747809
Shigatse2.1k	Karluk_TS	Tibetan_Lhasa	-0.005265	0.00119	-4.423	611553
Shannan1.3k	Kangju_TS	Tibetan_Lhasa	-0.005259	0.001555	-3.382	442118
Shannan2.2k_1	Dacaozi1.9k	Tibetan_Lhasa	-0.005212	0.001201	-4.339	396636
Shannan2.2k_1	TianShanHun_TS	Tibetan_Lhasa	-0.005197	0.001045	-4.972	512410
Shannan2.2k_1	Wusun_TS	Tibetan_Lhasa	-0.005187	0.001264	-4.103	429413
Shannan2.2k_1	Okunevo_EMBA	Tibetan_Lhasa	-0.005154	0.001076	-4.792	475164
Shigatse2.1k	Okunevo_EMBA	Tibetan_Lhasa	-0.005134	0.001036	-4.955	694969
Shigatse2.1k	Kangju_TS	Tibetan_Lhasa	-0.005123	0.001309	-3.913	508480
Lubrak	Wusun_TS	Tibetan_Lhasa	-0.005104	0.00138	-3.698	470220
Shigatse2.1k	HMMH_MN	Tibetan_Shigatse	-0.006636	0.001137	-5.836	413311
Shannan2.2k_2	HMMH_MN	Tibetan_Shigatse	-0.005539	0.001778	-3.115	201009
Shannan2.2k_2	DevilsCave_N	Tibetan_Shigatse	-0.004983	0.001213	-4.108	339748
Shannan2.2k_1	Karluk_TS	Tibetan_Shigatse	-0.004964	0.000953	-5.209	481106
Lubrak	Kangju_TS	Tibetan_Shigatse	-0.004872	0.001085	-4.489	438297
Shannan2.2k_1	Kangju_TS	Tibetan_Shigatse	-0.004647	0.00106	-4.384	399846

Shannan2.2k_1	Ikawazu	Tibetan_Shigatse	-0.004605	0.001314	-3.504	369662
Shannan1.3k	Nomad_IA_TS	Tibetan_Shigatse	-0.004546	0.001163	-3.91	617859
Shigatse2.1k	Karluk_TS	Tibetan_Shigatse	-0.004332	0.000843	-5.137	702232
Shigatse2.1k	AR_Xianbei_IA	Tibetan_Shigatse	-0.004289	0.001239	-3.463	220582
Shannan2.2k_1	YR_MN	Tibetan_Shigatse	-0.004261	0.000836	-5.095	477562
Shannan1.3k	WLR_BA	Tibetan_Shigatse	-0.004151	0.001252	-3.316	347414
Shannan1.3k	Karluk_TS	Tibetan_Shigatse	-0.004081	0.001134	-3.599	621085
Shannan2.2k_1	WLR_BA	Tibetan_Shigatse	-0.00403	0.001133	-3.557	274593
Shannan1.3k	Kangju_TS	Tibetan_Shigatse	-0.004008	0.001159	-3.457	516437
Shannan2.2k_2	Shimao_LN	Tibetan_Shigatse	-0.003933	0.001223	-3.215	335667
Shigatse2.1k	TianShanHun_TS	Tibetan_Shigatse	-0.003876	0.000626	-6.191	775793
Lubrak	Wusun_TS	Tibetan_Shigatse	-0.003869	0.000955	-4.05	534645
Shannan2.2k_1	DevilsCave_N	Tibetan_Shigatse	-0.003843	0.000947	-4.057	478975
Shannan2.2k_1	Nomad_IA_TS	Tibetan_Shigatse	-0.003836	0.001036	-3.704	478545
Shigatse2.1k	Wusun_TS	Tibetan_Shigatse	-0.00383	0.000815	-4.701	711403
Shigatse2.1k	Kangju_TS	Tibetan_Shigatse	-0.003787	0.00096	-3.947	584125
Shannan2.2k_1	Nomad_Med_TS	Tibetan_Shigatse	-0.003785	0.000729	-5.193	485788
Shannan2.2k_1	G4	Tibetan_Shigatse	-0.003774	0.001153	-3.272	268044
Shigatse2.1k	Ma912_G2	Tibetan_Shigatse	-0.003712	0.001044	-3.556	549831
Shannan2.2k_2	Lajia4k	Tibetan_Shigatse	-0.003602	0.001082	-3.329	335418
Shannan2.2k_2	YR_MN	Tibetan_Shigatse	-0.003505	0.001101	-3.184	339072
Nagqu1.4k	Ma912_G2	Tibetan_Shigatse	-0.003387	0.001035	-3.271	562443
Shannan2.2k_1	Shimao_LN	Tibetan_Shigatse	-0.003304	0.000895	-3.691	472751
Shannan2.2k_1	Wusun_TS	Tibetan_Shigatse	-0.003297	0.000952	-3.462	487461
Lubrak	Kangju_TS	Sherpa_Shigatse	-0.00466	0.001271	-3.665	406930

Table S12. Z-scores for f_4-statistics showing that Early Ancient Tibetans of the south-southwest cluster share more alleles with each other than populations of other plateau regions.

	D (X, Chamdo2.8k_1; Z, Mbuti)						D (X, Nagqu2.7k; Z, Mbuti)						D (X, Yushu2.8k; Z, Mbuti)					
X/Z	Suila	Lubrak	Chokhopani	Rhirhi	Shigatse2.6k	Shannan3k	Suila	Lubrak	Chokhopani	Rhirhi	Shigatse2.6k	Shannan3k	Suila	Lubrak	Chokhopani	Rhirhi	Shigatse2.6k	Shannan3k
Suila		4.8	3.6	3.3	4.6	1.7		4.2	4.3	2.8	2.8	3.1		8.5	6.5	5.6	7	4.7
Lubrak	6.2		5.4	8.5	5.7	3.2	6.1		6.8	8.9	5	4.5	8.8		11	13	9.5	6.8
Chokhopani	3.7	2.9		4.7	2.3	0.2	2.6	2.1		4.1	0.6	1	4.8	7.9		7.4	5.1	2.6
Rhirhi	3.5	6.1	5.3		2.6	0.6	2.5	5.1	5.6		1.4	2	4.7	12	9		6.1	4.1
Shigatse2.6k	6.1	5.5	4.4	4.8		1.5	4.8	4.7	4.9	4.7		2.6	6.8	9.3	7.4	7.6		4.1
Shannan3k	3.9	3.8	2.5	2.9	2.2		3.4	2.7	2.9	3.4	1.5		5.8	8	5	6.1	4.9	

Table S13. F_4-statistics showing 1,600-1,100-year-old Nagqu individuals cluster with 2,100-year-old Shigatse population relative to 2700-2500 BP Nagqu individuals.

X	Y	Z	O	f_4	Z	SNPs	Interpretation
Nagqu1.1k	Nagqu2.5k	Shigatse2.1k	Mbuti	0.0025	5.0	526127	Nagqu1.1k forms a chade with Shigatse2.1k relative to Nagqu2.5k
Nagqu1.1k	Shigatse2.1k	Nagqu2.5k	Mbuti	0.0009	1.7	526127	
Nagqu2.5k	Shigatse2.1k	Nagqu1.1k	Mbuti	-0.0017	-3.1	526127	
Nagqu1.1k	Nagqu2.7k	Shigatse2.1k	Mbuti	0.0019	3.8	518170	

Nagqu1.1k	Shigatse2.1k	Nagqu2.7k	Mbuti	0.0000	0.1	518170	Nagqu1.1k forms a chade with Shigatse2.1k relative to Nagqu2.7k
Nagqu2.7k	Shigatse2.1k	Nagqu1.1k	Mbuti	-0.0019	-3.4	518170	
Nagqu1.6k	Nagqu2.5k	Shigatse2.1k	Mbuti	0.0019	3.9	608825	Nagqu1.6k forms a chade with Shigatse2.1k relative to Nagqu2.5k
Nagqu1.6k	Shigatse2.1k	Nagqu2.5k	Mbuti	-0.0005	-1.0	608825	
Nagqu2.5k	Shigatse2.1k	Nagqu1.6k	Mbuti	-0.0025	-4.7	608825	
Nagqu1.6k	Nagqu2.7k	Shigatse2.1k	Mbuti	0.0010	2.0	627089	Nagqu1.6k forms a chade with Shigatse2.1k relative to Nagqu2.7k
Nagqu1.6k	Shigatse2.1k	Nagqu2.7k	Mbuti	-0.0005	-1.0	627089	
Nagqu2.7k	Shigatse2.1k	Nagqu1.6k	Mbuti	-0.0015	-3.0	627089	
Nagqu1.4k	Nagqu2.5k	Shigatse2.1k	Mbuti	0.0033	8.2	653176	Nagqu1.4k forms a chade with Shigatse2.1k relative to Nagqu2.5k
Nagqu1.4k	Shigatse2.1k	Nagqu2.5k	Mbuti	0.0005	1.3	653176	
Nagqu2.5k	Shigatse2.1k	Nagqu1.4k	Mbuti	-0.0028	-6.7	653176	
Nagqu1.4k	Nagqu2.7k	Shigatse2.1k	Mbuti	0.0026	5.9	672375	Nagqu1.4k forms a chade with Shigatse2.1k relative to Nagqu2.7k
Nagqu1.4k	Shigatse2.1k	Nagqu2.7k	Mbuti	-0.0002	-0.4	672375	
Nagqu2.7k	Shigatse2.1k	Nagqu1.4k	Mbuti	-0.0027	-6.3	672375	

Table S14. Distal *qpAdm* models for Early Ancient Tibetans. p# refers to mixture proportions, std# refers to the standard error corresponding to each mixture proportion. The analysis was performed with the 'rotating' scheme using the 'R15' reference set. (A) Feasible models for each target population. (B) Informative non-fitting models to facilitate comparisons across target populations. The models could be rejected due to low *P* values ($P < 0.05$), or that not all confidence interval of estimated proportions fall within 0 to 1 range.
See Excel.

Table S15. Proximal *qpAdm* models for Early Ancient Tibetans. p# refers to mixture proportions, std# refers to the standard error corresponding to each mixture proportion. (A) Feasible or best-fitting models for each target population. *P*-values smaller than 0.05 are highlighted. (B) Informative non-fitting models to facilitate comparisons across target populations. The models could be rejected due to low P values ($P < 0.05$), or that not all confidence interval of estimated proportions fall within 0 to 1 range. The qpAdm models were obtained through a rotating scheme, the "R15 + Zongri5.1k" outgroup set were used for modeling populations older than 4,000 BP (i.e. from the Zongri archaeological site). Both "R15 + Lajia4k + Zongri5.1k" and "R15 + Lajia4k + Lubrak" sets were used for modeling Early Ancient Tibetans younger than 3,000 BP.
See Excel.

Table S16. *qpWave* analysis for 2700-1900 BP Tibetan Plateau populations. Outgroup population with power to distinguish different plateau ancestries (‘O10’) were used as the pright population. Blue indicates $P > 0.05$ whereas red indicates $P < 0.01$.

	Yushu2.8k	Chamdo2.8k_1	Chamdo2.8k_2	Chamdo2.7k	Nagqu2.7k	Nagqu2.5k	Shannan3k	Shannan2.2k_1	Shannan2.2k_2	Shigatse1.9k	Shigatse2.1k	Shigatse2.6k	Lubrak	Chokhopani	Rhirhi
Yushu2.8k		3.1E-03	2.6E-03	1.3E-01	8.3E-01	4.1E-01	1.6E-10	7.2E-14	2.7E-13	1.5E-10	4.3E-15	2.6E-17	1.6E-18	1.1E-14	1.0E-12
Chamdo2.8k_1	3.1E-03		1.5E-02	5.0E-01	8.3E-03	2.0E-01	8.1E-04	1.0E-05	1.2E-04	1.4E-06	6.5E-09	1.8E-10	6.9E-11	9.6E-07	1.1E-07
Chamdo2.8k_2	2.6E-03	1.5E-02		1.4E-01	6.2E-01	4.5E-01	4.2E-01	6.5E-02	1.6E-01	1.0E-02	3.0E-02	7.4E-04	7.3E-07	1.3E-02	4.9E-02
Chamdo2.7k	1.3E-01	5.0E-01	1.4E-01		2.3E-01	4.2E-01	8.1E-03	9.9E-03	5.7E-06	9.7E-03	7.1E-05	5.3E-06	9.7E-08	1.3E-04	2.8E-04
Nagqu2.7k	8.3E-01	8.3E-03	6.2E-01	2.3E-01		7.3E-01	8.4E-04	2.4E-03	5.0E-05	1.3E-04	5.9E-07	1.5E-06	1.9E-10	6.8E-06	1.1E-04
Nagqu2.5k	4.1E-01	2.0E-01	4.5E-01	4.2E-01	7.3E-01		1.5E-03	1.3E-07	1.4E-05	2.0E-06	1.2E-08	1.3E-09	4.8E-12	1.4E-05	2.1E-05
Shannan3k	1.6E-10	8.1E-04	4.2E-01	8.1E-03	8.4E-04	1.5E-03		5.3E-01	1.7E-01	3.6E-01	2.6E-01	1.0E-01	1.7E-01	1.0E+00	7.8E-01
Shannan2.2k_1	7.2E-14	1.0E-05	6.5E-02	9.9E-03	2.4E-03	1.3E-07	5.3E-01		2.2E-01	5.9E-01	3.2E-01	1.7E-01	3.8E-02	1.4E-02	3.7E-02
Shannan2.2k_2	2.7E-13	1.2E-04	1.6E-01	5.7E-06	5.0E-05	1.4E-05	1.7E-01	2.2E-01		5.2E-03	6.9E-04	1.9E-03	2.3E-05	1.5E-04	2.9E-02
Shigatse1.9k	1.5E-10	1.4E-06	1.0E-02	9.7E-03	1.3E-04	2.0E-06	3.6E-01	5.9E-01	5.2E-03		7.8E-01	4.7E-01	8.1E-01	2.4E-02	6.8E-02
Shigatse2.1k	4.3E-15	6.5E-09	3.0E-02	7.1E-05	5.9E-07	1.2E-08	2.6E-01	3.2E-01	6.9E-04	7.8E-01		2.3E-01	5.2E-02	5.4E-03	5.0E-03
Shigatse2.6k	2.6E-17	1.8E-10	7.4E-04	5.3E-06	1.5E-06	1.3E-09	1.0E-01	1.7E-01	1.9E-03	4.7E-01	2.3E-01		6.4E-02	5.0E-03	5.4E-04
Lubrak	1.6E-18	6.9E-11	7.3E-07	9.7E-08	1.9E-10	4.8E-12	1.7E-01	3.8E-02	2.3E-05	8.1E-01	5.2E-02	6.4E-02		3.1E-04	2.3E-03
Chokhopani	1.1E-14	9.6E-07	1.3E-02	1.3E-04	6.8E-06	1.4E-05	1.0E+00	1.4E-02	1.5E-04	2.4E-02	5.4E-03	5.0E-03	3.1E-04		4.7E-01
Rhirhi	1.0E-12	1.1E-07	4.9E-02	2.8E-04	1.1E-04	2.1E-05	7.8E-01	3.7E-02	2.9E-02	6.8E-02	5.0E-03	5.4E-04	2.3E-03	4.7E-01	

Table S17. Feasible proximal *qpAdm* models for ancient and present-day populations younger than 2,800 BP. (A) Feasible or the best-fitting models are shown for each target population. A feasible model means that the target can be described using the given mixture model (pvalue > 0.05), the mixture model for the given number of sources (n) is better than a mixture model of n-1 sources, and all mixture proportions (p#) are greater than zero. Models with pvalue < 0.05 are highlighted in yellow. std# refers to the standard error corresponding to each mixture proportion. (B) Informative non-fitting models to facilitate comparisons across target populations. The models could be rejected due to low *P* values ($P < 0.05$), or that not all confidence interval of estimated proportions fall within 0 to 1 range. Outgroup population with power to distinguish different plateau ancestries (‘O10’) were used as the pright population.

See Excel.

Table S18. Z scores for f_4 (X, Y; Neanderthal/Denisovan, Chimp) testing symmetry between ancient and present-day populations with respect to archaic humans. Blue highlighting indiciates Z<-3, whereas red highlighting indicates Z>3.

X/Y	Z = Neanderthal						Z = Denisovan					
	Han	Ami	Daur	Tibetan_Shannan	French	Papuan	Han	Ami	Daur	Tibetan_Shannan	French	Papuan
**Zongri5.1k**	1.6	1.7	1.7	2.1	2	-0.2	1.7	2	1.5	1.4	1.1	-2.9
**Yushu2.8k**	0.5	0.5	0.3	0.7	0.7	-1.4	1.2	1.3	0.8	0.8	0.4	-4.2
**Yushu0.5k**	-0.7	-0.5	-0.6	-0.5	-0.3	-1.6	-1.3	-1.1	-1.5	-1.5	-1.9	-4.4
**Chamdo2.8k_1**	1	1.1	1	1.3	1.3	-0.7	0.4	0.9	-0.1	0.1	-0.4	-3.9
**Chamdo2.8k_2**	1.8	2	2	2.1	1.8	0.7	0.8	1.2	0.5	0.6	0	-2.6
**Chamdo2.7k**	-0.9	-0.5	-0.7	-0.6	-0.9	-2.3	0.1	0.4	0	0.1	-0.7	-3.2
**Nagqu2.7k**	0.1	0.1	-0.1	0.3	0.2	-1.5	0.9	0.8	0.5	0.5	0	-3.5
**Nagqu2.5k**	0.2	0.6	-0.2	0.1	0.5	-1.4	0.7	1.2	0.4	0.4	0.5	-3
**Nagqu1.6k**	-0.9	-0.7	-0.9	-0.5	-0.6	-2.6	1	1.1	0.7	0.9	0.1	-3.8
**Nagqu1.4k**	-0.5	0	-0.5	0.2	0.1	-2.3	-0.6	0	-0.9	-0.7	-1.3	-6
**Nagqu1.1k**	-0.4	0.1	-0.7	-0.2	-0.3	-1.7	0.4	0.7	0	0.1	-0.2	-3.4
**Nyingchi2k**	-0.9	-0.4	-1	-0.3	-0.7	-2.8	1	1.1	0.5	0.7	0	-4.9
**Nyingchi2k_o**	-0.2	-0.1	-0.1	0	0.2	-1.4	0.6	0.7	0.3	0.3	-0.2	-3
**Nyingchi0.8k**	0.3	0.3	0.3	0.8	0.6	-1.7	0.1	0.1	-0.2	-0.2	-0.4	-4.4
**Nyingchi0.1k**	-0.9	-0.2	-1.5	-1	-1.5	-1	-1.3	-1.4	-1.5	-1.4	-1.4	-1.9
**Lhasa1k**	0.1	0.2	0.1	0.7	0.5	-1.6	-0.5	-0.5	-0.8	-0.8	-1.2	-5.1
**Lhasa1.1k**	-0.8	-0.4	-0.4	-0.5	-0.3	-1.9	-0.8	-0.4	-0.6	-1.2	-0.8	-3.5
**Lhasa0.7k**	1.1	1.3	1.2	1.8	1.8	0	2	2.2	1.8	1.9	1.9	-2
**Shannan3k**	-1	-1	-0.9	-0.5	-0.8	-2.8	-1	-0.9	-1	-1.2	-1.8	-5.1
**Shannan2.2k_2**	-0.2	0.2	-0.3	-0.1	-0.2	-1.6	0.4	0.7	0	0	-0.5	-3.9
**Shannan2.2k_1**	-0.1	0	-0.1	0.2	0.2	-1.9	1.2	0.8	0.9	0.8	0.5	-3.6
**Shannan1.3k**	0.5	0.7	0.6	1.1	1	-0.9	1.2	1.6	1.1	1.2	0.7	-3.2
**Shigatse2.6k**	0.8	0.8	0.9	1.7	1.3	-1.1	1.3	1.5	1.1	1.4	0.7	-3.5
**Shigatse2.1k**	1.1	1.2	1	1.7	1.4	-1.1	1.1	1.2	0.8	0.9	0.3	-4.9
**Shigatse1.9k**	-0.2	0	-0.3	0.2	0	-1.8	1	1.1	0.7	0.8	0.1	-3.9
**Shigatse1.5k_2**	-0.9	-0.4	-0.7	-0.4	-0.5	-2.2	-0.2	0.1	0.1	0.3	-0.2	-2.5
**Shigatse1.5k_1**	-0.7	-0.5	-0.7	-0.1	-0.4	-2.3	0.3	0.3	-0.2	0.1	-0.3	-4.1
**Shigatse1.2k**	0.1	-0.6	0.6	0.5	1	-0.1	1.2	1.1	0.8	1.6	1.2	-0.8
**Shigatse0.9k**	2.5	2.2	2.1	3.2	2.1	-1.2	3.5	2.9	2.7	3	1.5	-5.2
**Shigatse0.7k**	-0.4	-0.2	-0.4	0.3	0	-2.3	0.5	0.7	0.2	0.3	-0.3	-5.5
**Ngari2.3k**	-1	-1	-1.2	-0.4	-0.5	-2.6	0.2	0.3	-0.3	0	-0.3	-5
**Ngari2.3k_o1**	-1.6	-1	-1.6	-1.1	-1.1	-2.9	-0.2	-0.1	-0.7	-0.6	-0.7	-4.2
**Ngari2.3k_o2**	1.5	1.4	1.5	1.3	1.1	-0.3	0.9	1.2	0.5	0.7	0	-2.4
**Ngari0.1k**	1.1	1.4	1.2	1.5	1.5	-0.6	-0.1	0.3	-0.3	-0.4	-0.8	-4.7

Table S19. 1600BP and 1400-1100BP Nagqu individuals show different affinities with various ancient individuals dating to 2,200-1,900 BP in the southern and southwestern plateau. F_4-statistics are of the form f4 (Pop1, Pop2; Pop3, Mbuti), and a significantly positive Z-score (Z > 3) indicates that Pop3 shares more alleles with Pop1 than Pop2.

X	Y	Z	O	*f*$_4$	Z	SNPs	Interpretation
Shigatse1.9k	Shigatse2.1k	Nagqu1.6k	Mbuti	0.0011	2.0	736381	Nagqu1.6k shares more alleles with Shigatse1.9k than other 2300-1900 BP populations across the Tibetan Plateau.
Shigatse1.9k	Ngari2.3k	Nagqu1.6k	Mbuti	0.0037	6.6	595581	
Shigatse1.9k	Nyingchi2k	Nagqu1.6k	Mbuti	0.0027	4.9	729002	
Shigatse1.9k	Shannan2.2k_1	Nagqu1.6k	Mbuti	0.0017	3.1	582088	
Shigatse1.9k	Shannan2.2k_2	Nagqu1.6k	Mbuti	0.0030	4.3	436523	
Shigatse2.1k	Ngari2.3k	Nagqu1.4k	Mbuti	0.0037	11.1	682099	Nagqu1.4k shares more alleles with Shigatse2.1k than other 2300-1900 BP populations except Shannan2.2k_1 across the Tibetan Plateau.
Shigatse2.1k	Nyingchi2k	Nagqu1.4k	Mbuti	0.0015	4.2	891870	
Shigatse2.1k	Shannan2.2k_2	Nagqu1.4k	Mbuti	0.0014	3.0	481451	
Shigatse2.1k	Shigatse1.9k	Nagqu1.4k	Mbuti	0.0014	3.4	806366	
Shannan2.2k_1	Shannan2.2k_2	Nagqu1.4k	Mbuti	0.0015	3.1	400578	Nagqu1.4k shares more alleles with Shannan2.2k_1 than other 2300-1900 BP populations except Shigatse2.1k across the Tibetan Plateau.
Shannan2.2k_1	Shigatse1.9k	Nagqu1.4k	Mbuti	0.0016	3.7	616591	
Shannan2.2k_1	Ngari2.3k	Nagqu1.4k	Mbuti	0.0037	9.5	540823	
Shannan2.2k_1	Nyingchi2k	Nagqu1.4k	Mbuti	0.0016	4.2	657740	
Shigatse2.1k	Ngari2.3k	Nagqu1.1k	Mbuti	0.0033	7.4	500318	Nagqu1.1k shares more alleles with Shigatse2.1k than other 2300-1900 BP populations across the Tibetan Plateau.
Shigatse2.1k	Nyingchi2k	Nagqu1.1k	Mbuti	0.0011	2.5	606534	
Shigatse2.1k	Shannan2.2k_1	Nagqu1.1k	Mbuti	0.0005	1.1	500142	
Shigatse2.1k	Shannan2.2k_2	Nagqu1.1k	Mbuti	0.0012	2.3	390722	
Shigatse2.1k	Shigatse1.9k	Nagqu1.1k	Mbuti	0.0023	4.2	575976	

Table S20. Archaic ancestry estimates using AdmixFrog. NEA, Neanderthal; DEN, Denisovan.

See Excel.

Table S21. Read counts and base information of ancient Tibetan Plateau individuals at 20 SNPs in *EPAS1* that are highly differentiated between Tibetans and other modern humans. The count cells with only reads supporting the presence of the common modern human allele are shaded in blue, the cells with only reads supporting the common Tibetan allele are shaded in pink, and the cells with reads supporting both alleles are shaded in orange. The p (AA), p (AB) and p (BB) represent the posterior probability of different genotypes where A represents the common modern human allele and B represents the common Tibetan allele. The probability was only called for individuals with sum reads count >= 4.

See Excel.

Table S22. Allele frequency of Denisovan-like *EPAS1* haplotype in present-day Tibetan populations.

Population	# of individuals	Frequency
Tibetan_Chamdo	6	0.75
Tibetan_Lhasa	3	0.83
Tibetan_Nagqu	3	1
Tibetan_Nyingchi	2	0.75
Tibetan_Shannan	7	0.79
Tibetan_Shigatse	12	0.96

Table S23. Counts of reads mapped to functional SNPs in other phenotypic genes. The count cells with only reads supporting the presence of the ancestral allele are shaded in blue, the cells with only reads supporting the derived allele are shaded in pink, and the cells with reads supporting both alleles are shaded in orange.

See Excel.

REFERENCES AND NOTES

1. F. Chen, F. Welker, C. C. Shen, S. E. Bailey, I. Bergmann, S. Davis, H. Xia, H. Wang, R. Fischer, S. E. Freidline, T. L. Yu, M. M. Skinner, S. Stelzer, G. Dong, Q. Fu, G. Dong, J. Wang, D. Zhang, J. J. Hublin, A late middle Pleistocene Denisovan mandible from the Tibetan Plateau. *Nature* **569**, 409–412 (2019).

2. X. L. Zhang, B. B. Ha, S. J. Wang, Z. J. Chen, J. Y. Ge, H. Long, W. He, W. Da, X. M. Nian, M. J. Yi, X. Y. Zhou, P. Q. Zhang, Y. S. Jin, O. Bar-Yosef, J. W. Olsen, X. Gao, The earliest human occupation of the high-altitude Tibetan Plateau 40 thousand to 30 thousand years ago. *Science* **362**, 1049–1051 (2018).

3. F. H. Chen, G. H. Dong, D. J. Zhang, X. Y. Liu, X. Jia, C. B. An, M. M. Ma, Y. W. Xie, L. Barton, X. Y. Ren, Z. J. Zhao, X. H. Wu, M. K. Jones, Agriculture facilitated permanent human occupation of the Tibetan Plateau after 3600 B.P. *Science* **347**, 248–250 (2015).

4. M. C. Meyer, M. S. Aldenderfer, Z. Wang, D. L. Hoffmann, J. A. Dahl, D. Degering, W. R. Haas, F. Schlutz, Permanent human occupation of the central Tibetan Plateau in the early Holocene. *Science* **355**, 64–67 (2017).

5. M. Aldenderfer, Peopling the Tibetan Plateau: Insights from archaeology. *High Alt. Med. Biol.* **12**, 141–147 (2011).

6. D. Lu, H. Lou, K. Yuan, X. Wang, Y. Wang, C. Zhang, Y. Lu, X. Yang, L. Deng, Y. Zhou, Q. Feng, Y. Hu, Q. Ding, Y. Yang, S. Li, L. Jin, Y. Guan, B. Su, L. Kang, S. Xu, Ancestral origins and genetic history of Tibetan highlanders. *Am. J. Hum. Genet.* **99**, 580–594 (2016).

7. C.-C. Wang, H.-Y. Yeh, A. N. Popov, H.-Q. Zhang, H. Matsumura, K. Sirak, O. Cheronet, A. Kovalev, N. Rohland, A. M. Kim, S. Mallick, R. Bernardos, D. Tumen, J. Zhao, Y.-C. Liu, J.-Y. Liu, M. Mah, K. Wang, Z. Zhang, N. Adamski, N. Broomandkhoshbacht, K. Callan, F. Candilio, K. S. D. Carlson, B. J. Culleton, L. Eccles, S. Freilich, D. Keating, A. M. Lawson, K. Mandl, M. Michel, J. Oppenheimer, K. T. Ozdogan, K. Stewardson, S. Wen, S. Yan, F. Zalzala, R. Chuang, C.-J. Huang, H. Looh, C.-C. Shiung, Y. G. Nikitin, A. V. Tabarev, A. A. Tishkin, S. Lin, Z.-Y. Sun, X.-M. Wu, T.-L. Yang, X. Hu, L. Chen, H. Du, J. Bayarsaikhan,

E. Mijiddorj, D. Erdenebaatar, T.-O. Iderkhangai, E. Myagmar, H. Kanzawa-Kiriyama, M. Nishino, K.-I. Shinoda, O. A. Shubina, J. Guo, W. Cai, Q. Deng, L. Kang, D. Li, D. Li, R. Lin, Nini, R. Shrestha, L.-X. Wang, L. Wei, G. Xie, H. Yao, M. Zhang, G. He, X. Yang, R. Hu, M. Robbeets, S. Schiffels, D. J. Kennett, L. Jin, H. Li, J. Krause, R. Pinhasi, D. Reich, Genomic insights into the formation of human populations in East Asia. *Nature* **591**, 413–419 (2021).

8. C. Jeong, A. T. Ozga, D. B. Witonsky, H. Malmstrom, H. Edlund, C. A. Hofman, R. W. Hagan, M. Jakobsson, C. M. Lewis, M. S. Aldenderfer, A. Di Rienzo, C. Warinner, Long-term genetic stability and a high-altitude east Asian origin for the peoples of the high valleys of the Himalayan arc. *Proc. Natl. Acad. Sci. U.S.A.* **113**, 7485–7490 (2016).

9. C.-C. Liu, D. Witonsky, A. Gosling, J. H. Lee, H. Ringbauer, R. Hagan, N. Patel, R. Stahl, J. Novembre, M. Aldenderfer, C. Warinner, A. Di Rienzo, C. Jeong, Ancient genomes from the Himalayas illuminate the genetic history of Tibetans and their Tibeto-Burman speaking neighbors. *Nat. Commun.* **13**, 1203 (2022).

10. R. Czai, *Tibetan Historical Manuscripts* (Gansu People Publishing Company, 2010).

11. J. d'Alpoim Guedes, M. Aldenderfer, The archaeology of the early Tibetan Plateau: New research on the initial peopling through the early bronze age. *J. Archaeol. Res.* **28**, 339–392 (2020).

12. M. Lipatov, K. Sanjeev, R. Patro, K. Veeramah, Maximum likelihood estimation of biological relatedness from low coverage sequencing data. bioRxiv 10.1101/023374 [**Preprint**]. 29 July 2015. https://doi.org/10.1101/023374.

13. A. L. Price, N. J. Patterson, R. M. Plenge, M. E. Weinblatt, N. A. Shadick, D. Reich, Principal components analysis corrects for stratification in genome-wide association studies. *Nat. Genet.* **38**, 904–909 (2006).

14. N. Patterson, P. Moorjani, Y. Luo, S. Mallick, N. Rohland, Y. Zhan, T. Genschoreck, T. Webster, D. Reich, Ancient admixture in human history. *Genetics* **192**, 1065–1093 (2012).

15. J. K. Pickrell, J. K. Pritchard, Inference of population splits and mixtures from genome-wide allele frequency data. *PLOS Genet.* **8**, e1002967 (2012).

16. W. Haak, I. Lazaridis, N. Patterson, N. Rohland, S. Mallick, B. Llamas, G. Brandt, S. Nordenfelt, E. Harney, K. Stewardson, Q. Fu, A. Mittnik, E. Banffy, C. Economou, M. Francken, S. Friederich, R. G. Pena, F. Hallgren, V. Khartanovich, A. Khokhlov, M. Kunst, P. Kuznetsov, H. Meller, O. Mochalov, V. Moiseyev, N. Nicklisch, S. L. Pichler, R. Risch, M. A. Rojo Guerra, C. Roth, A. Szecsenyi-Nagy, J. Wahl, M. Meyer, J. Krause, D. Brown, D. Anthony, A. Cooper, K. W. Alt, D. Reich, Massive migration from the steppe was a source for Indo-European languages in Europe. *Nature* **522**, 207–211 (2015).

17. É. Harney, N. Patterson, D. Reich, J. Wakeley, Assessing the performance of qpAdm: A statistical tool for studying population admixture. *Genetics* **217**, iyaa045 (2021).

18. I. Lazaridis, A. Mittnik, N. Patterson, S. Mallick, N. Rohland, S. Pfrengle, A. Furtwangler, A. Peltzer, C. Posth, A. Vasilakis, P. J. P. McGeorge, E. Konsolaki-Yannopoulou, G. Korres, H. Martlew, M. Michalodimitrakis, M. Ozsait, N. Ozsait, A. Papathanasiou, M. Richards, S. A. Roodenberg, Y. Tzedakis, R. Arnott, D. M. Fernandes, J. R. Hughey, D. M. Lotakis, P. A. Navas, Y. Maniatis, J. A. Stamatoyannopoulos, K. Stewardson, P. Stockhammer, R. Pinhasi, D. Reich, J. Krause, G. Stamatoyannopoulos, Genetic origins of the Minoans and Mycenaeans. *Nature* **548**, 214–218 (2017).

19. G. He, M. Wang, X. Zou, P. Chen, Z. Wang, Y. Liu, H. Yao, L.-H. Wei, R. Tang, C.-C. Wang, H.-Y. Yeh, Peopling history of the Tibetan Plateau and multiple waves of admixture of Tibetans inferred from both ancient and modern genome-wide data. *Front. Genet.* **12**, 725243 (2021).

20. N. Patterson, A. L Price, D. Reich, Population structure and eigenanalysis. *PLOS Genet.* **2**, e190 (2006).

21. M. Raghavan, P. Skoglund, K. E. Graf, M. Metspalu, A. Albrechtsen, I. Moltke, S. Rasmussen, T. W. Stafford, Jr., L. Orlando, E. Metspalu, M. Karmin, K. Tambets, S. Rootsi, R. Magi, P. F. Campos, E. Balanovska, O. Balanovsky, E. Khusnutdinova, S. Litvinov, L. P.

Osipova, S. A. Fedorova, M. I. Voevoda, M. DeGiorgio, T. Sicheritz-Ponten, S. Brunak, S. Demeshchenko, T. Kivisild, R. Villems, R. Nielsen, M. Jakobsson, E. Willerslev, Upper Palaeolithic Siberian genome reveals dual ancestry of native Americans. *Nature* **505**, 87–91 (2014).

22. C. Jeong, B. M. Peter, B. Basnyat, M. Neupane, C. M. Beall, G. Childs, S. R. Craig, J. Novembre, A. Di Rienzo, A longitudinal cline characterizes the genetic structure of human populations in the Tibetan Plateau. *PLOS ONE* **12**, e0175885 (2017).

23. H. H. Chen, G. S. Wang, D. Z. Mei, N. Suo, Brief report on the excavation of Zongri site, Tongde County, Qinghai. *Archaeology (Chinese)* 1–14 (1998).

24. L. L. Ren, G. H. Dong, F. W. Liu, J. d'Alpoim-Guedes, R. K. Flad, M. M. Ma, H. M. Li, Y. S. Yang, Y. J. Liu, D. J. Zhang, G. L. Li, J. Y. Li, F. H. Chen, Foraging and farming: Archaeobotanical and zooarchaeological evidence for Neolithic exchange on the Tibetan Plateau. *Antiquity* **94**, 637–652 (2020).

25. L. Y. Hung, J. F. Cui, H. H. Chen, Emergence of Neolithic communities on the northeastern Tibetan Plateau: Evidence from the Zongri cultural sites, in *BAR International Series 2679* (The 'Crescent -Shaped Cultural Communication Belt': Tong Enzheng's Model in Retrospect: An Examination of Methodological, Theoretical and Material Concerns of Long-Distance Interactions in East Asia, Archaeopress, 2014), pp. 65–78.

26. D. H. Alexander, J. Novembre, K. Lange, Fast model-based estimation of ancestry in unrelated individuals. *Genome Res.* **19**, 1655–1664 (2009).

27. A. M. Hudson, J. W. Olsen, J. Quade, G. Lei, T. E. Huth, H. Zhang, A regional record of expanded Holocene wetlands and prehistoric human occupation from paleowetland deposits of the western Yarlung Tsangpo valley, southern Tibetan Plateau. *Quatern. Res.* **86**, 13–33 (2016).

28. Z. Ling, X. Yang, Y. Wang, Y. Wang, J. Jin, D. Zhang, F. Chen, OSL chronology of the Liena archeological site in the Yarlung Tsangpo valley throws new light on human occupation of the Tibetan Plateau. *The Holocene* **30**, 1043–1052 (2020).

29. S. v. Schaik, *Tibet: A History* (Yale University Press, 2011), pp. 352.

30. V. M. Narasimhan, N. Patterson, P. Moorjani, N. Rohland, R. Bernardos, S. Mallick, I. Lazaridis, N. Nakatsuka, I. Olalde, M. Lipson, A. M. Kim, L. M. Olivieri, A. Coppa, M. Vidale, J. Mallory, V. Moiseyev, E. Kitov, J. Monge, N. Adamski, N. Alex, N. Broomandkhoshbacht, F. Candilio, K. Callan, O. Cheronet, B. J. Culleton, M. Ferry, D. Fernandes, S. Freilich, B. Gamarra, D. Gaudio, M. Hajdinjak, E. Harney, T. K. Harper, D. Keating, A. M. Lawson, M. Mah, K. Mandl, M. Michel, M. Novak, J. Oppenheimer, N. Rai, K. Sirak, V. Slon, K. Stewardson, F. Zalzala, Z. Zhang, G. Akhatov, A. N. Bagashev, A. Bagnera, B. Baitanayev, J. Bendezu-Sarmiento, A. A. Bissembaev, G. L. Bonora, T. T. Chargynov, T. Chikisheva, P. K. Dashkovskiy, A. Derevianko, M. Dobes, K. Douka, N. Dubova, M. N. Duisengali, D. Enshin, A. Epimakhov, A. V. Fribus, D. Fuller, A. Goryachev, A. Gromov, S. P. Grushin, B. Hanks, M. Judd, E. Kazizov, A. Khokhlov, A. P. Krygin, E. Kupriyanova, P. Kuznetsov, D. Luiselli, F. Maksudov, A. M. Mamedov, T. B. Mamirov, C. Meiklejohn, D. C. Merrett, R. Micheli, O. Mochalov, S. Mustafokulov, A. Nayak, D. Pettener, R. Potts, D. Razhev, M. Rykun, S. Sarno, T. M. Savenkova, K. Sikhymbaeva, S. M. Slepchenko, O. A. Soltobaev, N. Stepanova, S. Svyatko, K. Tabaldiev, M. Teschler-Nicola, A. A. Tishkin, V. V. Tkachev, S. Vasilyev, P. Veleminsky, D. Voyakin, A. Yermolayeva, M. Zahir, V. S. Zubkov, A. Zubova, V. S. Shinde, C. Lalueza-Fox, M. Meyer, D. Anthony, N. Boivin, K. Thangaraj, D. J. Kennett, M. Frachetti, R. Pinhasi, D. Reich, The formation of human populations in South and Central Asia. *Science* **365**, eaat7487 (2019).

31. X. Zhang, K. E. Witt, M. M. Banuelos, A. Ko, K. Yuan, S. Xu, R. Nielsen, E. Huerta-Sanchez, The history and evolution of the Denisovan-*EPAS1* haplotype in Tibetans. *Proc. Natl. Acad. Sci. U.S.A.* **118**, e2020803118 (2021).

32. E. Huerta-Sanchez, X. Jin, Asan, Z. Bianba, B. M. Peter, N. Vinckenbosch, Y. Liang, X. Yi, M. He, M. Somel, P. Ni, B. Wang, X. Ou, Huasang, J. Luosang, Z. X. Cuo, K. Li, G. Gao, Y.

Yin, W. Wang, X. Zhang, X. Xu, H. Yang, Y. Li, J. Wang, J. Wang, R. Nielsen, Altitude adaptation in Tibetans caused by introgression of Denisovan-like DNA. *Nature* **512**, 194–197 (2014).

33. E. Huerta-Sanchez, F. P. Casey, Archaic inheritance: Supporting high-altitude life in Tibet. *J. Appl. Physiol.* **119**, 1129–1134 (2015).

34. C. Leipe, T. Long, E. A. Sergusheva, Discontinuous spread of millet agriculture in eastern Asia and prehistoric population dynamics. *Sci. Adv.* **5**, eaax6225 (2019).

35. C. J. Stevens, D. Q. Fuller, The spread of agriculture in eastern Asia. *Lang. Dyn. Chang.* **7**, 152–186 (2017).

36. M. A. Yang, X. Fan, B. Sun, Ancient DNA indicates human population shifts and admixture in northern and southern China. *Science* **369**, 282–288 (2020).

37. G. Dong, X. Jia, R. Elston, F. Chen, S. Li, L. Wang, L. Cai, C. An, Spatial and temporal variety of prehistoric human settlement and its influencing factors in the upper Yellow River valley, Qinghai Province, China, *J. Archaeol. Sci.* **40**, 2538–2546 (2013).

38. J. V. Bellezza, *The Dawn of Tibet* (Rowman & Littlefield Publishers, 2014).

39. W. Huo, Tubo archaeology and Tubo civilization. *J. Tibet Univ.* **24**, 57–74 (2009).

40. H. L. Lu, Z. Y. Li, C. L. Ciren, D. D. Cao, X. Gao, L. H. Li, Sding Chung: An early multi-burial cave site on the Tibetan Plateau. *Antiquity* **96**, 745–753 (2022).

41. G. Zhaxi, A research review on the Zhang-zhung civilization in the past three decades. *J. Sichuan Minzu Coll.* **26**, (2017).

42. H. Lu, J. Zhang, Y. Yang, X. Yang, B. Xu, W. Yang, T. Tong, S. Jin, C. Shen, H. Rao, X. Li, H. Lu, D. Q. Fuller, L. Wang, C. Wang, D. Xu, N. Wu, Earliest tea as evidence for one branch of the silk road across the Tibetan Plateau. *Sci. Rep.* **6**, 18955 (2016).

43. J. Dabney, M. Knapp, I. Glocke, M. T. Gansauge, A. Weihmann, B. Nickel, C. Valdiosera, N. Garcia, S. Paabo, J. L. Arsuaga, M. Meyer, Complete mitochondrial genome sequence of a middle Pleistocene cave bear reconstructed from ultrashort DNA fragments. *Proc. Natl. Acad. Sci. U.S.A.* **110**, 15758–15763 (2013).

44. M. Meyer, M. Kircher, M. T. Gansauge, H. Li, F. Racimo, S. Mallick, J. G. Schraiber, F. Jay, K. Prufer, C. de Filippo, P. H. Sudmant, C. Alkan, Q. Fu, R. Do, N. Rohland, A. Tandon, M. Siebauer, R. E. Green, K. Bryc, A. W. Briggs, U. Stenzel, J. Dabney, J. Shendure, J. Kitzman, M. F. Hammer, M. V. Shunkov, A. P. Derevianko, N. Patterson, A. M. Andres, E. E. Eichler, M. Slatkin, D. Reich, J. Kelso, S. Paabo, A high-coverage genome sequence from an archaic Denisovan individual. *Science* **338**, 222–226 (2012).

45. M. T. Gansauge, M. Meyer, Single-stranded DNA library preparation for the sequencing of ancient or damaged DNA. *Nat. Protoc.* **8**, 737–748 (2013).

46. M. Meyer, M. Kircher, Illumina sequencing library preparation for highly multiplexed target capture and sequencing. *Cold Spring Harb. Protoc.* **2010**, pdb.prot5448 (2010).

47. M. Kircher, S. Sawyer, M. Meyer, Double indexing overcomes inaccuracies in multiplex sequencing on the Illumina platform. *Nucleic Acids Res.* **40**, e3 (2012).

48. Q. Fu, M. Meyer, X. Gao, U. Stenzel, H. A. Burbano, J. Kelso, S. Pääbo, DNA analysis of an early modern human from Tianyuan cave, China, *Proc. Natl. Acad. Sci. U.S.A.* **110**, 2223–2227 (2013).

49. Q. Fu, M. Hajdinjak, O. T. Moldovan, S. Constantin, S. Mallick, P. Skoglund, N. Patterson, N. Rohland, I. Lazaridis, B. Nickel, B. Viola, K. Prufer, M. Meyer, J. Kelso, D. Reich, S. Paabo, An early modern human from Romania with a recent Neanderthal ancestor. *Nature* **524**, 216–219 (2015).

50. G. Renaud, U. Stenzel, J. Kelso, leeHom: Adaptor trimming and merging for Illumina sequencing reads. *Nucleic Acids Res.* **42**, e141 (2014).

51. H. Li, R. Durbin, Fast and accurate short read alignment with Burrows-Wheeler transform. *Bioinformatics* **25**, 1754–1760 (2009).

52. Q. Fu, A. Mittnik, P. L. F. Johnson, K. Bos, M. Lari, R. Bollongino, C. Sun, L. Giemsch, R. Schmitz, J. Burger, A. M. Ronchitelli, F. Martini, R. G. Cremonesi, J. Svoboda, P. Bauer, D. Caramelli, S. Castellano, D. Reich, S. Paabo, J. Krause, A revised timescale for human evolution based on ancient mitochondrial genomes. *Curr. Biol.* **23**, 553–559 (2013).

53. T. S. Korneliussen, A. Albrechtsen, R. Nielsen, ANGSD: Analysis of next generation sequencing data. *BMC Bioinformatics* **15**, 356 (2014).

54. P. J. Reimer, W. E. N. Austin, E. Bard, The IntCal20 northern hemisphere radiocarbon age calibration curve (0–55 cal kBP). *Radiocarbon* **62**, 725–757 (2020).

55. P. Skoglund, J. Storå, A. Götherström, M. Jakobsson, Accurate sex identification of ancient human remains using DNA shotgun sequencing. *J. Archaeol. Sci.* **40**, 4477–4482 (2013).

56. M. van Oven, M. Kayser, Updated comprehensive phylogenetic tree of global human mitochondrial DNA variation. *Hum. Mutat.* **30**, E386–E394 (2009).

57. H. Weissensteiner, D. Pacher, A. Kloss-Brandstätter, L. Forer, G. Specht, H.-J. Bandelt, F. Kronenberg, A. Salas, S. Schönherr, HaploGrep 2: Mitochondrial haplogroup classification in the era of high-throughput sequencing. *Nucleic Acids Res.* **44**, W58–W63 (2016).

58. M. S. Blouin, DNA-based methods for pedigree reconstruction and kinship analysis in natural populations. *Trends Ecol. Evol.* **18**, 503–511 (2003).

59. S. Mallick, H. Li, M. Lipson, I. Mathieson, M. Gymrek, F. Racimo, M. Zhao, N. Chennagiri, S. Nordenfelt, A. Tandon, P. Skoglund, I. Lazaridis, S. Sankararaman, Q. Fu, N. Rohland, G. Renaud, Y. Erlich, T. Willems, C. Gallo, J. P. Spence, Y. S. Song, G. Poletti, F. Balloux, G. van Driem, P. de Knijff, I. G. Romero, A. R. Jha, D. M. Behar, C. M. Bravi, C. Capelli, T. Hervig, A. Moreno-Estrada, O. L. Posukh, E. Balanovska, O. Balanovsky, S. Karachanak-Yankova, H. Sahakyan, D. Toncheva, L. Yepiskoposyan, C. Tyler-Smith, Y. Xue, M. S. Abdullah, A. Ruiz-Linares, C. M. Beall, A. Di Rienzo, C. Jeong, E. B.

Starikovskaya, E. Metspalu, J. Parik, R. Villems, B. M. Henn, U. Hodoglugil, R. Mahley, A. Sajantila, G. Stamatoyannopoulos, J. T. S. Wee, R. Khusainova, E. Khusnutdinova, S. Litvinov, G. Ayodo, D. Comas, M. F. Hammer, T. Kivisild, W. Klitz, C. A. Winkler, D. Labuda, M. Bamshad, L. B. Jorde, S. A. Tishkoff, W. S. Watkins, M. Metspalu, S. Dryomov, R. Sukernik, L. Singh, K. Thangaraj, S. Pääbo, J. Kelso, N. Patterson, D. Reich, The Simons genome diversity project: 300 genomes from 142 diverse populations. *Nature* **538**, 201–206 (2016).

60. S. Purcell, B. Neale, K. Todd-Brown, L. Thomas, M. A. Ferreira, D. Bender, J. Maller, P. Sklar, P. I. De Bakker, M. J. Daly, PLINK: A tool set for whole-genome association and population-based linkage analyses. *Am. J. Hum. Genet.* **81**, 559–575 (2007).

61. P. Skoglund, C. Posth, K. Sirak, M. Spriggs, F. Valentin, S. Bedford, G. R. Clark, C. Reepmeyer, F. Petchey, D. Fernandes, Q. Fu, E. Harney, M. Lipson, S. Mallick, M. Novak, N. Rohland, K. Stewardson, S. Abdullah, M. P. Cox, F. R. Friedlaender, J. S. Friedlaender, T. Kivisild, G. Koki, P. Kusuma, D. A. Merriwether, F. X. Ricaut, J. T. Wee, N. Patterson, J. Krause, R. Pinhasi, D. Reich, Genomic insights into the peopling of the Southwest Pacific. *Nature* **538**, 510–513 (2016).

62. P. Damgaard, N. Marchi, S. Rasmussen, M. Peyrot, G. Renaud, T. Korneliussen, J. V. Moreno-Mayar, M. W. Pedersen, A. Goldberg, E. Usmanova, N. Baimukhanov, V. Loman, L. Hedeager, A. G. Pedersen, K. Nielsen, G. Afanasiev, K. Akmatov, A. Aldashev, A. Alpaslan, G. Baimbetov, V. I. Bazaliiskii, A. Beisenov, B. Boldbaatar, B. Boldgiv, C. Dorzhu, S. Ellingvag, D. Erdenebaatar, R. Dajani, E. Dmitriev, V. Evdokimov, K. M. Frei, A. Gromov, A. Goryachev, H. Hakonarson, T. Hegay, Z. Khachatryan, R. Khaskhanov, E. Kitov, A. Kolbina, T. Kubatbek, A. Kukushkin, I. Kukushkin, N. Lau, A. Margaryan, I. Merkyte, I. V. Mertz, V. K. Mertz, E. Mijiddorj, V. Moiyesev, G. Mukhtarova, B. Nurmukhanbetov, Z. Orozbekova, I. Panyushkina, K. Pieta, V. Smrcka, I. Shevnina, A. Logvin, K. G. Sjogren, T. Stolcova, K. Tashbaeva, A. Tkachev, T. Tulegenov, D. Voyakin, L. Yepiskoposyan, S. Undrakhbold, V. Varfolomeev, A. Weber, N. Kradin, M. E. Allentoft, L. Orlando, R. Nielsen, M. Sikora, E. Heyer, K. Kristiansen, E. Willerslev, 137 ancient human genomes from across the Eurasian steppes. *Nature* **557**, 369–374 (2018).

63. P. Damgaard, R. Martiniano, J. Kamm, J. V. Moreno-Mayar, G. Kroonen, M. Peyrot, G. Barjamovic, S. Rasmussen, C. Zacho, N. Baimukhanov, V. Zaibert, V. Merz, A. Biddanda, I. Merz, V. Loman, V. Evdokimov, E. Usmanova, B. Hemphill, A. Seguin-Orlando, F. E. Yediay, I. Ullah, K. G. Sjogren, K. H. Iversen, J. Choin, C. de la Fuente, M. Ilardo, H. Schroeder, V. Moiseyev, A. Gromov, A. Polyakov, S. Omura, S. Y. Senyurt, H. Ahmad, C. McKenzie, A. Margaryan, A. Hameed, A. Samad, N. Gul, M. H. Khokhar, O. I. Goriunova, V. I. Bazaliiskii, J. Novembre, A. W. Weber, L. Orlando, M. E. Allentoft, R. Nielsen, K. Kristiansen, M. Sikora, A. K. Outram, R. Durbin, E. Willerslev, The first horse herders and the impact of early bronze age steppe expansions into Asia. *Science* **360**, eaar7711 (2018).

64. M. Rasmussen, S. L. Anzick, M. R. Waters, P. Skoglund, M. DeGiorgio, T. W. Stafford Jr., S. Rasmussen, I. Moltke, A. Albrechtsen, S. M. Doyle, G. D. Poznik, V. Gudmundsdottir, R. Yadav, A.-S. Malaspinas, S. S. White, M. E. Allentoft, O. E. Cornejo, K. Tambets, A. Eriksson, P. D. Heintzman, M. Karmin, T. S. Korneliussen, D. J. Meltzer, T. L. Pierre, J. Stenderup, L. Saag, V. M. Warmuth, M. C. Lopes, R. S. Malhi, S. Brunak, T. Sicheritz-Ponten, I. Barnes, M. Collins, L. Orlando, F. Balloux, *A. Manica*, R. Gupta, M. Metspalu, C. D. Bustamante, M. Jakobsson, R. Nielsen, E. Willerslev, The genome of a Late Pleistocene human from a Clovis burial site in western Montana. *Nature* **506**, 225–229 (2014).

65. C. Ning, T. Li, K. Wang, F. Zhang, T. Li, X. Wu, S. Gao, Q. Zhang, H. Zhang, M. J. Hudson, G. Dong, S. Wu, Y. Fang, C. Liu, C. Feng, W. Li, T. Han, R. Li, J. Wei, Y. Zhu, Y. Zhou, C. C. Wang, S. Fan, Z. Xiong, Z. Sun, M. Ye, L. Sun, X. Wu, F. Liang, Y. Cao, X. Wei, H. Zhu, H. Zhou, J. Krause, M. Robbeets, C. Jeong, Y. Cui, Ancient genomes from northern China suggest links between subsistence changes and human migration. *Nat. Commun.* **11**, 2700 (2020).

66. É. Harney, N. Patterson, D. Reich, J. Wakeley, Assessing the performance of qpAdm: A statistical tool for studying population admixture. *bioRxiv*, 2020.04.09.032664 (2020).

67. B. M. Peter, 100,000 years of gene flow between Neandertals and Denisovans in the Altai mountains. *bioRxiv*, 2020.03.13.990523 (2020).

68. K. Prufer, F. Racimo, N. Patterson, F. Jay, S. Sankararaman, S. Sawyer, A. Heinze, G. Renaud, P. H. Sudmant, C. de Filippo, H. Li, S. Mallick, M. Dannemann, Q. Fu, M. Kircher, M. Kuhlwilm, M. Lachmann, M. Meyer, M. Ongyerth, M. Siebauer, C. Theunert, A. Tandon, P. Moorjani, J. Pickrell, J. C. Mullikin, S. H. Vohr, R. E. Green, I. Hellmann, P. L. Johnson, H. Blanche, H. Cann, J. O. Kitzman, J. Shendure, E. E. Eichler, E. S. Lein, T. E. Bakken, L. V. Golovanova, V. B. Doronichev, M. V. Shunkov, A. P. Derevianko, *B. Viola*, M. Slatkin, D. Reich, J. Kelso, S. Paabo, The complete genome sequence of a Neanderthal from the Altai Mountains. *Nature* **505**, 43–49 (2014).

69. K. Prüfer, C. d. Filippo, S. Grote, F. Mafessoni, A high-coverage Neandertal genome from Vindija cave in Croatia. *Science* **358**, 4 (2017).

70. Y. Wang, F. Song, J. Zhu, S. Zhang, Y. Yang, T. Chen, B. Tang, L. Dong, N. Ding, Q. Zhang, Z. Bai, X. Dong, H. Chen, M. Sun, S. Zhai, Y. Sun, L. Yu, L. Lan, J. Xiao, X. Fang, H. Lei, Z. Zhang, W. Zhao, GSA: Genome sequence archive. *Genomics Proteomics Bioinformatics* **15**, 14–18 (2017).

71. Big Data Center Members, Database resources of the BIG data center in 2018. *Nucleic Acids Res.* **46**, D14–D20 (2018).

72. H. H. Chen, “A study of the Zongri remains,” thesis, Peking University (Chinese) (1992).

73. X. Guo, “The study of burial potteries from Zongri relic,” thesis, Xibei University (Chinese) (2014).

74. Y. Liu, “Archaeobotanical study in Zongri Site, Qinghai Province, China,” thesis, Lanzhou University (Chinese) (2018).

75. X. Y. Ren, Y. H. He, Q. W. Zhao, S. C. Pan, M. Tang, J. Y. Cao, C. Huang, H. Jiang, D. Tan, Y. M. Wang, L. H. Cai, X. J. Gu, Y. Song, Y. Qin, W. Du, C. Y. Ma, P. Li, D. J. Lu, Y. X. Zheng, X. H. Yu, The brief survery report of Pukagongma sarcophagus tomb in Zhiduo County, Qinghai Province. *J. Tibet.* **2017**, 24–40 (2017).

76. L. L. Ren, G. H. Dong, H. M. Li, D. Rhode, R. K. Flad, G. Q. Li, Y. Yang, Z. X. Wang, L. H. Cai, X. Y. Ren, D. J. Zhang, F. H. Chen, Dating human settlement in the east-central Tibetan Plateau during the Late Holocene. **60**, 137–150 (2018).

77. Z. Zhang, W. Xiage, H. Lv, C. n. Sodnam, Identification and interpretation of faunal remains from a prehistoric cist burial in Amdo County, North Tibet. *Journal of Tibetology (Chinese)* 1–18 (2015).

78. W. Xiage, "Unveiling the veil of ancient civilization in Qiangtang Plateau," *Journal of Tibet University (Chinese)* v01.20.NO.1, (2005).

79. Xiaoyong, "Remains of tombs from the early 7th century to the early 8th century were found in bange County, Tibet Autonomous Region," *Tibet Daily (Chinese)* (2019).

80. X. M. C. H. Bureau, D. o. A. o. S. University, S. P. I. o. Archaeology, *Field Archaeology Report of Tibetan Section of Qinghai-Tibet Railway* (Science Press (Chinese), 2005).

81. H. Wang, Y. Li, Y. Feng, L. Tu, H. Zhang, The age of human bones from Redilong, Qamdo County, Tibet. *Acta Geoscinet. Sin.* **24**, 569–572 (2003).

82. W. Xiage, Tentative analysis of the type and age of the prehistoric stone coffin burials in Tibet. *Tibet Stud.*, 40–44 (1998).

83. S. t. o. t. X. M. A. C. o. Cultural, The preliminary report of Xiaoenda Neolithic age ruins in Xizang autonomous region. 1, (1990).

84. Anonymous, "Agongrong cemetery in Bomi, Tibet.," *Popular Archaeology (Chinese)* v4 (2017).

85. Xinhuanet, "The largest number of pottery tombs unearthed in Shannan, Tibet," *Identification and Appreciation to Cultural Relics (Chinese)* v6 (2018).

86. Z. D. S. He, Wangdui, "Discovery of ancient tombs in Jiesang village, Naidong, Tibet," *Archaeology (Chinese)* v12 (1985).

87. Z. Luobu, “New discovery and preliminary understanding of early tombs in central Tibet,” *The second silk road archaeological Forum (Chinese Meeting)* (2018).

88. F. B. Jiang, “Middle and late Tubo tombs discovered in Tibet,” *China News Service (Chinese)* (2018).

89. Archaeological investigation and excavation report of water control project in Pundo County Linzhou City, in *Research on Cultural Relics and Archaeology in Tibet*, Habibu, F. Xu, L. Dunzhu, Eds. (Science Press, ed. 1, 2016), chap. 1, pp. 282.

90. H. Wei, “Archaeological discovery and research of prehistoric tombs in Tibet plateau,” *China Tibet* v4 (1994).

91. S. Wang, “Archaeological discoveries “outline” the prehistoric civilization of Western Tibet,” *China Tibet Online (Chinese)* (2018).

92. X. Yu, “Archaeological discoveries and characteristics of funeral customs in Western Tibet in the pre Tubo era,” *Journal of Northwest Minzu University* 1 (2017).

93. Y. X. Li, “A brief report on the investigation of Gebusailu cemetery in Zhada County, Tibet,” *Archaeology (Chinese)* v6 (2001).

94. J. H. Yao, W, “A brief report on the excavation of ancient tombs at the piyang Dongga site in Zhada County, Tibet,” *Archaeology (Chinese)* v6 (2001).

95. A. Bergstrom, S. A. McCarthy, R. Hui, M. A. Almarri, Q. Ayub, P. Danecek, Y. Chen, S. Felkel, P. Hallast, J. Kamm, H. Blanche, J. F. Deleuze, H. Cann, S. Mallick, D. Reich, M. S. Sandhu, P. Skoglund, A. Scally, Y. Xue, R. Durbin, C. Tyler-Smith, Insights into human genetic variation and population history from 929 diverse genomes. *Science* **367**, eaay5012 (2020).

96. V. M. Narasimhan, N. Patterson, P. Moorjani, I. Lazaridis, L. Mark, S. Mallick, N. Rohland, R. Bernardos, A. Kim, N. Nakatsuka, I. Olalde, A. Coppa, J. Mallory, V. Moiseyev, J. Monge, L. Olivieri, N. Adamski, N. Broomandkhoshbacht, F. Candilio, O. Cheronet, B.

Culleton, M. Ferry, D. Fernandes, B. Gamarra, D. Gaudio, M. Hajdinjak, E. Harney, T. Harper, D. Keating, A. Lawson, M. Michel, M. Novak, J. Oppenheimer, N. Rai, K. Sirak, V. Slon, K. Stewardson, Z. Zhang, G. Akhatov, A. Bagashev, B. Baitanayev, G. Bonora, T. Chikisheva, A. Derevianko, E. Dmitry, K. Douka, N. Dubova, A. Epimakhov, S. Freilich, D. Fuller, A. Goryachev, A. Gromov, B. Hanks, M. Judd, E. Kazizov, A. Khokhlov, E. Kitov, E. Kupriyanova, P. Kuznetsov, D. Luiselli, F. Maksudov, C. Meiklejohn, D. Merrett, R. Micheli, O. Mochalov, Z. Muhammed, S. Mustafakulov, A. Nayak, R. Petrovna, D. Pettner, R. Potts, D. Razhev, S. Sarno, K. Sikhymbaevae, S. Slepchenko, N. Stepanova, S. Svyatko, S. Vasilyev, M. Vidale, D. Voyakin, A. Yermolayeva, A. Zubova, V. Shinde, C. Lalueza-Fox, M. Meyer, D. Anthony, N. Boivin, K. Thangaraj, D. Kennett, M. Frachetti, R. Pinhasi, D. Reich, The genomic formation of South and Central Asia. bioRxiv 292581 [**Preprint**]. 31 March 2018. https://doi.org/10.1101/292581.

97. H. McColl, F. Racimo, L. Vinner, F. Demeter, T. Gakuhari, J. V. Moreno-Mayar, G. van Driem, U. Gram Wilken, A. Seguin-Orlando, C. de la Fuente Castro, S. Wasef, R. Shoocongdej, V. Souksavatdy, T. Sayavongkhamdy, M. M. Saidin, M. E. Allentoft, T. Sato, A. S. Malaspinas, F. A. Aghakhanian, T. Korneliussen, A. Prohaska, A. Margaryan, P. de Barros Damgaard, S. Kaewsutthi, P. Lertrit, T. M. H. Nguyen, H. C. Hung, T. Minh Tran, H. Nghia Truong, G. H. Nguyen, S. Shahidan, K. Wiradnyana, H. Matsumae, N. Shigehara, M. Yoneda, H. Ishida, T. Masuyama, Y. Yamada, A. Tajima, H. Shibata, A. Toyoda, T. Hanihara, S. Nakagome, T. Deviese, A. M. Bacon, P. Duringer, J. L. Ponche, L. Shackelford, E. Patole-Edoumba, A. T. Nguyen, B. Bellina-Pryce, J. C. Galipaud, R. Kinaston, H. Buckley, C. Pottier, S. Rasmussen, T. Higham, R. A. Foley, M. M. Lahr, L. Orlando, M. Sikora, M. E. Phipps, H. Oota, C. Higham, D. M. Lambert, E. Willerslev, The prehistoric peopling of Southeast Asia. *Science* **361**, 88–92 (2018).

98. T. Wang, W. Wang, G. Xie, Human population history at the crossroads of east and Southeast Asia since 11,000 years ago. *Cell* **184**, 3829–3841.e21 (2021).

99. P. Skoglund, J. C. Thompson, M. E. Prendergast, A. Mittnik, K. Sirak, M. Hajdinjak, T. Salie, N. Rohland, S. Mallick, A. Peltzer, A. Heinze, I. Olalde, M. Ferry, E. Harney, M. Michel, K. Stewardson, J. I. Cerezo-Roman, C. Chiumia, A. Crowther, E. Gomani-

Chindebvu, A. O. Gidna, K. M. Grillo, I. T. Helenius, G. Hellenthal, R. Helm, M. Horton, S. Lopez, A. Z. P. Mabulla, J. Parkington, C. Shipton, M. G. Thomas, R. Tibesasa, M. Welling, V. M. Hayes, D. J. Kennett, R. Ramesar, M. Meyer, S. Paabo, N. Patterson, A. G. Morris, N. Boivin, R. Pinhasi, J. Krause, D. Reich, Reconstructing prehistoric African population structure. *Cell* **171**, 59–71.e21 (2017).

100. I. Lazaridis, N. Patterson, A. Mittnik, G. Renaud, S. Mallick, K. Kirsanow, P. H. Sudmant, J. G. Schraiber, S. Castellano, M. Lipson, B. Berger, C. Economou, R. Bollongino, Q. Fu, K. I. Bos, S. Nordenfelt, H. Li, C. de Filippo, K. Prufer, S. Sawyer, C. Posth, W. Haak, F. Hallgren, E. Fornander, N. Rohland, D. Delsate, M. Francken, J. M. Guinet, J. Wahl, G. Ayodo, H. A. Babiker, G. Bailliet, E. Balanovska, O. Balanovsky, R. Barrantes, G. Bedoya, H. Ben-Ami, J. Bene, F. Berrada, C. M. Bravi, F. Brisighelli, G. B. Busby, F. Cali, M. Churnosov, D. E. Cole, D. Corach, L. Damba, G. van Driem, S. Dryomov, J. M. Dugoujon, S. A. Fedorova, I. Gallego Romero, M. Gubina, M. Hammer, B. M. Henn, T. Hervig, U. Hodoglugil, A. R. Jha, S. Karachanak-Yankova, R. Khusainova, E. Khusnutdinova, R. Kittles, T. Kivisild, W. Klitz, V. Kucinskas, A. Kushniarevich, L. Laredj, S. Litvinov, T. Loukidis, R. W. Mahley, B. Melegh, E. Metspalu, J. Molina, J. Mountain, K. Nakkalajarvi, D. Nesheva, T. Nyambo, L. Osipova, J. Parik, F. Platonov, O. Posukh, V. Romano, F. Rothhammer, I. Rudan, R. Ruizbakiev, H. Sahakyan, A. Sajantila, A. Salas, E. B. Starikovskaya, A. Tarekegn, D. Toncheva, S. Turdikulova, I. Uktveryte, O. Utevska, R. Vasquez, M. Villena, M. Voevoda, C. A. Winkler, L. Yepiskoposyan, P. Zalloua, T. Zemunik, A. Cooper, C. Capelli, M. G. Thomas, A. Ruiz-Linares, S. A. Tishkoff, L. Singh, K. Thangaraj, R. Villems, D. Comas, R. Sukernik, M. Metspalu, M. Meyer, E. E. Eichler, J. Burger, M. Slatkin, S. Paabo, J. Kelso, D. Reich, J. Krause, Ancient human genomes suggest three ancestral populations for present-day Europeans. *Nature* **513**, 409–413 (2014).

101. S. R. Grossman, I. Shlyakhter, E. K. Karlsson, E. H. Byrne, S. Morales, G. Frieden, E. Hostetter, E. Angelino, M. Garber, O. Zuk, E. S. Lander, S. F. Schaffner, P. C. Sabeti, A composite of multiple signals distinguishes causal variants in regions of positive selection. *Science* **327**, 883–886 (2010).

102. A. Fujimoto, J. Ohashi, N. Nishida, T. Miyagawa, Y. Morishita, T. Tsunoda, R. Kimura, K. Tokunaga, A replication study confirmed the EDAR gene to be a major contributor to population differentiation regarding head hair thickness in Asia. *Hum. Genet.* **124**, 179–185 (2008).

103. Y. G. Kamberov, S. Wang, J. Tan, P. Gerbault, A. Wark, L. Tan, Y. Yang, S. Li, K. Tang, H. Chen, A. Powell, Y. Itan, D. Fuller, J. Lohmueller, J. Mao, A. Schachar, M. Paymer, E. Hostetter, E. Byrne, M. Burnett, A. P. McMahon, M. G. Thomas, D. E. Lieberman, L. Jin, C. J. Tabin, B. A. Morgan, P. C. Sabeti, Modeling recent human evolution in mice by expression of a selected EDAR variant. *Cell* **152**, 691–702 (2013).

104. J. H. Park, T. Yamaguchi, C. Watanabe, A. Kawaguchi, K. Haneji, M. Takeda, Y. I. Kim, Y. Tomoyasu, M. Watanabe, H. Oota, T. Hanihara, H. Ishida, K. Maki, S. B. Park, R. Kimura, Effects of an Asian-specific nonsynonymous EDAR variant on multiple dental traits. *J. Hum. Genet.* **57**, 508–514 (2012).

105. R. A. Sturm, D. L. Duffy, Z. Z. Zhao, F. P. Leite, M. S. Stark, N. K. Hayward, N. G. Martin, G. W. Montgomery, A single SNP in an evolutionary conserved region within intron 86 of the HERC2 gene determines human blue-brown eye color. *Am. J. Hum. Genet.* **82**, 424–431 (2008).

106. C. A. Guenther, B. Tasic, L. Luo, M. A. Bedell, D. M. Kingsley, A molecular basis for classic blond hair color in Europeans. *Nat. Genet.* **46**, 748–752 (2014).

107. F. Liu, K. van Duijn, J. R. Vingerling, A. Hofman, A. G. Uitterlinden, A. C. Janssens, M. Kayser, Eye color and the prediction of complex phenotypes from genotypes. *Curr. Biol.* **19**, R192–R193 (2009).

108. F. Liu, A. Wollstein, P. G. Hysi, G. A. Ankra-Badu, T. D. Spector, D. Park, G. Zhu, M. Larsson, D. L. Duffy, G. W. Montgomery, D. A. Mackey, S. Walsh, O. Lao, A. Hofman, F. Rivadeneira, J. R. Vingerling, A. G. Uitterlinden, N. G. Martin, C. J. Hammond, M. Kayser, Digital quantification of human eye color highlights genetic association of three new loci. *PLOS Genet.* **6**, e1000934 (2010).

109. R. L. Lamason, M. A. Mohideen, J. R. Mest, A. C. Wong, H. L. Norton, M. C. Aros, M. J. Jurynec, X. Mao, V. R. Humphreville, J. E. Humbert, S. Sinha, J. L. Moore, P. Jagadeeswaran, W. Zhao, G. Ning, I. Makalowska, P. M. McKeigue, D. O'Donnell, R. Kittles, E. J. Parra, N. J. Mangini, D. J. Grunwald, M. D. Shriver, V. A. Canfield, K. C. Cheng, SLC24A5, a putative cation exchanger, affects pigmentation in zebrafish and humans. *Science* **310**, 1782–1786 (2005).

110. M. Soejima, Y. Koda, Population differences of two coding SNPs in pigmentation-related genes SLC24A5 and SLC45A2. *Int. J. Leg. Med.* **121**, 36–39 (2007).

111. S. Walsh, F. Liu, A. Wollstein, L. Kovatsi, A. Ralf, A. Kosiniak-Kamysz, W. Branicki, M. Kayser, The HIrisPlex system for simultaneous prediction of hair and eye colour from DNA. *Forensic Sci. Int. Genet.* **7**, 98–115 (2013).

112. P. Sulem, D. F. Gudbjartsson, S. N. Stacey, A. Helgason, T. Rafnar, K. P. Magnusson, A. Manolescu, A. Karason, A. Palsson, G. Thorleifsson, M. Jakobsdottir, S. Steinberg, S. Palsson, F. Jonasson, B. Sigurgeirsson, K. Thorisdottir, R. Ragnarsson, K. R. Benediktsdottir, K. K. Aben, L. A. Kiemeney, J. H. Olafsson, J. Gulcher, A. Kong, U. Thorsteinsdottir, K. Stefansson, Genetic determinants of hair, eye and skin pigmentation in Europeans. *Nat. Genet.* **39**, 1443–1452 (2007).

113. N. S. Enattah, T. Sahi, E. Savilahti, J. D. Terwilliger, L. Peltonen, I. Jarvela, Identification of a variant associated with adult-type hypolactasia. *Nat. Genet.* **30**, 233–237 (2002).

114. C. J. Ingram, M. F. Elamin, C. A. Mulcare, M. E. Weale, A. Tarekegn, T. O. Raga, E. Bekele, F. M. Elamin, M. G. Thomas, N. Bradman, D. M. Swallow, A novel polymorphism associated with lactose tolerance in Africa: Multiple causes for lactase persistence? *Hum. Genet.* **120**, 779–788 (2007).

115. S. A. Tishkoff, F. A. Reed, A. Ranciaro, B. F. Voight, C. C. Babbitt, J. S. Silverman, K. Powell, H. M. Mortensen, J. B. Hirbo, M. Osman, M. Ibrahim, S. A. Omar, G. Lema, T. B. Nyambo, J. Ghori, S. Bumpstead, J. K. Pritchard, G. A. Wray, P. Deloukas, Convergent adaptation of human lactase persistence in Africa and Europe. *Nat. Genet.* **39**, 31–40 (2007).

116. K. Eaton, M. Edwards, S. Krithika, G. Cook, H. Norton, E. J. Parra, Association study confirms the role of two OCA2 polymorphisms in normal skin pigmentation variation in East Asian populations. *Am. J. Hum. Biol.* **27**, 520–525 (2015).

117. H. Rajeevan, U. Soundararajan, J. R. Kidd, A. J. Pakstis, K. K. Kidd, ALFRED: An allele frequency resource for research and teaching. *Nucleic Acids Res.* **40**, D1010–1015 (2012).

118. P. J. Brooks, M. A. Enoch, D. Goldman, T. K. Li, A. Yokoyama, The alcohol flushing response: An unrecognized risk factor for esophageal cancer from alcohol consumption. *PLOS Med.* **6**, e50 (2009).

119. A. Yoshida, I. Y. Huang, M. Ikawa, Molecular abnormality of an inactive aldehyde dehydrogenase variant commonly found in Orientals. *Proc. Natl. Acad. Sci. U.S.A.* **81**, 258–261 (1984).

120. H. Li, S. Borinskaya, K. Yoshimura, N. Kal'ina, A. Marusin, V. A. Stepanov, Z. Qin, S. Khaliq, M. Y. Lee, Y. Yang, A. Mohyuddin, D. Gurwitz, S. Q. Mehdi, E. Rogaev, L. Jin, N. K. Yankovsky, J. R. Kidd, K. K. Kidd, Refined geographic distribution of the oriental *ALDH2*504Lys* (nee *487Lys*) variant. *Ann. Hum. Genet.* **73**, 335–345 (2009).

121. H. Li, N. Mukherjee, U. Soundararajan, Z. Tarnok, C. Barta, S. Khaliq, A. Mohyuddin, S. L. Kajuna, S. Q. Mehdi, J. R. Kidd, K. K. Kidd, Geographically separate increases in the frequency of the derived ADH1B*47His allele in eastern and western Asia. *Am. J. Hum. Genet.* **81**, 842–846 (2007).

122. Y. Peng, H. Shi, X. B. Qi, C. J. Xiao, H. Zhong, R. L. Ma, B. Su, The ADH1B Arg47His polymorphism in east Asian populations and expansion of rice domestication in history. *BMC Evol. Biol.* **10**, 15 (2010).
